# Neural and psychosocial signatures of the comorbidity between pain and affective symptoms

**DOI:** 10.1097/PR9.0000000000001353

**Published:** 2025-12-12

**Authors:** Francesco Scarlatti, Ludovic Dormegny-Jeanjean, Roman Schefzik, Jack R. Foucher, Tobias Banaschewski, Arun L.W. Bokde, Rüdiger Brühl, Sylvane Desrivières, Hugh Garavan, Penny Gowland, Antoine Grigis, Andreas Heinz, Jean-Luc Martinot, Marie-Laure Paillère Martinot, Eric Artiges, Dimitri Papadopoulos Orfanos, Luise Poustka, Michael N. Smolka, Sarah Hohmann, Nilakshi Vaidya, Henrik Walter, Robert Whelan, Gunter Schumann, Frauke Nees, Emanuel Schwarz, Martin Löffler, Herta Flor, IMAGEN Consortium, Tobias Banaschewski

**Affiliations:** a Department of Neuropsychology and Psychological Resilience Research, Research Group Learning and Brain Plasticity in Mental Disorders, Central Institute of Mental Health, Medical Faculty Mannheim, Heidelberg University, Mannheim, Germany; b UMR CNRS 7357, Engineering Science, Computer Science and Imaging Laboratory (ICube), University of Strasbourg, Strasbourg, France; c Department of Elderly Psychiatry and Neurostimulation, University Hospitals of Strasbourg, University of Strasbourg, Strasbourg, France; d Hector Institute for Artificial Intelligence in Psychiatry, Central Institute of Mental Health, Medical Faculty Mannheim, Heidelberg University, Mannheim, Germany; e Department of Psychiatry and Psychotherapy, Central Institute of Mental Health, Medical Faculty Mannheim, Heidelberg University, Mannheim, Germany; f Department of Child and Adolescent Psychiatry and Psychotherapy, Central Institute of Mental Health, Medical Faculty Mannheim, Heidelberg University, Mannheim, Germany. German Center for Mental Health (DZPG), partner site Mannheim-Heidelberg-Ulm; g Discipline of Psychiatry, School of Medicine and Trinity College Institute of Neuroscience, Trinity College Dublin, Dublin, Ireland; h Physikalisch-Technische Bundesanstalt (PTB), Braunschweig and Berlin, Germany; i Social, Genetic and Developmental Psychiatry Centre, Institute of Psychiatry, Psychology & Neuroscience, King's College London, London, United Kingdom; j Departments of Psychiatry and Psychology, University of Vermont, Burlington, VT, USA; k Sir Peter Mansfield Imaging Centre School of Physics and Astronomy, University of Nottingham, Nottingham, United Kingdom; l NeuroSpin, CEA, Université Paris-Saclay, Gif-sur-Yvette, France; m Department of Psychiatry and Psychotherapy, University of Tübingen, Tübingen, Germany; German Center for Mental Health (DZPG), Site Tübingen, Germany; n Institut National de la Santé et de la Recherche Médicale, INSERM U A10 “Trajectoires développementales & psychiatrie,” University Paris-Saclay, Ecole Normale Supérieure Paris-Saclay, CNRS; Centre Borelli, Gif-sur-Yvette, France; o AP-HP, Sorbonne Université, Department of Child and Adolescent Psychiatry, Pitié-Salpêtrière Hospital, Paris, France; p Psychiatry Department, EPS Barthélémy Durand, Etampes, France; q Department of Psychology, School of Social Sciences, University of Mannheim, Mannheim, Germany; r Department of Child and Adolescent Psychiatry, Center for Psychosocial Medicine, University Hospital Heidelberg, Heidelberg, Germany; s Department of Psychiatry and Psychotherapy, Technische Universität Dresden, Dresden, Germany; t Centre for Population Neuroscience and Stratified Medicine (PONS), Department of Psychiatry and Psychotherapy, Charité Universitätsmedizin Berlin, Berlin, Germany; u Department of Psychiatry and Psychotherapy CCM, Charité – Universitätsmedizin Berlin, corporate member of Freie Universität Berlin, Humboldt-Universität zu Berlin, and Berlin Institute of Health, Berlin, Germany; v School of Psychology and Global Brain Health Institute, Trinity College Dublin, Dublin, Ireland; w Centre for Population Neuroscience and Precision Medicine (PONS), Institute for Science and Technology of Brain-inspired Intelligence (ISTBI), Fudan University, Shanghai, China; x German Center for Mental Health (DZPG), Site Berlin-Potsdam, Germany; y Institute of Medical Psychology and Medical Sociology, University Medical Center Schleswig Holstein, Kiel University, Kiel, Germany; z Clinical Psychology, Department of Experimental Psychology, Heinrich Heine University Düsseldorf, Düsseldorf, Germany

**Keywords:** Comorbidity, Pain, Depression, Anxiety, Reward, Markers, fMRI

## Abstract

**Introduction:**

The bidirectional relationship between pain and affective symptoms suggests shared underlying mechanisms. Neurophysiological changes, psychosocial factors, and specifically alterations in instrumental learning likely contribute significantly to their co-occurrence.

**Objectives:**

In this study, we aimed to uncover factors associated with the comorbidity between pain and the severity of depressive symptoms and between pain and the severity of anxiety symptoms.

**Methods:**

We analyzed psychosocial (n = 624) and neuroimaging (n = 689) data from a European sample of young adults with pain and affective symptoms from the general population. Using multitask learning, we constructed cross-sectional models predicting the coexistence of both symptom domains.

**Results:**

Based on blood-oxygen-level-dependent responses during reward feedback, we found a neural pattern associated with the comorbidity between pain and the severity of depressive symptoms, reflecting regionally heterogeneous alterations in reward responsivity. These alterations included stronger negative responses in the caudate nucleus and putamen. In addition, we identified robust psychosocial markers of the comorbidity between pain and the severity of either depressive or anxiety symptoms, among which nonpainful somatic symptoms and persistent rumination showed the most robust association. We further compared the predictive capabilities of psychosocial markers with those of neural processes in instrumental learning and found that neural signatures of reward feedback improved the prediction of a psychosocial signature for the comorbidity between pain and the severity of depressive symptoms.

**Conclusion:**

These findings highlight the presence of common mechanisms underlying the comorbidity of pain and affective symptoms, including alterations in the neural responses to reward.

## Abstract 2

Supplemental Digital Content is Available in the Text.

A neural signature of reward feedback and psychosocial signatures were associated with the comorbidity between pain and depressive symptoms, and between pain and anxiety symptoms.

## 1. Introduction

Pain often co-occurs with affective symptoms such as depression and anxiety,^9,26,43^ and their bidirectional relationship is associated with poorer outcomes and reduced treatment response.^3,4^ Identifying indicators and understanding the mechanisms underlying this comorbidity is therefore critical.

Altered instrumental learning, a process in which behavior is shaped by rewards and punishments, has been reported in individuals experiencing pain,^13–15,37^ depressive,^2,18,41,42^ and anxiety symptoms.^41,53^ These conditions are often characterized by reduced reinforcement of healthy behaviors and increased reinforcement of maladaptive ones.^2,13–15,21,37,41,42^ Although both depressive and anxiety symptoms have been linked to heightened punishment sensitivity, diminished reward sensitivity is most pronounced in depression.^7,17,25,42^ Reduced reward sensitivity and anhedonia have also been observed in individuals with persistent pain symptoms.^19,32,39,40,50^

Neurobiologically, pain and affective symptoms and disorders have been linked to overlapping alterations in the functioning of reward-related brain circuits, including the striatum, orbitofrontal cortex (OFC), and ventromedial prefrontal cortex (vmPFC).^1,27,30,42,48^ Psychosocial factors such as nonpainful somatic symptoms, rumination, neuroticism, and the experience of trauma further contribute to the persistence and interaction of these symptoms.^9,10,31,34^ For instance, reduced sensitivity to rewards may lead to diminished engagement in goal-directed activities and increased social withdrawal, both of which are core features of depression.^2,42,46^ They may also be implicated in lower activity levels, a recognized risk factor for the persistence of pain.^23,29^ By contrast, increased sensitivity to punishments may induce avoidance of excessively feared experiences, a hallmark of anxiety,^53^ as well as maladaptive avoidant responses to pain (pain behaviors such as guarding or bracing), which can increase pain over time.^13–15,37^

Together, these findings suggest shared mechanisms that may underlie comorbidity. In this study, we used neural and behavioral reward-related variables, along with psychosocial determinants, to identify patterns associated with the comorbidity between pain and the severity of depressive or anxiety symptoms in young adults from the general population. Finally, we compared these signatures to better understand their respective contributions.

## 2. Methods

### 2.1. Dataset

The IMAGEN dataset (https://imagen-project.org/) is a longitudinal dataset with data from 8 European centers, which recruited typically developing 14-year-olds with no history of major mental, neurological, or medical conditions (eg, bipolar disorder, schizophrenia, neurodevelopmental disorders, premature birth, head trauma).^47^ Follow-ups were conducted after 2 years (follow-up 1) and 5 years (follow-up 2). This study used cross-sectional functional MRI (fMRI) and psychosocial data from 1,021 participants at follow-up 2 (54% women, mean age: 19.0 ± 0.7 years). After excluding missing data, 689 subjects remained for neuroimaging and 624 for psychosocial analysis, with 615 overlapping (58% women, mean age: 19.0 ± 0.7 years). For twin pairs, 1 twin was randomly excluded. Demographics and probability for the diagnosis of mental disorders are in Supplementary Tables S1 and S2 (available at http://links.lww.com/PR9/A352).

### 2.2. Functional MRI: data acquisition

In the IMAGEN project, scanner protocols were standardized and MRI acquisitions with identical phantoms were carried out at all sites to calibrate and harmonize data from different imaging hardware. Details on MRI acquisition parameters are summarized in Supplementary Tables S3 and S4 (available at http://links.lww.com/PR9/A352).

### 2.3. Functional MRI: experimental design

**Figure 1.**
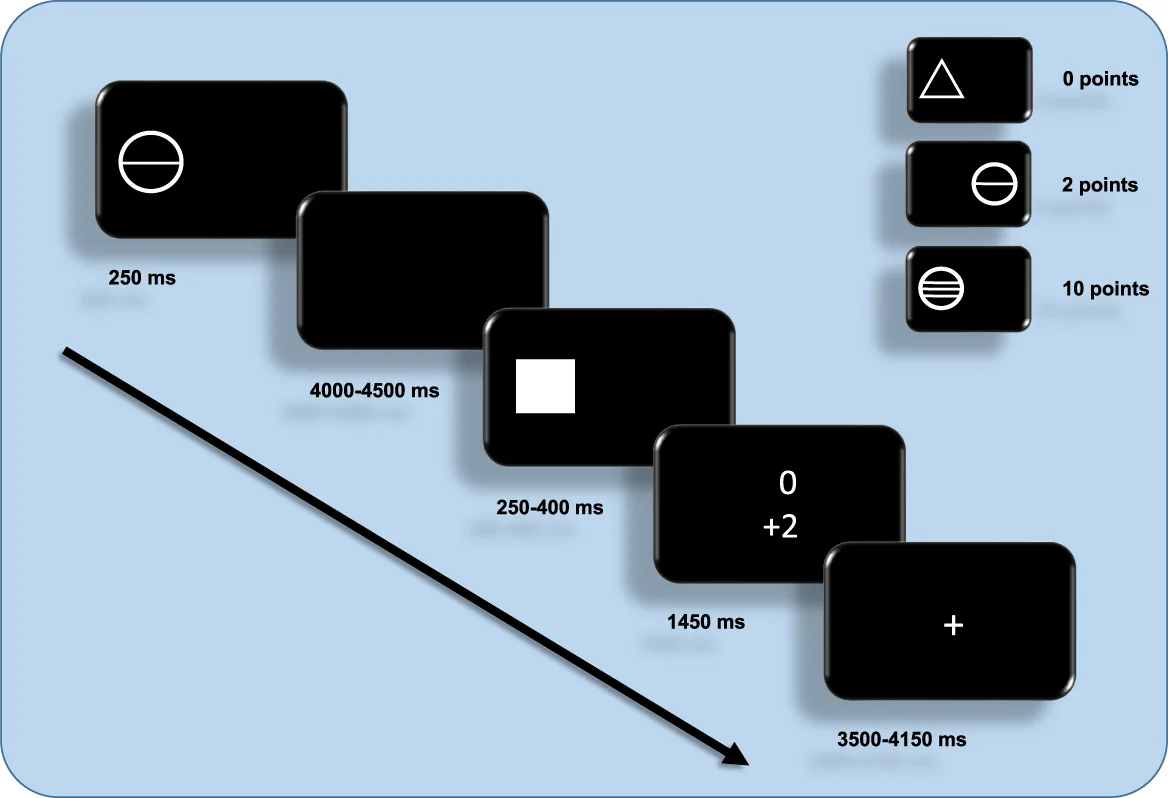
Monetary incentive delay task from the IMAGEN dataset. In each trial, participants were presented with an incentive cue, lasting 250 milliseconds, indicating the potential rewards associated with that trial. These cues took the form of symbols: a circle with 3 lines denoted a high reward (10 points), a circle with 1 line represented a lower reward (2 points), and a triangle signaled no reward (0 points). Following this cue presentation, a blank screen appeared for a variable duration of 4000 to 4500 milliseconds, followed by a target screen prompting participants to respond swiftly by pressing a button with either their left or right hand. The response window was dynamically adjusted to maintain a 66% success rate, making the task more challenging if performance exceeded this threshold and easier if below. Inadequate timing of responses, either premature or delayed, resulted in a failure to acquire points (reward miss). Conversely, precise timing within the adapted response interval led to successful point acquisition (reward hit). The accumulated and current trial points were displayed after the response window. An intertrial interval ranging from 3500 to 4150 milliseconds, featuring a fixation cross, followed.

The monetary incentive delay (MID) task employed in this study was designed to assess participants' reward learning as depicted in Figure 1.

### 2.4. Psychosocial data

We used standardized scores of pain, depression, and anxiety as outcome variables. Pain was assessed using the Children's Somatization Inventory.^35,51^ As pain outcome, we used the items strictly related to pain (1, 3, 5, 6, 16, 33, 34; corresponding to “Headache,” “Pain—heart or chest,” “Pain—lower back,” “Sore muscles,” “Pain—stomach,” “Pain—knees, elbows, joints,” “Pain—arms, legs”). The maximum pain intensity across these items was used as the outcome variable for pain. For depression, we employed the total score from the Adolescent Depression Rating Scale.^44^ For anxiety, we used the summary score derived from the first 12 items of the Anxiety Screening from the Diagnostic Interview for Mental Disorders (DIA-X) / Munich Composite International Diagnostic Interview (M-CIDI).^52^ Supplementary Figure S1 (available at http://links.lww.com/PR9/A352) shows the outcome distributions for the entire sample as well as for each center. Of note, 87% of our sample had pain symptoms, 89% had anxiety symptoms, and 44% had depressive symptoms, with 17% being above the cut-off for subthreshold depression.

As features in our psychosocial model, we included items from questionnaires and behavioral measures assessing decision-making abilities, experiences of trauma, use of alcohol and tobacco, substance use, bullism, monetary discounting, personality traits, instrumental learning, rumination, life events, and nonpainful somatic symptoms. Questionnaires and tests are listed in Supplementary Methods (see Data File S1 for all variables, available at http://links.lww.com/PR9/A352).

### 2.5. Functional MRI: data processing

#### 2.5.1. Preprocessing

We preprocessed the fMRI data using fMRIPrep 21.0.1,^11^ which is based on Nipype 1.6.1. The detailed steps are reported in Supplementary Methods (available at http://links.lww.com/PR9/A352).

#### 2.5.2. Data denoising and quality control

The output reports of fMRIPrep were visually inspected. Data displaying irreparable anomalies, such as missing brain segments, were excluded from subsequent analyses. Following this, we selected the best denoising procedure among 3 distinct approaches (see Supplementary Methods and Supplementary Fig. S2, available at http://links.lww.com/PR9/A352).

#### 2.5.3. First-level modelling: monetary incentive delay task

First-level analyses were conducted using the FMRI Expert Analysis Tool (FEAT) of the FMRIB Software Library (FSL) 6.0. The general linear model included the relevant predictors: conditions of interest (high reward, low reward, no reward), response of the subject (hit or miss), and phase (anticipation or feedback). These factors produced 12 contrasts vs baseline (eg, *Feedback_Hit_HighReward*). In addition, we considered pairwise reward contrasts (*HighReward* vs *NoReward*, *LowReward* vs *NoReward*) and aggregate contrasts combining multiple conditions to examine mechanisms independent of specific factors. For example, the contrast *Feedback_Hit* summed across reward levels, allowing assessment of effects that are independent of reward magnitude. Altogether, this resulted in 36 contrasts (Supplementary Table S5, available at http://links.lww.com/PR9/A352). The anticipation phase was modelled for 4 seconds after the appearance of the clue, and the feedback phase for 1.5 seconds after the target disappears. The canonical double-gamma Hemodynamic Response Function was implemented.

#### 2.5.4. Neurosynth “reward” mask

We selectively analyzed voxels within a “reward” mask (binarized, 12,969 voxels) obtained from neurosynth.org, spanning the midbrain, striatum, thalamus, anterior cingulate cortex (ACC), anterior insula, and vmPFC (Supplementary Fig. S3, available at http://links.lww.com/PR9/A352).

### 2.6. Psychosocial data processing

Participants with missing data were excluded, except for certain variables from the European School Survey Project on Alcohol and Drugs (ESPAD) and the Life Events Questionnaire (LEQ) (eg, age or frequency of experiences; see Data File S1 for counts). For these, missing values likely reflected the absence of the experience, so missing indicators were added. Predictors with zero variance were removed.

### 2.7. Multivariate statistical analysis

#### 2.7.1. Functional MRI

To uncover neural signatures associated with the comorbidities, we employed multitask learning (MTL), a machine learning technique that estimates multiple outcomes concurrently by exploiting a knowledge transfer between them. Multitask learning can identify signatures that are simultaneously predictive of multiple phenotypes.^54^

We separately built 1 MTL model for each of 36 blood-oxygen-level-dependent (BOLD) contrasts (Supplementary Table S5, available at http://links.lww.com/PR9/A352). Activity maps of all subjects were divided into development and hold-out samples, with 4 entire centers randomly assigned to each group. Within the development set, we used leave-one-center-out cross-validation (LOCOCV), assigning an entire center as the test fold and the remaining 3 centers as training in each iteration, with the 4 hold-out centers kept for final evaluation. Contrasts were standardized before dimensionality reduction using principal component analysis (PCA), using the “pca” function with default options in MATLAB R2018a (Mathworks, Natick, MA, RRID: SCR_001622). Standardization was performed within the training set only, and the same transformation (ie, centering and scaling parameters) was applied to the hold-out centers, to avoid information leakage across sites. The principal component (PC) scores for each subject were the features of the model, and the outcome measures were the standardized scores for pain, depression, and anxiety.

The MTL algorithm aimed at solving the following objective:

$$\min_{W}\sum_{i=1}^{t}\frac{1}{n_{i}}L\left(W_{i}|X_{i},Y_{i}\right)+\lambda {\left\|W\right\|}_{2,1},$$

where L(∘) is the least square loss function for regression, X_i_ and Y_i_ are the predictor matrix and outcome vector for task i∈{1,2,…,t}, respectively, n_i_ is the number of subjects of task i, and W is the coefficient matrix, with the i-th column, W_i_, consisting of the weights referring to task i. Knowledge transfer among tasks is achieved using the convex regularization term λ||W||_2,1_ based on the L_2,1_-norm, which extends the conventional lasso approach by allowing for joint feature selection across tasks.^6^ The parameter λ controls the strength of the regularization, where a higher value of λ encourages the model to select a smaller subset of features that optimize predictive performance across tasks. Thus, knowledge transfer primarily affects which features are selected, rather than the specific weight patterns for each task.

The parameters λ were selected using LOCOCV. Density-based weighting was applied during model training to address outcome imbalance. This technique assigns higher weights to subjects with less common outcomes and lower weights to those with more common outcomes. Specifically, the weight R_j_ assigned to a subject j, whose outcome has a relative frequency f_j_ in the training set, was computed as follows (note: these are distinct from the weights learned by the model):

$$R_{j}=C_{j}^{x}\times \left(n/\overset{n}{\underset{j=1}{\sum }}C_{j}^{x}\right),$$

where C_j_ = 1-f_j_ is the complement of the relative frequency f_j_ of a given outcome, x is an exponent regulating the strength of the reweighting procedure, and n is the number of subjects in the training set. Our primary analyses were based on x = 1, while additional analyses for x = 0 (no reweighting), x = 2, and x = 3 are presented in Supplementary Tables S6–S8 and Supplementary Figures S4–S6 (available at http://links.lww.com/PR9/A352).

The 3 best-performing models were selected during LOCOCV. For pain and the severity of anxiety symptoms, a clear performance gap separated the top 3 models from the rest, justifying their selection. Although no such distinct gap was observed for pain and the severity of depressive symptoms, we nonetheless selected the top 3 models to maintain consistency across analyses (Supplementary Fig. S7, available at http://links.lww.com/PR9/A352). Selection was based on the average mean squared error (MSE) between outcomes, requiring at least 1 selected PC. These models were tested on the hold-out centers using 10,000 1-sided permutation tests (significance threshold = 0.05).

Multitask learning was performed using Regularized Multi-Task Learning (RMTL) 0.9.9, an open-source R package for MTL, where the RMTL source code was additionally customized, implementing LOCOCV and density-based weighting.^6^

#### 2.7.2. Psychosocial models

The psychosocial analyses used the same method as the fMRI analyses, except that no PCA was performed; standardized item scores were used directly as features. In addition, we identified the most robust features by increasing the strictness of the MTL approach regarding variable selection (ie, increasing the value of the penalty term), and counting how many times a feature was selected. A variable is a robust indicator of comorbidity when (1) it is robustly selected during MTL when increasing the penalty term, and (2) the sign of its weight is the same for both outcomes. On the other hand, features with opposing weight signs for both outcomes are good at discriminating between the 2 conditions. The penalty term was increased, by steps of 0.01, from the value of λ calculated during LOCOCV to a maximum value of 0.99.

#### 2.7.3. Stacked ensemble model

To compare the predictive capabilities of neuroimaging and psychosocial indicators, we used significant models from both fMRI and psychosocial analyses. A unified model was created using the stacked ensemble technique, where the features of the second-order model consisted of estimates from the first-order models. The same MTL algorithm described above was applied. This composite model was trained on the hold-out sample, and its mean absolute error (MAE) was computed during LOCOCV. We used 10,000 1-sided permutation tests for assessment.

### 2.8. Preregistration

This study was preregistered on the Open Science Framework (https://doi.org/10.17605/OSF.IO/DPXW9); deviations from the original analysis are detailed in Supplementary Table S9 (available at http://links.lww.com/PR9/A352).

## 3. Results

### 3.1. A neural signature of the comorbidity between pain and the severity of depressive symptoms

**Figure 2.**
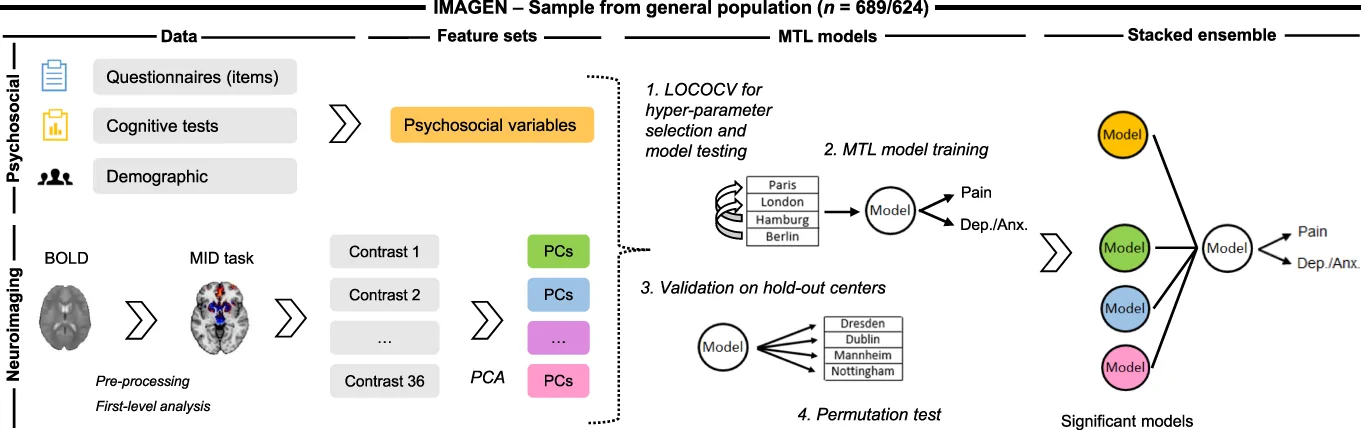
Flowchart of psychosocial and neuroimaging analyses. Preprocessed BOLD signals from the MID task, which involves monetary wins, were used to generate 36 contrasts (Supplementary Table S5, available at http://links.lww.com/PR9/A352). These contrasts underwent principal component analysis, producing principal components for MTL models. Psychosocial analyses used questionnaire items, cognitive tests, and demographics as MTL features. Models were created for pain and severity of depressive symptoms, and for pain and the severity of anxiety symptoms, ensuring shared feature selection using L_2,1_-norm in our MTL approach. Hyperparameters were selected using LOCOCV. The best models, selected based on mean squared errors from LOCOCV, were validated on hold-out centers through 10,000 1-sided permutation tests. Statistically significant models were combined using the stacked ensemble method, where estimates from first-level models became the features of a second-level model. Anx., anxiety; BOLD, blood-oxygen-level-dependent (signal); Dep., depression; LOCOCV, leave-one-center-out cross-validation; MID, monetary incentive delay; MTL, multitask learning; n, sample size in neuroimaging analysis/sample size in psychosocial analysis; PC, principal component; PCA, principal component analysis.

We used BOLD responses derived from an instrumental learning task (Fig. 1) to estimate current levels of pain and either depressive or anxiety symptoms, using an MTL approach as depicted in Figure 2. Given the weak Spearman correlations (ρ) between outcomes (Pain-Depression: ρ(687) = 0.28, *P* = 4.6 × 10^−14^; Pain-Anxiety: ρ(687) = 0.34, *P* = 2.3 × 10^−20^), MTL enabled us to target the specific component that is shared between these conditions.

The 3 best-performing models of the comorbidity between pain and the severity of depressive symptoms were derived from 3 BOLD contrasts based on reward feedback. All 3 models generalized to at least 3 of 4 hold-out centers, with validation being possible in some instances for only 1 of the 2 outcomes (*Feedback_Hit_HighReward*: MAE_pain_ = [0.57; 0.65; 0.59; 0.67], *P*_pain_ = [0.0028; 0.0016; 1.00; 1.00]; MAE_depression_ = [1.17; 1.69; 1.27; 1.75], *P*_depression_ = [0.0031; 1.00; 0.0030; 0.99]; *Feedback_Hit_LowReward*: MAE_pain_ = [0.57; 0.67; 0.59; 0.67], *P*_pain_ = [0.0026; 0.0023; 1.00; 1.00]; MAE_depression_ = [1.18; 1.66; 1.28; 1.76], *P*_depression_ = [0.0042; 1.00; 0.0042; 1.00]; *Feedback_HighReward*: MAE_pain_ = [0.57; 0.66; 0.59; 0.61], *P*_pain_ = [0.0001; 0.0002; 1.00; 1.00]; MAE_depression_ = [1.16; 1.71; 1.26; 1.78], *P*_depression_ = [0.0038; 0.0035; 0.0026; 0.0027]). In all 3 models, only the first PC was selected. The neural signatures of the 3 contrasts were reconstructed by projecting the model weights back into voxel space and were highly correlated (Pearson correlation: *r* = 0.98–0.99). This suggests that pain and the severity of depressive symptoms are encoded by a feedback-sensitive neural pattern common to all contrasts, whose activation strength matters more than reward magnitude per se. Owing to their nearly identical nature, only the model based on the BOLD responses to receiving a high reward is presented in detail in the Results section. However, all 3 models are described in detail in Supplementary Figure S8 (available at http://links.lww.com/PR9/A352). Each model contains 2 sets of weights, 1 for estimating pain symptoms and the other for estimating the severity of depressive symptoms. In all 3 significant models, the signatures for both pain and the severity of depressive symptoms were scaled versions of each other. This occurs because the model consistently selected a single PC (the first); if multiple components had been selected, the resulting voxelwise signatures would not, in general, be simple scaled versions. Thus, while the scaling itself reflects a property of the method, the substantive result is that the best joint prediction relies on a single shared spatial pattern. Additional analyses using alternative reweighting methods and outcome transformations (modifying skewness) confirmed that the first PC remained the most robust feature across approaches (Supplementary Figs. S4–S6, available at http://links.lww.com/PR9/A352). Moreover, the same pattern was found when using the sum scores of the pain items as the outcome measure for pain (Supplementary Fig. S9, available at http://links.lww.com/PR9/A352). Sum scores and maximum pain intensity were strongly correlated (ρ(687) = 0.84; *P* = 1.30 × 10^−184^). Taken together, these findings indicate that, based on neural circuits underlying reward processing, a single pattern estimated both pain and the severity of depressive symptoms.

**Figure 3.**
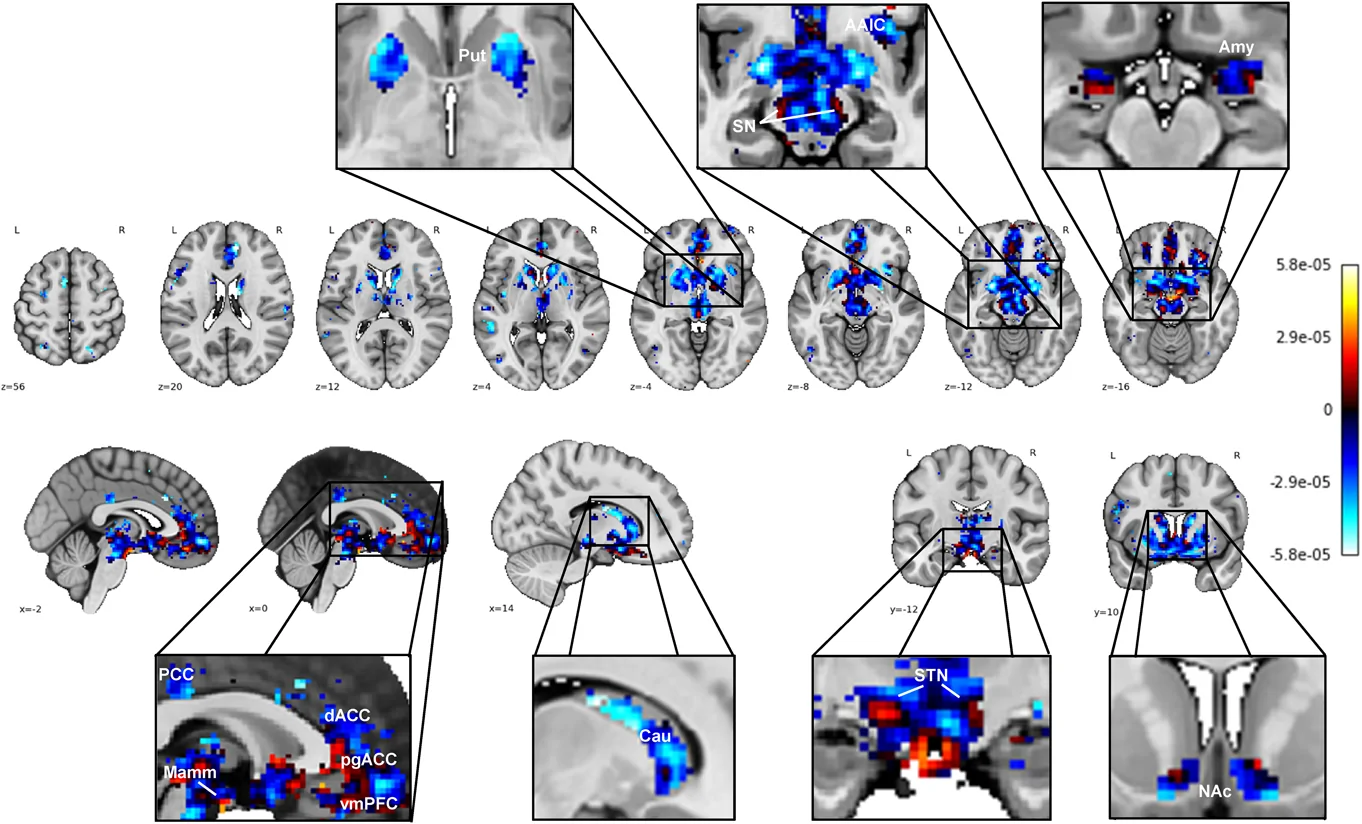
Neural signature of reward feedback associated with the comorbidity between pain and the severity of depressive symptoms. Neural signature significantly estimating both pain and the severity of depressive symptoms, built from the BOLD response to receiving a high reward during the MID task of the IMAGEN sample. Four magnified regions have been masked with their corresponding regions of interest (Put, Amy, Cau, NAc). A gray matter mask obtained from TemplateFlow^8^ was applied in this figure to improve anatomical distinction (for all weights in the unmasked signature, see Supplementary Fig. S8, available at http://links.lww.com/PR9/A352). Only the neural signature for the severity of depressive symptoms is exemplarily shown here. The color scale depicts the weights of the neural signature. AAIC, anterior agranular insula complex; Amy, amygdala; BOLD, blood-oxygen-level-dependent; Cau, nucleus caudatus; dACC, dorsal anterior cingulate cortex; Mamm, mammillary nucleus; MID, monetary incentive delay; NAc, nucleus accumbens; PCC, posterior cingulate cortex; pgACC, perigenual anterior cingulate cortex; Put, putamen; SN, substantia nigra; STN, subthalamic nucleus; vmPFC, ventromedial prefrontal cortex.

This pattern included negative weights in the caudate nucleus, putamen, posterior cingulate cortex (PCC), dorsal ACC, and right anterior insula and positive weights in the mammillary nucleus, substantia nigra (SN), and subthalamic nucleus (Fig. 3). The vmPFC, nucleus accumbens (NAc), perigenual ACC (pgACC), and amygdala showed mixed weights. Model predictions result from applying these weighted signatures to individual activation maps: while the activation map reflects task-related changes in the BOLD signal, the neural signature assigns weights to voxels according to their contribution to predicting the outcomes. The univariate group statistics (Supplementary Fig. S10, available at http://links.lww.com/PR9/A352) showed increased BOLD responses in voxels of the vmPFC, pgACC, and PCC during reward feedback; however, only the PCC had consistently negative weights, linking greater BOLD responses to lower symptom severity. Similarly, striatal regions with reduced BOLD responses also had negative weights, indicating that more negative reward responses are associated with higher pain and severity of depressive symptoms. Taken together, the neural signature and group statistics indicate directionally heterogeneous alterations in reward responsivity.

**Figure 4.**
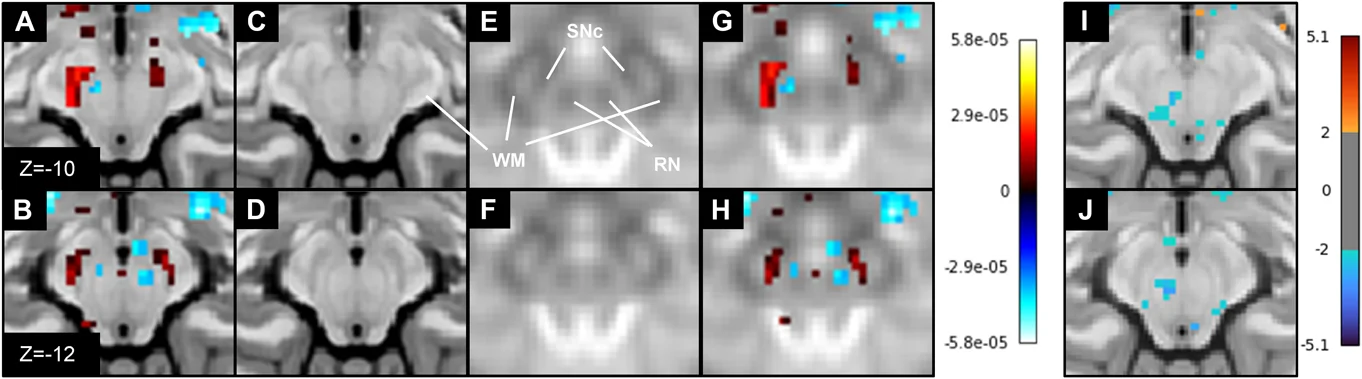
Substantia nigra contains high positive weights in the model for the comorbidity between pain and the severity of depressive symptoms during reward feedback. Neural signature in 2 adjacent slices at the level of the midbrain (MNI coordinates: Z = −10, Z = −12), projected onto both the anatomical MNI template and the average fMRI image of all the subjects, to show the overlap between the bilateral cluster of high positive weights and the substantia nigra. (A, B) Neural signature projected onto the MNI template; (C, D) MNI template; (E, F) fMRI average image; (G, H) neural signature projected onto the fMRI average image. Only the 10% highest and 10% lowest weights of the neural signature are shown. The color scale depicts positive and negative weights of the signature. (I, J) Group statistics of the contrast *Feedback_Hit_HighReward* projected onto the MNI template, with a significance level of *P* = 0.05 and a threshold Z = 1.96. Color scale indicates the z-values. fMRI, functional MRI; MNI, Montreal Neurological Institute; RN, red nucleus; SNc, substantia nigra pars compacta; WM, white matter.

In Figure 4, we projected the neural signature onto the average fMRI image, which shows a good alignment between the SN and the positive weights of the model. However, the SN was not significant in the group statistics (Fig. 4I–J), preventing us from determining the direction of its contribution to outcome estimation.

The 3 best-performing models for the comorbidity between pain and the severity of anxiety symptoms were not statistically significant in hold-out centers, as detailed in Supplementary Table S10 (see Data Files S2 and S3 for MSEs across all contrasts, available at http://links.lww.com/PR9/A352).

### 3.2. Psychosocial signatures of the comorbidity between pain and the severity of affective symptoms

**Figure 5.**
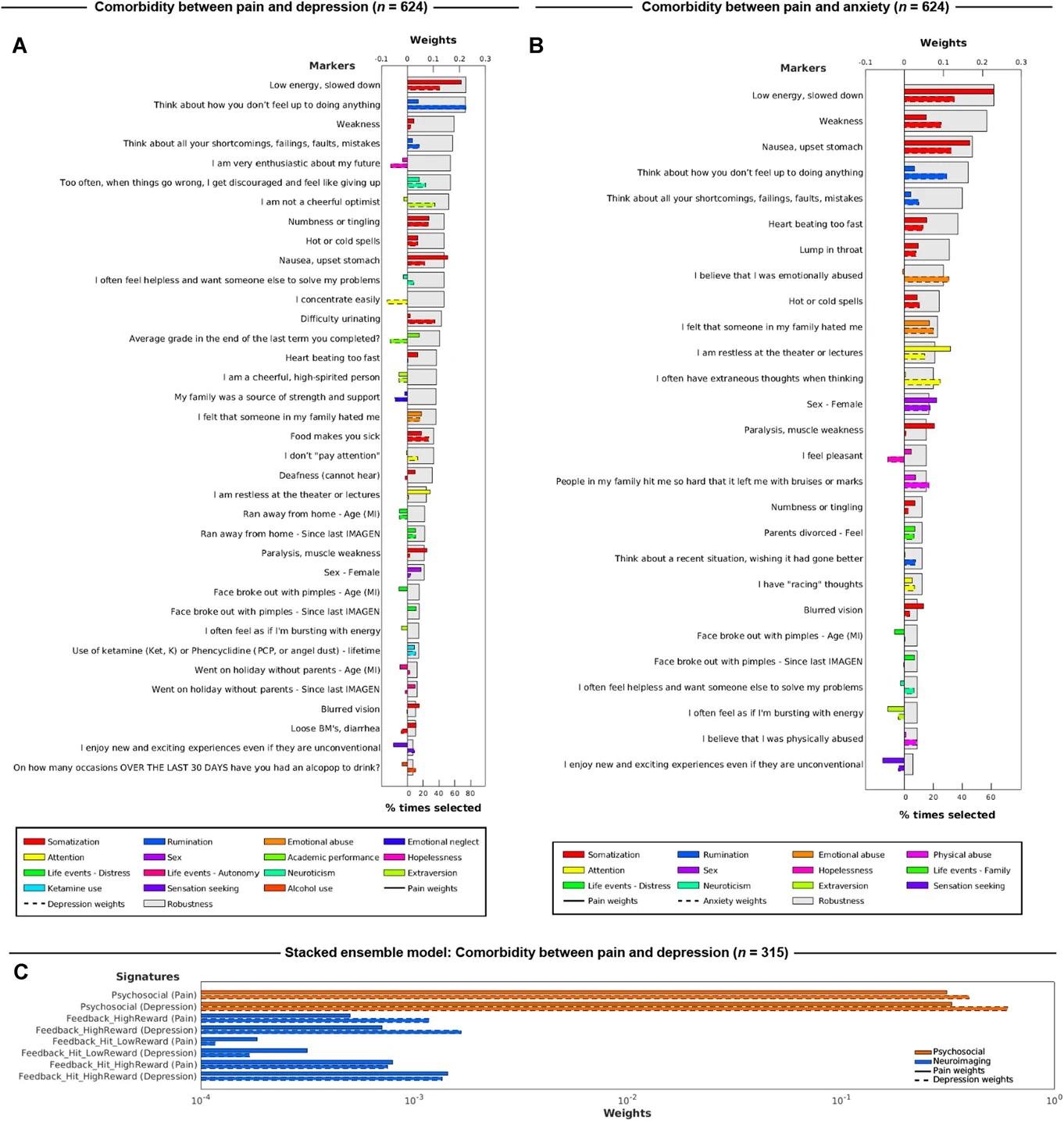
Psychosocial markers of the 2 types of comorbidity and a stacked ensemble model combining neural and psychosocial signatures. (A, B) Psychosocial markers of the comorbidity between pain and the severity of depressive symptoms, or pain and the severity of anxiety symptoms, respectively. (C) Stacked ensemble model of the comorbidity between pain and the severity of depressive symptoms, combining the significant neural and psychosocial signatures. The presented features in (A) and (B) meet 2 criteria: selected at least 10% of the time compared with the most robust feature, and have at least 1 weight that is a minimum of 10% of the highest weight. Complete lists of selected features and weights are in Data Files S4 and S5 (available at http://links.lww.com/PR9/A352). Gray bars indicate feature robustness, and colored bars show model weights, with continuous lines for pain and dashed lines for depression or anxiety. The upper *x*-axis shows weights, and the lower *x*-axis shows feature robustness. Labels and colors denote constructs and categories specified in the legend. In (C), significant psychosocial and neural signatures of the comorbidity between pain and the severity of depressive symptoms are shown as features of the stacked ensemble model. Continuous line bars represent weights for pain, while dashed bars represent weights for depression. The *x*-axis is logarithmically scaled, with colors differentiating psychosocial and neural signatures. MI, missing indicator.

We used items from questionnaires and behavioral measures to estimate pain and the severity of comorbid depressive/anxiety symptoms, which were only weakly correlated (Pain-Depression: ρ(622) = 0.28, *P* = 2.5 × 10^−12^; Pain-Anxiety: ρ(622) = 0.34, *P* = 6.1 × 10^−18^). The MTL models for both types of comorbidity (Fig. 5A and B) were statistically significant in hold-out centers (Pain-Depression: MAE_pain_ = [0.45; 0.56; 0.55; 0.64], *P*_pain_ = [<0.0001; 0.0010; 0.0048; 0.096]; MAE_depression_ = [0.86; 1.10; 1.01; 1.35], *P*_depression_ = <0.0001 [all]; Pain-Anxiety: MAE_pain_ = [0.46; 0.58; 0.56; 0.62], *P*_pain_ = [<0.0001; 0.0063; 0.016; 0.016]; MAE_anxiety_ = [3.58; 3.61; 4.18; 4.31], *P*_anxiety_ = <0.0001 [all]).

The primary indicators for the comorbidity between pain and the severity of depressive symptoms were items associated with nonpainful somatic symptoms, rumination, neuroticism, extraversion, and feelings of hopelessness. Similarly, the primary markers for the comorbidity between pain and the severity of anxiety symptoms included items linked to nonpainful somatic symptoms, rumination, emotional abuse, lack of attention, and female sex.

To illustrate the difference between multitask and single-task learning models, we separately trained psychosocial models for pain and for the severity of depressive symptoms (Supplementary Fig. S11, available at http://links.lww.com/PR9/A352). As described in the Methods section, knowledge transfer across tasks in MTL is primarily reflected in the features selected by the model. Consequently, while the pattern of weights may be similar between multitask and single-task models, the sets of selected features and their robustness differ.

### 3.3. The predictive power of psychosocial compared with neural signatures

We trained a composite model comprising the statistically significant neural signatures and the psychosocial signature of the comorbidity between pain and the severity of depressive symptoms (MAE_pain_: 0.62, *P*_pain_ < 0.0001, MAE_depression_: 1.22, *P*_depression_ < 0.0001; Fig. 5C). The weights assigned to this second-level model indicated that the influence of the psychosocial signature on outcome estimation significantly outweighed that of the neural signatures, by orders of magnitude (10^2^-10^3^). To understand whether the neural signatures significantly improve the estimation of pain and depressive symptom severity, we compared the absolute errors of the stacked ensemble model comprising the psychosocial signature with or without the neural signatures. Including the neural signatures slightly but significantly improved the model performance (Wilcoxon signed-rank test: ΔMAE_pain_ = −5.36 × 10^−4^, V = 17,566, *P* = 6.06 × 10^−6^, n = 315 pairs; ΔMAE_depression_ = −0.024, V = 15,196, *P* = 2.11 × 10^−9^, n = 315 pairs).

## 4. Discussion

This study aimed to identify neural and psychosocial indicators of the comorbidity between pain and the severity of affective symptoms in young adults from the general population. Using reward-related neural signals and psychosocial data, we uncovered distinct and shared patterns associated with pain and the severity of depressive/anxiety symptoms.

### 4.1. A neural signature of the comorbidity between pain and the severity of depressive symptoms

Based on BOLD responses to reward feedback, we identified a neural signature of the comorbidity between pain and the severity of depressive symptoms, involving key regions linked to reward learning and instrumental behavior, such as the ventral and dorsal striatum, SN, vmPFC, and ACC.^20,21,24,28,45,46^

The dorsal striatum is key to forming instrumental associations between actions and rewards,^20,24,46^ while the ventral striatum is involved in motivational processes such as behavioral activation, sustained task engagement, and exertion of effort during instrumental behavior.^46^ The vmPFC is important for value-based decision making, encoding evolving outcome values.^21^ Specifically, the anterior/pregenual vmPFC represents rewards and costs on a common scale.^28^ The pgACC integrates information on the availability of rewards coming from the medial OFC and the corresponding actions required to attain them.^45^

Deficits in instrumental learning may underlie both depressive and pain symptoms,^2,13–15,21,37,41,42^ contributing to impaired goal-directed behavior, which is a common feature of both conditions.^12,46^ Our neural signature aligns with imaging findings showing that reward signals in the striatum and OFC of depressed individuals are altered during anticipation and feedback phases, while in chronic back pain, vmPFC responses to reward feedback are reduced.^1,27,30,42^ In addition, the transition from subacute to chronic pain is predicted by increased reward feedback encoding in the NAc.^30^

Our results point to the involvement of the dorsal striatum and SN in the comorbidity between pain and the severity of depressive symptoms. As in depression, altered dorsal striatum signals may impair learning of action-reward associations.^42^ These instrumental associations require dopamine (DA) release from the SN to the dorsal striatum.^20,24,46^ However, both chronic pain and depression are characterized by a hypoactive dopaminergic state.^42,48^ Preclinical evidence shows that the dopaminergic system contributes to the development of their comorbidity.^33^ In murine models of neuropathic pain, reduced activity of ventral tegmental area DA neurons projecting to the NAc underlies anhedonia-like behavior, while activating this pathway restores motivation.^33^ This reduction is in part due to a feedback loop with the ACC.^49^ Despite lower baseline activity, ventral tegmental area DA neurons show enhanced phasic reward responses.^33^ If similar increases occur in the SN, they could explain the positive SN weights in our neural signature, though this should be interpreted with caution given the lack of group-level effects.

We did not find significant neural signatures of comorbid pain and anxiety symptom severity, possibly due to limitations of the MID task in capturing relevant learning components for anxiety. Notably, anxiety is associated with increased sensitivity to punishments.^7,25^ The MID task used in the IMAGEN study did not involve explicit punishments, which may be essential for revealing the components of instrumental learning associated with anxiety.

### 4.2. Psychosocial signatures of the comorbidity between pain and the severity of affective symptoms

Psychosocial markers, particularly nonpainful somatic symptoms and rumination, were strong predictors for both types of comorbidity. This is consistent with prior literature on overlapping symptom clusters, such as the SPADE pentad (Sleep disturbance, Pain, Anxiety, Depression, and low Energy/fatigue),^9,26^ while extending that work by demonstrating the relative robustness of psychosocial indicators for each specific comorbidity. Importantly, we also observed differential patterns in predictor strength. For instance, emotional neglect was robustly associated with depressive comorbidity, while physical abuse was more predictive of anxiety comorbidity.

### 4.3. The predictive power of psychosocial compared with neural signatures

Neural signatures of reward feedback improved the prediction of a psychosocial signature for the comorbidity between pain and the severity of depressive symptoms. The stronger influence of psychosocial factors may reflect their broader scope compared with the more limited neural measures focused on a specific type of reward learning. This aligns with prior multimodal studies, where psychosocial data outperformed neuroimaging but both provided complementary information, with the combined models performing best.^22,36^

### 4.4. Limitations

The IMAGEN dataset is skewed toward lower depression, pain, and anxiety scores (Supplementary Fig. S1, available at http://links.lww.com/PR9/A352). Nevertheless, pain and affective symptoms were prevalent, highlighting the importance of investigating these comorbidities in young adults. Although symptom severity may be lower than in older populations, epidemiological studies report chronic pain in 5% to 30% of young adults, depending on definitions and populations.^5,38^ In addition, skewed outcomes can hinder predictive modeling due to limited variability. To address this, we applied density-based weighting, but since low clinical scores predominated, our signatures may reflect the comorbidity of mild symptoms, limiting applicability to severe or chronic cases. By focusing our neuroimaging analysis on reward learning, we may have missed other neural domains relevant to the comorbidity. Moreover, depression, pain, and anxiety are heterogeneous conditions with varying symptom spectra, which different scales might capture differently.^16^ Consequently, our findings should be interpreted in the context of the questionnaires used in this study. In addition, we did not explicitly model or correct for site effects in our analyses. However, the IMAGEN project implemented several measures to minimize intersite variability (see Methods), and our use of LOCOCV and testing on hold-out centers provides a strong safeguard against overfitting to site-specific artifacts. Finally, as our sample consists of young adults who may still be undergoing developmental changes, the generalizability of our signatures to middle-aged or older individuals may be limited.

## 5. Conclusions

We identified neural and psychosocial signatures of the comorbidity between pain and the severity of affective symptoms in young adults from the general population. Neural responses during reward feedback were associated with the comorbidity between pain and the severity of depressive symptoms and improved prediction when added to psychosocial indicators. Psychosocial variables, particularly nonpainful somatic symptoms and rumination, were robust predictors across both types of comorbidity. These findings may inform early detection and intervention strategies.

## Disclosures

T. Banaschewski served in an advisory or consultancy role for Infectopharm, Medice, Neurim Pharmaceuticals, Oberberg GmbH, Takeda, Eye Level, and AGB pharma; he received conference support or speaker fees from Janssen-Cilag, Medice, AGB Pharma, and Takeda; he has been involved in clinical trials conducted by Shire & Viforpharma; and he received royalties from Hogrefe, Kohlhammer, CIP Medien, Oxford University Press. P. Gowland received funding from Wellcome Leap, EPSRC, Nestle, BBSRC, Rosetrees, and Stinygate Trust, and he received support to attend meetings as organizer or speaker in some capacity from the International Society for Magnetic Resonance in Medicine. L. Poustka served in an advisory or consultancy role for Roche and Viforpharm and received speaker fees from Shire; she also received royalties from Hogrefe, Kohlhammer, and Schattauer. E. Schwarz received speaker fees from bfd buchholz-fachinformationsdienst GmbH. This study is unrelated to the above grants and relationships. The other authors have no conflicts of interest to declare.

## Supplemental digital content

Supplemental digital content associated with this article can be found online at http://links.lww.com/PR9/A352.

## Supplementary Material

## References

[1] Admon R Kaiser RH Dillon DG Beltzer M Goer F Olson DP Vitaliano G Pizzagalli DA. Dopaminergic enhancement of striatal response to reward in major depression. Am J Psychiatry 2017;174:378–86. 10.1176/appi.ajp.2016.16010111 PMC5378658 27771973

[2] Admon R Pizzagalli DA. Dysfunctional reward processing in depression. Curr Opin Psychol 2015;4:114–8. 10.1016/j.copsyc.2014.12.011 PMC4525714 26258159

[3] Bair MJ Robinson RL Katon W Kroenke K. Depression and pain comorbidity: a literature review. Arch Intern Med 2003;163:2433–45. 10.1001/archinte.163.20.2433 14609780

[4] Bondesson E Larrosa Pardo F Stigmar K Ringqvist Å Petersson IF Jöud A Schelin MEC. Comorbidity between pain and mental illness: evidence of a bidirectional relationship. Eur J Pain 2018;22:1304–11. 10.1002/ejp.1218 29577509

[5] Brown D Schenk S Genent D Zernikow B Wager J. A scoping review of chronic pain in emerging adults. Pain Rep 2021;6:e920. 10.1097/PR9.0000000000000920 PMC8546842 34712883

[6] Cao H Zhou J Schwarz E. RMTL: an R library for multi-task learning. Bioinformatics 2019;35:1797–8. 10.1093/bioinformatics/bty831 30256897

[7] Cavanagh JF Bismark AW Frank MJ Allen JJB. Multiple dissociations between comorbid depression and anxiety on reward and punishment processing: evidence from computationally informed EEG. Comput Psychiatr 2019;3:1–17. 10.1162/cpsy_a_00024 PMC6515849 31149639

[8] Ciric R Thompson WH Lorenz R Goncalves M MacNicol EE Markiewicz CJ Halchenko YO Ghosh SS Gorgolewski KJ Poldrack RA Esteban O. TemplateFlow: FAIR-sharing of multi-scale, multi-species brain models. Nat Methods 2022;19:1568–71. 10.1038/s41592-022-01681-2 PMC9718663 36456786

[9] Davis LL Kroenke K Monahan P Kean J Stump TE. The SPADE symptom cluster in primary care patients with chronic pain. Clin J Pain 2016;32:388–93. 10.1097/AJP.0000000000000286 PMC4761335 26295379

[10] Edwards RR Dworkin RH Sullivan MD Turk DC Wasan AD. The role of psychosocial processes in the development and maintenance of chronic pain. J Pain 2016;17:T70–92. 10.1016/j.jpain.2016.01.001 PMC5012303 27586832

[11] Esteban O Markiewicz CJ Blair RW Moodie CA Isik AI Erramuzpe A Kent JD Goncalves M DuPre E Snyder M Oya H Ghosh SS Wright J Durnez J Poldrack RA Gorgolewski KJ. fMRIPrep: a robust preprocessing pipeline for functional MRI. Nat Methods 2019;16:111–6. 10.1038/s41592-018-0235-4 PMC6319393 30532080

[12] Fisher E Palermo TM. Goal pursuit in youth with chronic pain. Children (Basel) 2016;3:36. 10.3390/children3040036 PMC5184811 27879686

[13] Flor H. New developments in the understanding and management of persistent pain. Curr Opin Psychiatry 2012;25:109–13. 10.1097/YCO.0b013e3283503510 22227632

[14] Flor H Knost B Birbaumer N. The role of operant conditioning in chronic pain: an experimental investigation. PAIN 2002;95:111–8. 10.1016/s0304-3959(01)00385-2 11790473

[15] Fordyce WE. Behavioral methods for chronic pain and illness. St. Louis: Mosby, 1976.

[16] Fried EI. The 52 symptoms of major depression: lack of content overlap among seven common depression scales. J Affect Disord 2017;208:191–7. 10.1016/j.jad.2016.10.019 27792962

[17] Geugies H Mocking RJT Figueroa CA Groot PFC Marsman JBC Servaas MN Steele JD Schene AH Ruhé HG. Impaired reward-related learning signals in remitted unmedicated patients with recurrent depression. Brain 2019;142:2510–22. 10.1093/brain/awz167 PMC6734943 31280309

[18] Halahakoon DC Kieslich K O'Driscoll C Nair A Lewis G Roiser JP. Reward-processing behavior in depressed participants relative to healthy volunteers: a systematic review and meta-analysis. JAMA Psychiatry 2020;77:1286–95. 10.1001/jamapsychiatry.2020.2139 PMC7391183 32725180

[19] Hess LE Haimovici A Muñoz MA Montoya P. Beyond pain: modeling decision-making deficits in chronic pain. Front Behav Neurosci 2014;8:263. 10.3389/fnbeh.2014.00263 PMC4117932 25136301

[20] Hikosaka O Kim HF Amita H Yasuda M Isoda M Tachibana Y Yoshida A. Direct and indirect pathways for choosing objects and actions. Eur J Neurosci 2019;49:637–45. 10.1111/ejn.13876 PMC6107440 29473660

[21] Hiser J Koenigs M. The multifaceted role of the ventromedial prefrontal cortex in emotion, decision making, social cognition, and psychopathology. Biol Psychiatry 2018;83:638–47. 10.1016/j.biopsych.2017.10.030 PMC5862740 29275839

[22] Ho TC Shah R Mishra J May AC Tapert SF. Multi-level predictors of depression symptoms in the Adolescent Brain Cognitive Development (ABCD) study. J Child Psychol Psychiatry 2022;63:1523–33. 10.1111/jcpp.13608 PMC9489813 35307818

[23] Holth HS Werpen HK Zwart JA Hagen K. Physical inactivity is associated with chronic musculoskeletal complaints 11 years later: results from the Nord-Trøndelag Health Study. BMC Musculoskelet Disord 2008;9:159. 10.1186/1471-2474-9-159 PMC2606680 19046448

[24] Jeong H Taylor A Floeder JR Lohmann M Mihalas S Wu B Zhou M Burke DA Namboodiri VMK. Mesolimbic dopamine release conveys causal associations. Science 2022;378:eabq6740. 10.1126/science.abq6740 PMC9910357 36480599

[25] Katz BA Matanky K Aviram G Yovel I. Reinforcement sensitivity, depression and anxiety: a meta-analysis and meta-analytic structural equation model. Clin Psychol Rev 2020;77:101842. 10.1016/j.cpr.2020.101842 32179341

[26] Kroenke K Talib TL Stump TE Kean, J Haggstrom DA DeChant P Lake KR Stout M Monahan PO. Incorporating PROMIS symptom measures into primary care practice-a randomized clinical trial. J Gen Intern Med 2018;33:1245–52. 10.1007/s11606-018-4391-0 PMC6082211 29623512

[27] Kumar P Goer F Murray L Dillon DG Beltzer ML Cohen AL Brooks NH Pizzagalli DA. Impaired reward prediction error encoding and striatal-midbrain connectivity in depression. Neuropsychopharmacology 2018;43:1581–8. 10.1038/s41386-018-0032-x PMC5983542 29540863

[28] Levy DJ Glimcher PW. The root of all value: a neural common currency for choice. Curr Opin Neurobiol 2012;22:1027–38. 10.1016/j.conb.2012.06.001 PMC4093837 22766486

[29] Lindell M Grimby-Ekman A. Stress, non-restorative sleep, and physical inactivity as risk factors for chronic pain in young adults: a cohort study. PLoS One 2022;17:e0262601. 10.1371/journal.pone.0262601 PMC8782303 35061825

[30] Löffler M Levine SM Usai K Desch S Kandić M Nees F Flor H. Corticostriatal circuits in the transition to chronic back pain: the predictive role of reward learning. Cell Rep Med 2022;3:100677. 10.1016/j.xcrm.2022.100677 PMC9381385 35798001

[31] Macedo BBD von Werne Baes C Menezes IC Juruena MF. Child abuse and neglect as risk factors for comorbidity between depression and chronic pain in adulthood. J Nerv Ment Dis 2019;207:538–45. 10.1097/NMD.0000000000001031 31192794

[32] Marbach JJ Richlin DM Lipton JA. Illness behavior, depression and anhedonia in myofascial face and back pain patients. Psychother Psychosom 1983;39:47–54. 10.1159/000287720 6220421

[33] Markovic T Pedersen CE Massaly N Vachez YM Ruyle B Murphy CA Abiraman K Shin JH Garcia JJ Yoon HJ Alvarez VA Bruchas MR Creed MC Morón JA. Pain induces adaptations in ventral tegmental area dopamine neurons to drive anhedonia-like behavior. Nat Neurosci 2021;24:1601–13. 10.1038/s41593-021-00924-3 PMC8556343 34663957

[34] McLaughlin KA Nolen-Hoeksema S. Rumination as a transdiagnostic factor in depression and anxiety. Behav Res Ther 2011;49:186–93. 10.1016/j.brat.2010.12.006 PMC3042543 21238951

[35] Meesters C Muris P Ghys A Reumerman T Rooijmans M. The Children's Somatization Inventory: further evidence for its reliability and validity in a pediatric and a community sample of Dutch children and adolescents. J Pediatr Psychol 2003;28:413–22. 10.1093/jpepsy/jsg031 12904453

[36] Mellem MS Liu Y Gonzalez H Kollada M Martin WJ Ahammad P. Machine learning models identify multimodal measurements highly predictive of transdiagnostic symptom severity for mood, anhedonia, and anxiety. Biol Psychiatry Cogn Neurosci Neuroimaging 2020;5:56–67. 10.1016/j.bpsc.2019.07.007 31543457

[37] Meulders A Vansteenwegen D Vlaeyen JWS. The acquisition of fear of movement-related pain and associative learning: a novel pain-relevant human fear conditioning paradigm. PAIN 2011;152:2460–9. 10.1016/j.pain.2011.05.015 21723664

[38] Murray CB de la Vega R Murphy LK Kashikar-Zuck S Palermo TM. The prevalence of chronic pain in young adults: a systematic review and meta-analysis. PAIN 2022;163:e972–84. 10.1097/j.pain.0000000000002541 34817439

[39] Nees F Ruttorf M Fuchs X Rance M Beyer N. Brain-behaviour correlates of habitual motivation in chronic back pain. Sci Rep 2020;10:11090. 10.1038/s41598-020-67386-8 PMC7338353 32632166

[40] Nees F Usai K Löffler M Flor H. The evaluation and brain representation of pleasant touch in chronic and subacute back pain. Neurobiol Pain 2019;5:100025. 10.1016/j.ynpai.2018.10.002 PMC6550103 31194113

[41] Pike AC Robinson OJ. Reinforcement learning in patients with mood and anxiety disorders vs control individuals: a systematic review and meta-analysis. JAMA Psychiatry 2022;79:313–22. 10.1001/jamapsychiatry.2022.0051 PMC8892374 35234834

[42] Pizzagalli DA. Depression, stress, and anhedonia: toward a synthesis and integrated model. Annu Rev Clin Psychol 2014;10:393–423. 10.1146/annurev-clinpsy-050212-185606 PMC3972338 24471371

[43] Radat F Margot-Duclot A Attal N. Psychiatric co-morbidities in patients with chronic peripheral neuropathic pain: a multicentre cohort study. Eur J Pain 2013;17:1547–57. 10.1002/j.1532-2149.2013.00334.x 23720357

[44] Revah-Levy A Birmaher B Gasquet I Falissard B. The Adolescent Depression Rating Scale (ADRS): a validation study. BMC Psychiatry 2007;7:2. 10.1186/1471-244X-7-2 PMC1785378 17222346

[45] Rolls ET. The cingulate cortex and limbic systems for emotion, action, and memory. Brain Struct Funct 2019;224:3001–18. 10.1007/s00429-019-01945-2 PMC6875144 31451898

[46] Salamone JD Correa M. The mysterious motivational functions of mesolimbic dopamine. Neuron 2012;76:470–85. 10.1016/j.neuron.2012.10.021 PMC4450094 23141060

[47] Schumann G Loth E Banaschewski T Barbot A Barker G Büchel C Conrod PJ Dalley JW Flor H Gallinat J Garavan H Heinz A Itterman B Lathrop M Mallik C Mann K Martinot JL Paus T Poline J-B Robbins TW Rietschel M Reed L Smolka M Spanagel R Speiser C Stephens DN Ströhle A Struve M; IMAGEN consortium. The IMAGEN study: reinforcement-related behaviour in normal brain function and psychopathology. Mol Psychiatry 2010;15:1128–39. 10.1038/mp.2010.4 21102431

[48] Serafini RA Pryce KD Zachariou V. The mesolimbic dopamine system in chronic pain and associated affective comorbidities. Biol Psychiatry 2020;87:64–73. 10.1016/j.biopsych.2019.10.018 PMC6954000 31806085

[49] Song Q Wei A Xu H Gu Y Jiang Y Dong N Zheng C Wang Q Gao M Sun S Duan X Chen Y Wang B Huo J Yao J Wu H Li H Wu X Jing Z Liu X Yang Y Hu S Zhao A Wang H Cheng X Qin Y Qu Q Chen T Zhou Z Chai Z Kang X Wei F Wang C. An ACC–VTA–ACC positive-feedback loop mediates the persistence of neuropathic pain and emotional consequences. Nat Neurosci 2024;27:272–85. 10.1038/s41593-023-01519-w 38172439

[50] Verdejo-García A López-Torrecillas F Calandre EP Delgado-Rodríguez A Bechara A. Executive function and decision-making in women with fibromyalgia. Arch Clin Neuropsychol 2009;24:113–22. 10.1093/arclin/acp014 19395361

[51] Walker LS Beck JE Garber J Lambert W. Children's Somatization Inventory: psychometric properties of the revised form (CSI-24). J Pediatr Psychol 2009;34:430–40. 10.1093/jpepsy/jsn093 PMC2722132 18782857

[52] Wittchen HU Pfister H. DIA-X-interviews: manual für screening-verfahren und interview; Interviewheft. Frankfurt: Swets & Zeitlinger; 1997.

[53] Yamamori Y Robinson OJ Roiser JP. Approach-avoidance reinforcement learning as a translational and computational model of anxiety-related avoidance. Elife 2023;12:RP87720. 10.7554/eLife.87720 PMC10645421 37963085

[54] Zhou J Liu J Narayan VA Ye J; Alzheimer's Disease Neuroimaging Initiative. Modeling disease progression via multi-task learning. Neuroimage 2013;78:233–48. 10.1016/j.neuroimage.2013.03.073 23583359

