# Supplementary material for: Neural and psychosocial signatures of the comorbidity between pain and affective symptoms
Source: Pain Rep. 2025 Dec 12;11(1):e1353. doi: 10.1097/PR9.0000000000001353 (PMC12705059; doi:10.1097/PR9.0000000000001353)
Supplement: Supplementary file 1 [file painreports-11-e1353-s001.pdf]

Supplementary Information

- **Supplementary Figures**
- **Supplementary Tables**
- **Supplementary Methods**
- **Data Files S1-S5**

Supplementary Figures

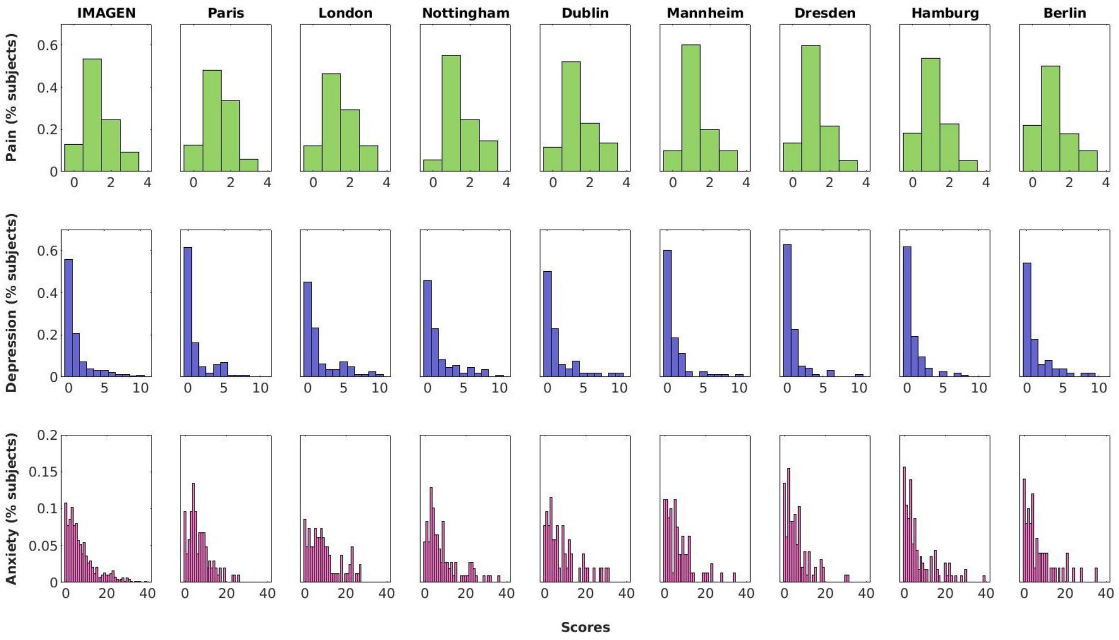

Supplementary Figure S1. Distribution of the outcomes for the whole dataset and across sites in the IMAGEN sample. Data based on the sample used in the neuroimaging analysis (*n* = 689).

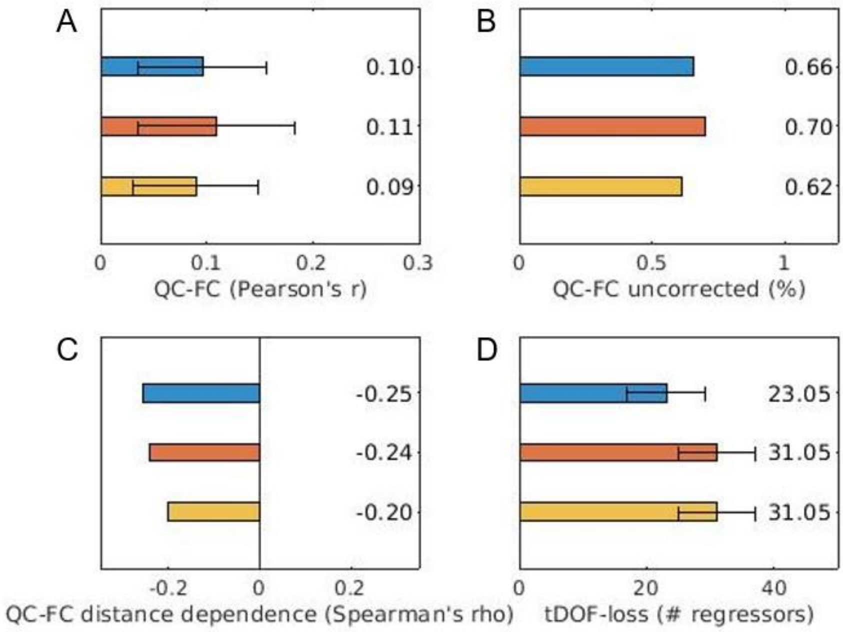

Supplementary Figure S2. Quality metrics used to assess the efficacy of different denoising strategies. Four quality metrics were used to compare three denoising strategies: (**A**) median absolute QC-FC correlation, and (**B**) the proportion of edges where the QC-FC correlation was significant. The QC-FC correlation is the inter-subject correlation between framewise displacement (FD) and functional connectivity between pairs of regions following noise correction. It quantifies the efficacy of each method in reducing motion-related variability. (**C**) QC-FC distance dependence, which is the correlation between the QC-FC metric and the Euclidian distance between brain regions. It measures how motion affects short-range connectivity more than medium- and long-range connectivity. (**D**) tDOF-loss, which refers to the reduction in temporal degrees of freedom (tDOF) that occurs when using more nuisance regressors to model noise in fMRI time series data. Greater tDOF loss leads to reduced statistical power, which may artificially elevate functional connectivity measures. The three denoising strategies were: 1) ICA-AROMA: we used (smoothed) fMRIPrep outputs denoised with ICA-AROMA; 2) ICA-AROMA + 8 Phys (aggressive): GLM was applied to the (smoothed) ICA-AROMA denoised outputs to regress out 8 physiological confounds: WM and CSF mean signals, their derivatives, squares, and squares of derivatives; 3) ICA-AROMA + 8 Phys (non-aggressive): for this approach, we denoised (non-smoothed) pre-processed data by adding the 8 physiological confounds to independent components, encompassing both signal and noise components. ICA-AROMA + 8 Phys (non-aggressive) was superior in 3 metrics out of 4. Blue = ICA-AROMA. Red = ICA-AROMA + 8 Phys (aggressive). Orange = ICA-AROMA + 8 Phys (non-aggressive).

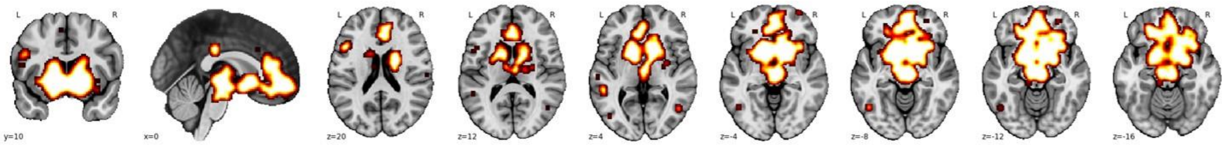

Supplementary Figure S3. Reward mask used in fMRI analyses. Downloaded from neurosynth.org on June 28, 2022. The NIfTI file can be found with the codes on OSF. This mask was defined based on 922 relevant studies associated with the term ‘reward’ (FDR = 0.01, association test with positive values, no further threshold). The resulting mask was binarized and encompassed a total of 12,969 voxels, providing a targeted substrate for our investigation.

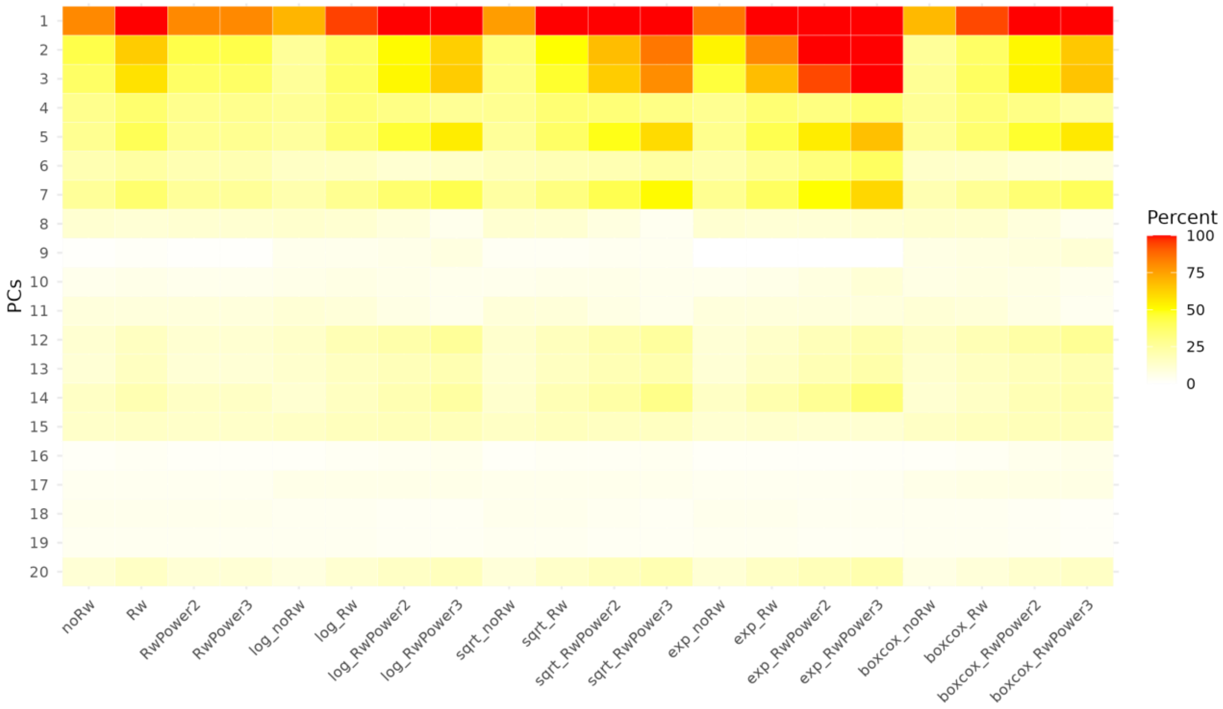

Supplementary Figure S4. Robustness of the first 20 PCs from the Feedback_Hit_HighReward contrast in models combining different reweighting and transformation strategies. Reweighting was applied according to the formula in the main Methods, with exponent *x* set to 0 (no reweighting, noRw), 1 (Rw, main analysis), 2 (RwPower2), and 3 (RwPower3). Higher exponents intensify the emphasis on uncommon

outcomes. These reweighting strategies were combined with the following outcome transformations: No transformation (other than standardization), log (natural logarithm), sqrt (square root), exp (natural exponential), and boxcox (Box-Cox with λ=0.5). Each transformation influenced the skewness (s) of the outcomes (pain and depression) as follows: no transformation: s_{pain} = 0.45, s_{dep} = 2.17; log: s_{pain} = - 0.53, s_{dep} = 1.07; sqrt: s_{pain} = - 0.02, s_{dep} = 1.56; exp: s_{pain} = 0.92, s_{dep} = 2.73; boxcox: s_{pain} = - 1.01, s_{dep} = 0.98. The model "Rw" corresponds to the one presented in the main Results. The robustness of the PCs was calculated by counting how many times they were selected when increasing the penalty of the MTL regularization procedure and retraining the model. The penalty term was increased, by steps of 0.1, from 0.1 to 10. The first PC is always the most robust feature for estimating pain and the severity of depressive symptoms.

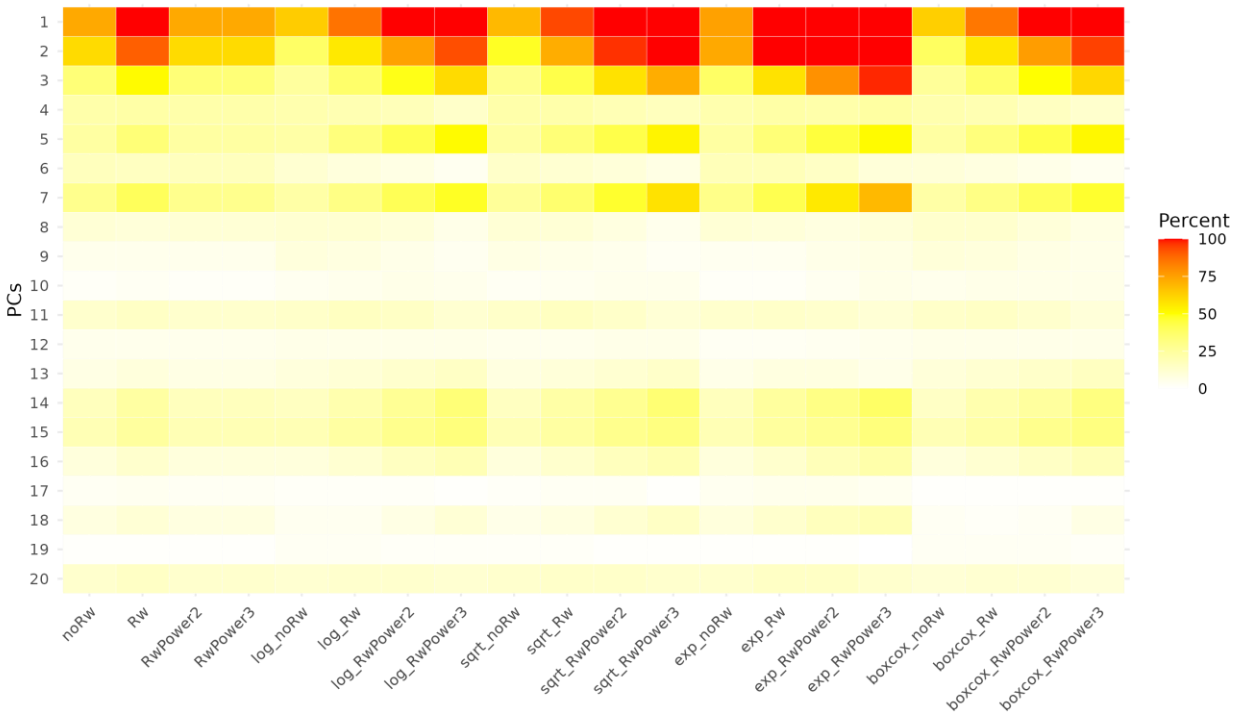

Supplementary Figure S5. Robustness of the first 20 PCs from the Feedback_Hit_LowReward contrast in models combining different reweighting and transformation strategies. See Supplementary Figure S4 for further details.

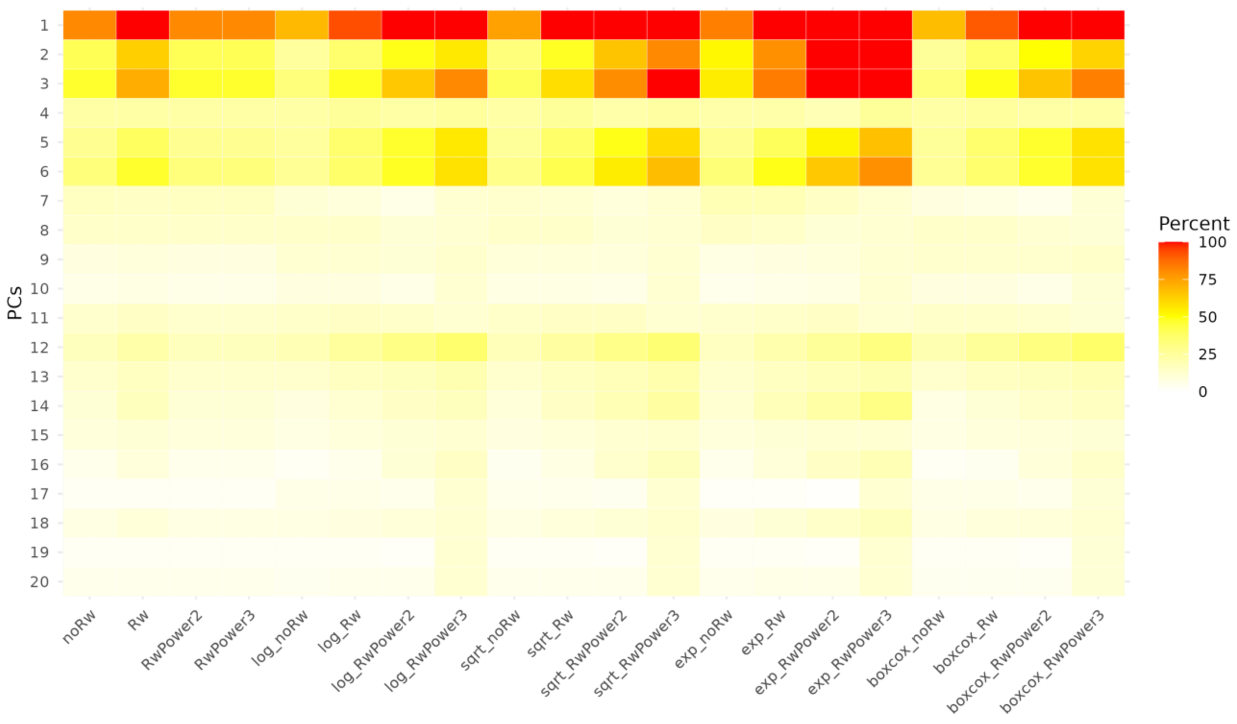

Supplementary Figure S6. Robustness of the first 20 PCs from the Feedback_HighReward contrast in models combining different reweighting and transformation strategies. See Supplementary Figure S4 for further details.

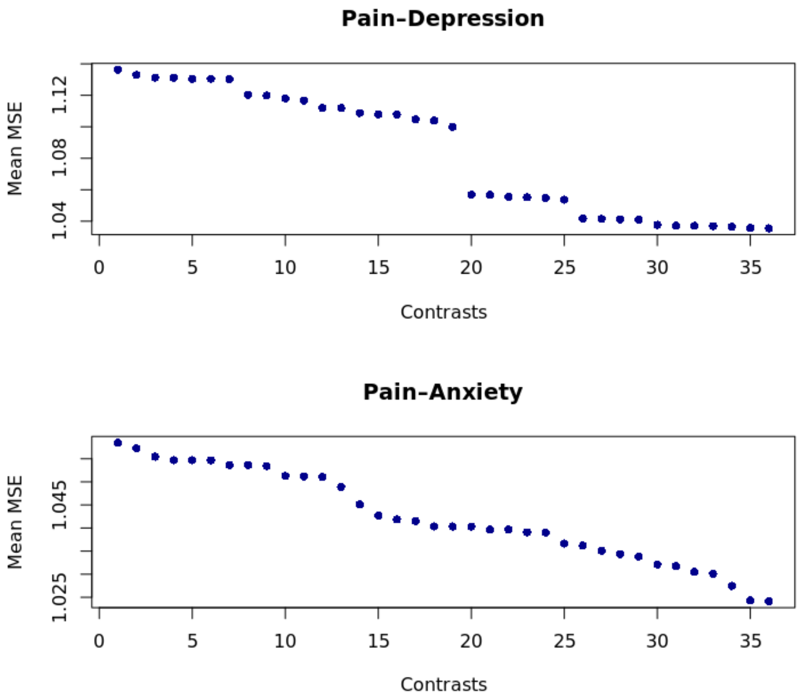

Supplementary Figure S7. Average mean squared errors for all the 36 contrasts, calculated during the leave-one-center-out cross-validation procedure in the training set.

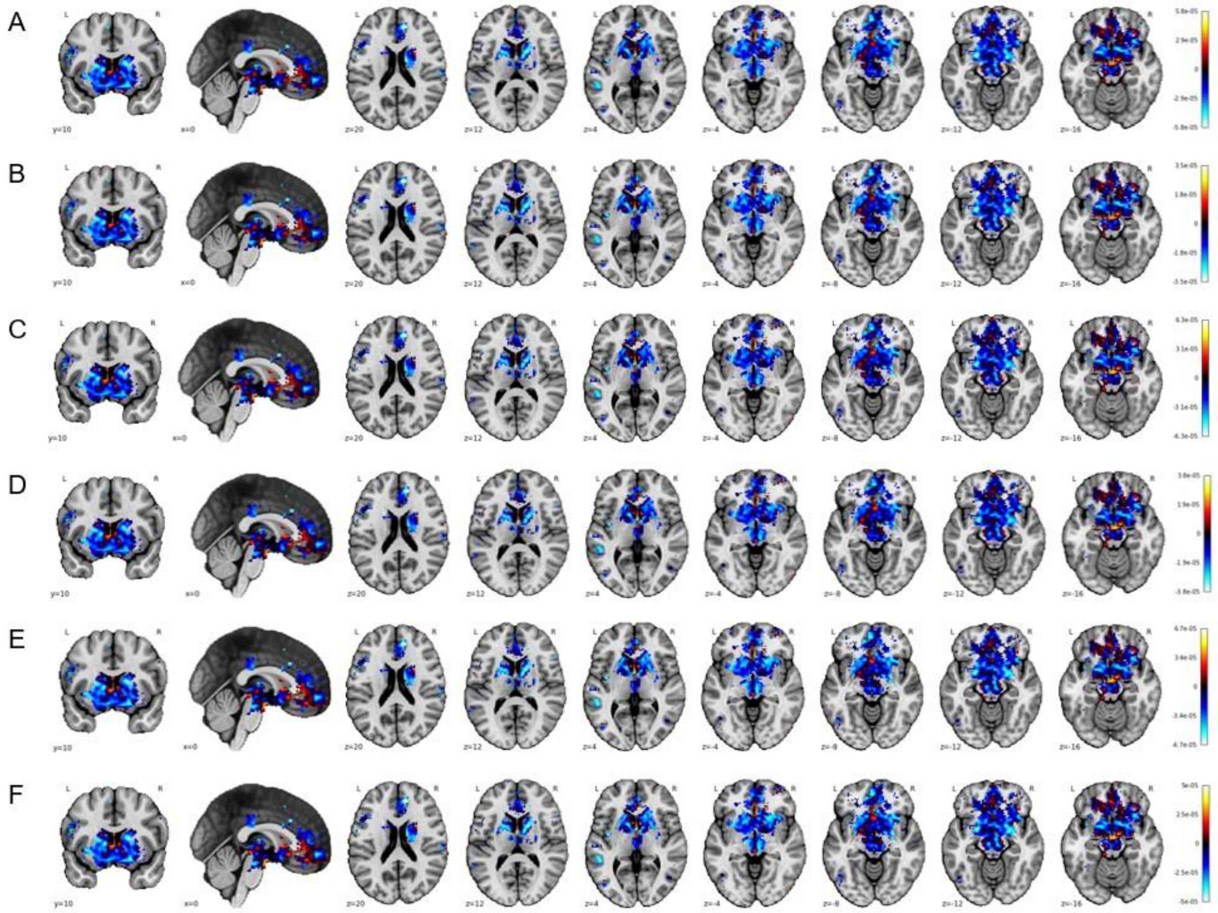

Supplementary Figure S8. Neural signatures from the three significant models of the IMAGEN sample. (**A**) Neural signature of the severity of depressive symptoms for the *Feedback_Hit_HighReward* contrast. (**B**) Neural signature of pain for the *Feedback_Hit_HighReward* contrast. (**C**) Neural signature of the severity of depressive symptoms for the *Feedback_Hit_LowReward* contrast. (**D**) Neural signature of pain for the *Feedback_Hit_LowReward* contrast. (**E**) Neural signature of the severity of depressive symptoms for the *Feedback_HighReward* contrast. (**F**) Neural signature of pain for the *Feedback_HighReward* contrast. The neural signatures were derived from three different BOLD contrasts, based on the BOLD response when: 1) the subjects hit the target and received a high reward (*Feedback_Hit_HighReward*); 2) the subjects hit the target and received a low reward (*Feedback_Hit_LowReward*); and 3) the subjects received a high reward (*Feedback_HighReward*). The three models were highly correlated (Pearson's correlation (r):

$r_{1\text{-}2}$ = 0.98, $r_{1\text{-}3}$ = 0.99, $r_{2\text{-}3}$ = 0.99). Each model contains two neural signatures, one consisting of the weights for estimating pain, and the other consisting of the weights for estimating the severity of depressive symptoms. Although features reflect the comorbidity of pain and the severity of depressive symptoms, the weights attributed to these common features may differ between these two signatures, and may have opposite signs. In our case, the signatures for pain and the severity of depressive symptoms were scaled versions of each other in all three significant models. This phenomenon was attributable to the exclusive selection of the first principal component in each model. All signatures are unmasked and unthresholded. Color scales indicate the weights of the signatures.

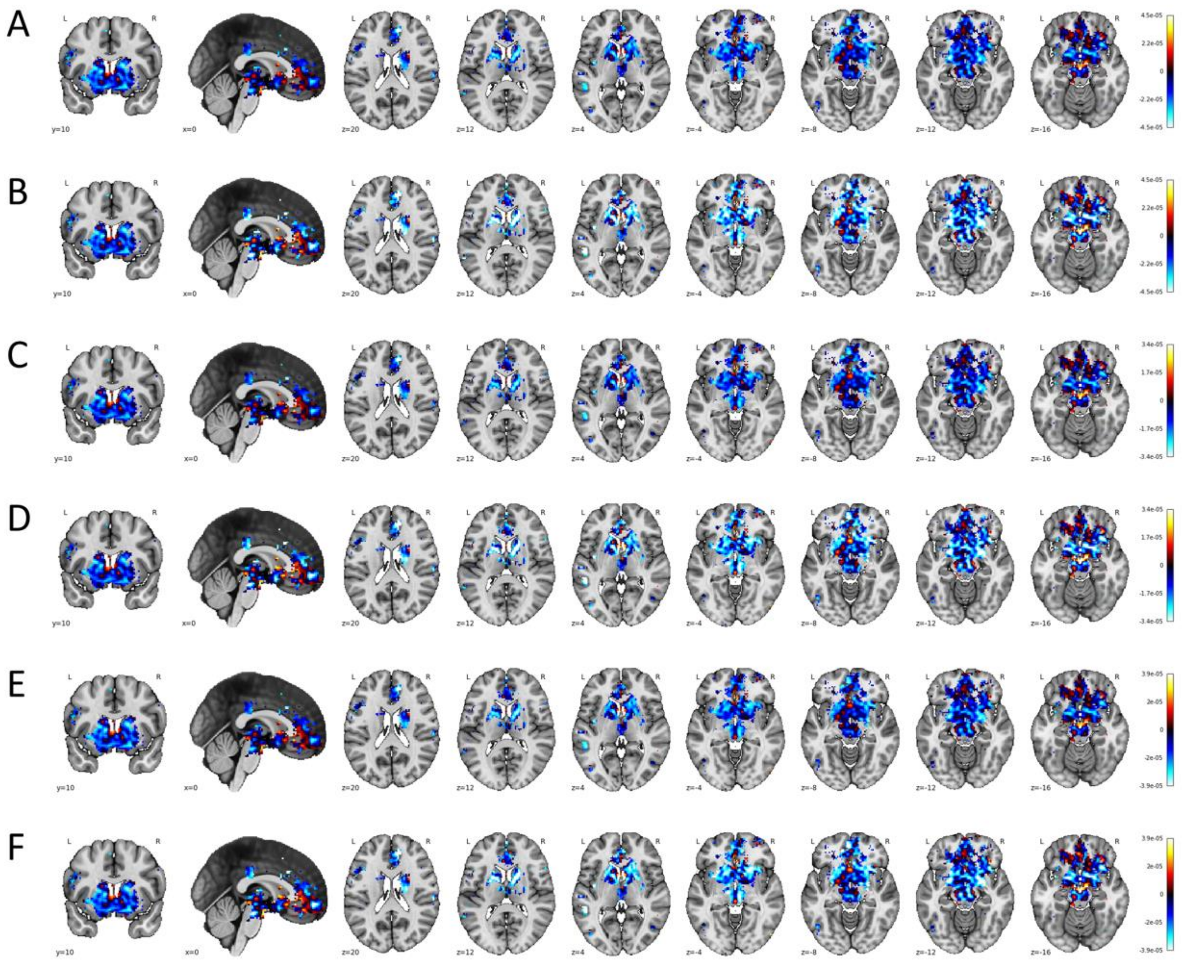

Supplementary Figure S9. Neural signatures of the comorbidity between pain and the severity of depressive symptoms, using sum scores of pain items as outcome measure for pain. Consistent with the main results, only the first principal component was selected, showing negative weights for both outcomes. (**A**) Neural signature of pain for the *Feedback_Hit_HighReward* contrast (MAE = [1.91; 2.12; 1.86; 2.19], P = [0.0007; 0.9983; 0.0041; 0.9995]). (**B**) Neural signature of the severity of depressive symptoms for the *Feedback_Hit_HighReward* contrast (MAE = [1.16; 1.71; 1.26; 1.79], P = [0.0020; 0.9985; 0.0014; 0.9980]). (**C**) Neural signature of pain for the *Feedback_Hit_LowReward* contrast (MAE = [1.91; 2.09; 1.86; 2.12], P = [0.0017; 0.0010; 0.0038; 0.9979]). (**D**) Neural signature of the severity of depressive symptoms for the *Feedback_Hit_LowReward* contrast (MAE = [1.18; 1.63; 1.28; 1.72], P = [0.0033; 0.9975; 0.0026; 0.9960]). (**E**) Neural signature of pain for the *Feedback_HighReward* contrast (MAE = [1.91; 2.10; 1.86; 2.18], P = [0.0010; 0.9973; 0.0067; 0.9989]). (**F**) Neural signature of the severity of depressive symptoms for the *Feedback_HighReward* contrast (MAE = [1.16; 1.67; 1.26; 1.73], P = [0.0035; 0.9946; 0.0039; 0.9965]). Color scales indicate the weights of the signatures.

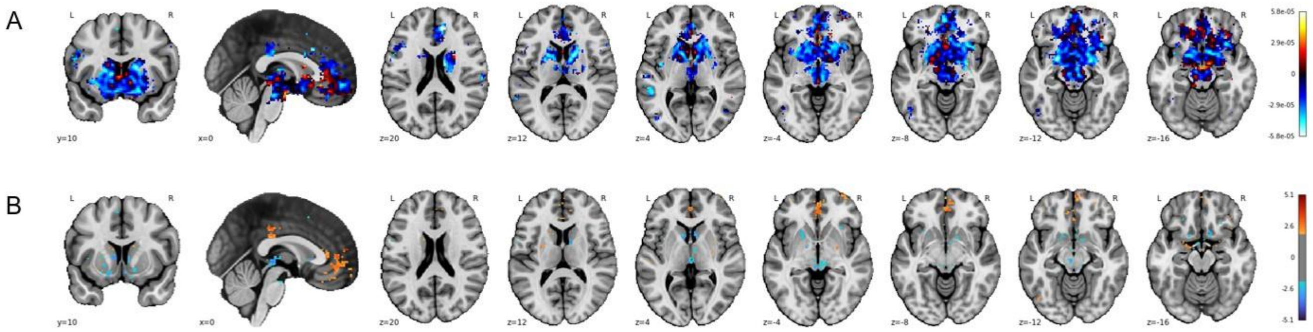

Supplementary Figure S10. Neural signature and group statistics for the contrast *Feedback_Hit_HighReward* of the IMAGEN sample. (**A**) Neural signature, unthresholded and unmasked. The signature shown is the one for the severity of depressive symptoms, the one for pain is identical with scaled values. (**B**) Group statistics with a significance level of p=0.05 and a threshold Z=1.96. Color scale indicates the z-values.

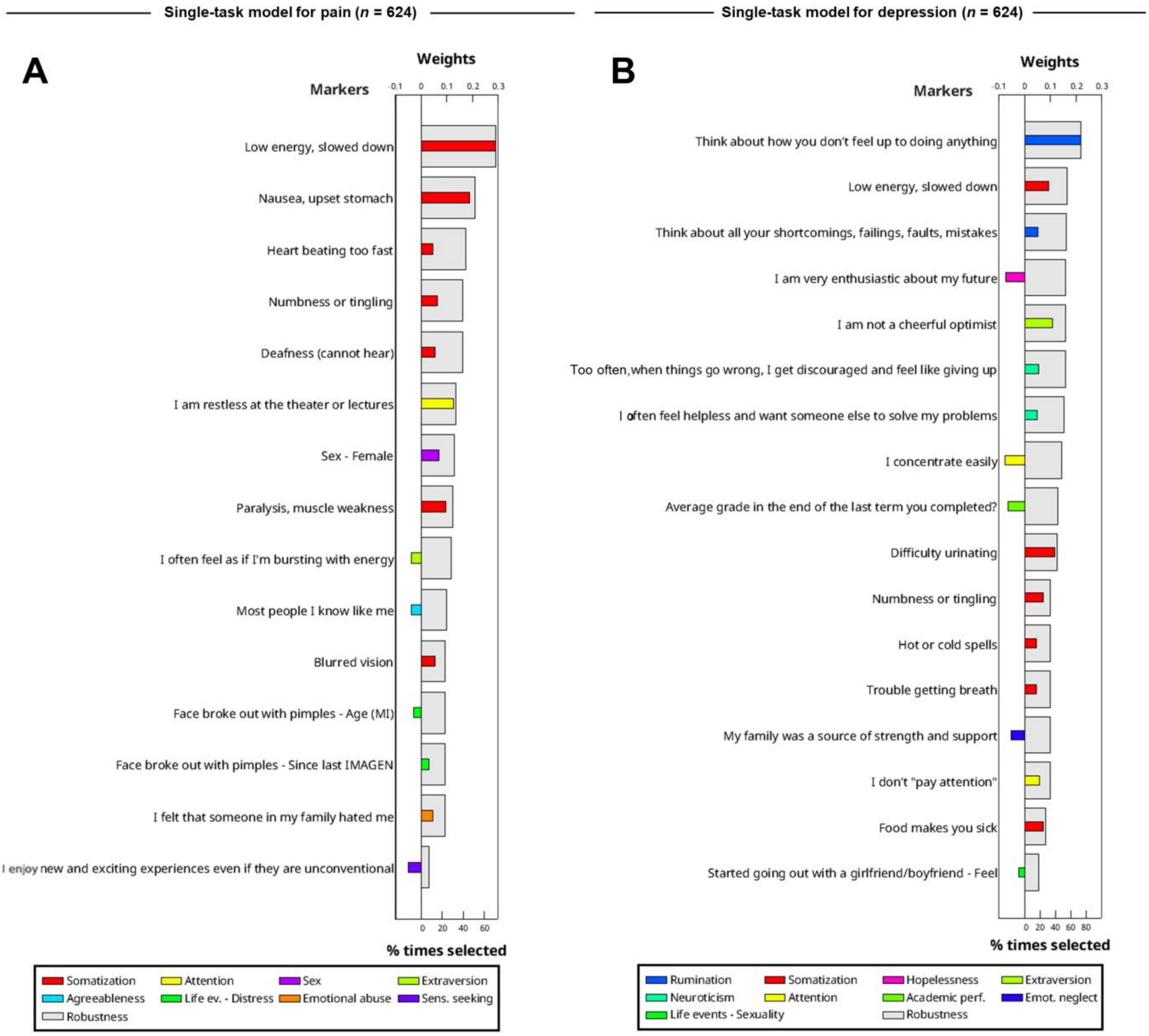

Supplementary Figure S11. Single-task psychosocial model for pain (A) and the severity of depressive symptoms (B). The presented features in **(A)** and **(B)** meet two criteria:

selected at least 10% of the times compared to the most robust feature, and have at least one weight that is a minimum of 10% of the highest weight. Grey bars indicate feature robustness, and colored bars show model weights. The upper x-axis shows weights, and the lower x-axis shows feature robustness. Labels and colors denote constructs and categories specified in the legend. Emot. = emotional; ev. = events; MI = missing indicator; perf. = performance; sens. = sensation.

Supplementary Tables

Supplementary Table S1. Demographic composition of the different centers of the IMAGEN sample used in this study. Data based on the sample used in the neuroimaging analysis.

	Paris	London	Nottingham	Dublin	Mannheim	Dresden	Hamburg	Berlin
**Sex (F)**	0.58	0.59	0.59	0.64	0.61	0.55	0.51	0.72
**Age**	19.7 +/- 0.7	18.9 +/- 0.5	18.8 +/- 0.5	19.2 +/- 0.7	19.0 +/- 0.8	18.6 +/- 0.6	18.8 +/- 0.6	19.1 +/- 0.9

Supplementary Table S2. DAWBA bands for different diagnoses in the IMAGEN sample. For each disorder, it is indicated the proportion of subjects in each band defined in the Development and Well-Being Assessment (DAWBA) [6]. Data based on the sample used in the neuroimaging analysis.

P(D)	MDD	GAD	SP	SAD	PD	AG	PTSD	OCD	ED
**< 0.1%**	0.557	0	0.657	0.737	0.959	0.911	0.885	0.830	0.614
**≈ 0.5%**	0.323	0.724	0.326	0.169	0.028	0.085	0.092	0.137	0.291
**≈ 3%**	0	0.175	0	0.048	0	0	0.010	0.018	0.083
**≈ 15%**	0.085	0.082	0.010	0.032	0.006	0.004	0.010	0.012	0
**≈ 50%**	0.019	0.019	0.007	0.013	0.007	0	0.003	0.003	0.012
**> 70%**	0.016	0	0	0	0	0	0	0	0

Abbreviations: P(D) = probability of diagnosis; MDD = major depression; GAD = generalized anxiety disorder; SP = specific phobia; SAD = social anxiety disorder (social phobia); PD = panic disorder; AG = agoraphobia; PTSD = post-traumatic stress disorder; OCD = obsessive compulsive disorder; ED = eating disorders.

Supplementary Table S3. Scanning parameters of the MID task for each center, from the follow-up 2 of the IMAGEN dataset. The selection of scanning variables was deliberately tailored to ensure compatibility across all scanning devices. Furthermore, imaging protocols were standardized consistently across various sites and scanners. Diverse centers used distinct 3 Tesla MRI scanners (Siemens, Munich, Germany; Philips, Best, The Netherlands; GE Healthcare, Chicago, USA), all adhering to a common set of parameters. Uniform hardware from Nordic Neurolabs in Bergen, Norway, was employed for the standardized presentation of visual and auditory stimuli across all sites. For all the centers, the functional MRI data entailed a repetition time (TR) of 2.2 seconds, an echo time (TE) of 30 milliseconds, and an in-plane resolution of 64 x 64 pixels. The imaging used a gradient-echo echo-planar (EPI) T2*-weighted sequence, encompassing the acquisition of 40 slices in a sequential descending order (with a slice thickness of 2.4 mm and a 1 mm gap), and an acceleration factor of 2.

	**Berlin**	**Dresden**	**Mannheim**	**Hamburg**	**London**	**Nottingham**	**Dublin**	**Paris**
**Scanner**	SIEMENS MAGNETOM Verio Syngo MR B17	SIEMENS MAGNETOM TrioTim Syngo MR B17	SIEMENS MAGNETOM TrioTim Syngo MR B17	SIEMENS MAGNETOM TrioTim Syngo MR B17	General Electric	PHILIPS	PHILIPS	SIEMENS MAGNETOM TrioTim Syngo MR B17
**TR (s)**	2.2	2.2	2.2	2.2	2.2	2.2	2.2	2.2
**TE (ms)**	30	30	30	30	30	30	30	30
**Matrix size**	64 x 64	64 x 64	64 x 64	64 x 64	64 x 64	64 x 64	64 x 64	64 x 64

**Acceleration factor**	2	2	2	2	2	2	2	2
**No. of slices**	40	40	40	40	40	40	40	40
**Slice thickness (mm)**	2.4	2.4	2.4	2.4	2.4	2.4	2.4	2.4
**Slice gap (mm)**	1.0	1.0	1.0	1.0	1.0	1.0	1.0	1.0
**Slice encoding direction**	Desc.	Desc.	Desc.	Desc.	Desc.	Desc.	Desc.	Desc.
**Slice acquisition mode**	Seq.	Seq.	Seq.	Seq.	Seq.	Seq.	Seq.	Seq.
**Phase encoding direction**	A>>P	P>>A	P>>A	P>>A	P>>A	A>>P	P>>A	P>>A
**Echo spacing (ms)**	0.52	0.58	0.58	0.58	0.424	0.5842	0.5842	0.58
**FoV (mm^2)**	220 x 220	220 x 220	220 x 220	220 x 220	220 x 220	220 x 220	220 x 220	220 x 220
**Flip angle (degrees)**	75	75	75	75	75	75	75	75
**Field map echo time difference (ms)**	2.46	2.46	2.46	2.46	2.27	5	5	2.46

Abbreviations: TR = time of repetition; TE = time of echo; Desc. = descending; Seq. = sequential; A = anterior; P = posterior; FoV = field of view.

Supplementary Table S4. Scanning parameters of the MPRAGE structural MRI for each center, from the follow-up 2 of the IMAGEN dataset. For structural reference, we used high-resolution anatomical MRIs, which employed a three-dimensional T1-weighted magnetization prepared gradient echo sequence (MPRAGE).

	Berlin	Dresden	Mannheim	Hamburg	London	Nottingham	Dublin	Paris
**Scanner**	SIEMENS MAGNETOM Verio Syngo MR B17	SIEMENS MAGNETOM TrioTim Syngo MR B17	SIEMENS MAGNETOM TrioTim Syngo MR B17	SIEMENS MAGNETOM TrioTim Syngo MR B17	General Electric	PHILIPS	PHILIPS	SIEMENS MAGNETOM TrioTim Syngo MR B17
**TR (s)**	2.3	2.3	2.3	2.3	2.3	2.3	2.3	2.3
**TE (ms)**	2.8	2.8	2.8	2.8	2.8	2.8	2.8	2.8
**Matrix size**	256 x 256	256 x 256	256 x 256	256 x 256	256 x 256	256 x 256	256 x 256	256 x 256
**No. of slices**	160	160	160	160	170	137	137	160
**Slice thickness (mm)**	1.1	1.1	1.1	1.1	1.1	1.1	1.1	1.1
**FoV (mm^2)**	263 x 280	263 x 280	263 x 280	263 x 280	263 x 280	263 x 281	263 x 281	263 x 280
**Flip angle (degrees)**	9	9	9	9	8	9	9	9

Abbreviations: TR = time of repetition; TE = time of echo; FoV = field of view.

Supplementary Table S5. The 36 contrasts computed from the MID task of the IMAGEN dataset. Each contrast was used to train a model for predicting the comorbidity between pain and the severity of depressive symptoms or between pain and the severity of anxiety symptoms.

Contrasts – Anticipation phase	Contrasts – Feedback phase
Anticipation_Hit_HighReward	Feedback_Hit_HighReward
Anticipation_Hit_NoReward	Feedback_Hit_NoReward
Anticipation_Hit_LowReward	Feedback_Hit_LowReward
Anticipation_Missed_HighReward	Feedback_Missed_HighReward
Anticipation_Missed_NoReward	Feedback_Missed_NoReward
Anticipation_Missed_LowReward	Feedback_Missed_LowReward
Anticipation_Hit	Feedback_Hit
Anticipation_Missed	Feedback_Missed
Anticipation_Hit > Anticipation_Missed	Feedback_Hit > Feedback_Missed
Anticipation_Hit_HighReward > Anticipation_Hit_NoReward	Feedback_Hit_HighReward > Feedback_Hit_NoReward
Anticipation_Hit_LowReward > Anticipation_Hit_NoReward	Feedback_Hit_LowReward > Feedback_Hit_NoReward
Anticipation_Missed_HighReward > Anticipation_Missed_NoReward	Feedback_Missed_HighReward > Feedback_Missed_NoReward
Anticipation_Missed_LowReward > Anticipation_Missed_NoReward	Feedback_Missed_LowReward > Feedback_Missed_NoReward
Anticipation_HighReward	Feedback_HighReward
Anticipation_LowReward	Feedback_LowReward
Anticipation_NoReward	Feedback_NoReward
Anticipation_HighReward > Anticipation_NoReward	Feedback_HighReward > Feedback_NoReward
Anticipation_LowReward > Anticipation_NoReward	Feedback_LowReward > Feedback_NoReward

Supplementary Table S6. Model performance (MAE and RMSE) for the Feedback_Hit_HighReward contrast, using various combinations of reweighting and transformation strategies. Reweighting was applied according to the formula in the main

Methods, with exponent *x* set to 0 (no reweighting, noRw), 1 (Rw, main analysis), 2 (RwPower2), and 3 (RwPower3). Higher exponents intensify the emphasis on uncommon outcomes. These reweighting strategies were combined with the following outcome transformations: No transformation (other than standardization), log (natural logarithm), sqrt (square root), exp (natural exponential), and boxcox (Box-Cox with λ=0.5). Each transformation influenced the skewness (s) of the outcomes (pain and severity of depressive symptoms) as follows: no transformation: s_{pain} = 0.45, s_{dep} = 2.17; log: s_{pain} = - 0.53, s_{dep} = 1.07; sqrt: s_{pain} = - 0.02, s_{dep} = 1.56; exp: s_{pain} = 0.92, s_{dep} = 2.73; boxcox: s_{pain} = - 1.01, s_{dep} = 0.98.

		Dresden		Dublin		Mannheim		Nottingham	
		**MAE**	**RMSE**	**MAE**	**RMSE**	**MAE**	**RMSE**	**MAE**	**RMSE**
**noRw**	**Pain**	0.57	0.72	0.69	0.91	0.59	0.78	0.82	1.15
	**Dep**	1.14	1.62	1.84	2.69	1.24	1.86	1.94	2.84
**Rw**	**Pain**	0.57	0.73	0.65	0.84	0.59	0.77	0.67	0.87
	**Dep**	1.17	1.63	1.69	2.51	1.27	1.87	1.75	2.45
**RwPower2**	**Pain**	0.57	0.72	0.69	0.88	0.59	0.78	0.72	0.94
	**Dep**	1.15	1.62	1.82	2.81	1.25	1.86	1.92	2.65
**RwPower3**	**Pain**	0.57	0.72	0.71	0.91	0.59	0.78	0.75	0.98
	**Dep**	1.13	1.62	2.02	3.26	1.23	1.86	2.16	2.99
**Log_noRw**	**Pain**	0,5	0,73	0,77	1,08	0,53	0,81	0,84	1,27
	**Dep**	0,94	1,59	1,69	2,64	1,09	1,88	1,7	2,69
**Log_Rw**	**Pain**	0,51	0,73	0,64	0,89	0,54	0,8	0,63	0,89
	**Dep**	1,13	1,62	1,54	2,38	1,24	1,86	1,6	2,4
**Log_RwPower2**	**Pain**	0,5	0,73	0,68	0,92	0,53	0,8	0,7	0,96
	**Dep**	0,95	1,59	1,77	3,04	1,09	1,88	1,63	2,55
**Log_RwPower3**	**Pain**	0,5	0,73	0,75	1,02	0,53	0,81	0,74	1

	**Dep**	0,95	1,59	1,72	2,89	1,09	1,88	1,63	2,54
**Sqrt_noRw**	**Pain**	0,53	0,72	0,71	0,96	0,56	0,79	0,81	1,11
	**Dep**	1	1,58	1,73	2,64	1,13	1,86	1,73	2,62
**Sqrt_Rw**	**Pain**	0,54	0,72	0,64	0,86	0,56	0,79	0,68	0,91
	**Dep**	1,01	1,58	1,65	2,62	1,14	1,86	1,66	2,48
**Sqrt_RwPower2**	**Pain**	0,53	0,72	0,7	0,92	0,56	0,79	0,72	0,96
	**Dep**	1	1,58	1,82	3,05	1,13	1,86	1,74	2,56
**Sqrt_RwPower3**	**Pain**	0,53	0,72	0,71	0,93	0,56	0,79	0,73	0,96
	**Dep**	1	1,58	1,92	3,36	1,13	1,86	1,8	2,63
**Exp_noRw**	**Pain**	0,61	0,74	1,73	1,93	0,6	0,77	0,66	0,83
	**Dep**	1,3	1,69	1,93	2,39	1,36	1,89	1,58	2,5
**Exp_Rw**	**Pain**	0,61	0,74	1,04	1,19	0,62	0,77	0,67	0,83
	**Dep**	1,29	1,68	1,5	2,47	1,36	1,89	12,18	12,4
**Exp_RwPower2**	**Pain**	0,61	0,74	1,22	1,37	0,62	0,77	0,67	0,83
	**Dep**	1,27	1,67	1,5	2,58	1,34	1,89	25,01	25,12
**Exp_RwPower3**	**Pain**	0,6	0,74	1,56	1,77	0,61	0,77	0,67	0,83
	**Dep**	1,25	1,66	1,51	2,78	1,31	1,88	2,26	2,54
**Boxcox_noRw**	**Pain**	0,46	0,75	0,83	1,18	0,51	0,84	0,85	1,27
	**Dep**	0,89	1,62	1,72	2,73	1,04	1,93	1,77	2,88
**Boxcox_Rw**	**Pain**	0,46	0,75	0,61	0,94	0,49	0,83	0,6	0,95
	**Dep**	1,03	1,59	1,49	2,41	1,16	1,85	1,56	2,44
**Boxcox_RwPower2**	**Pain**	0,46	0,74	0,72	0,97	0,5	0,83	0,73	1,01
	**Dep**	0,89	1,62	1,74	2,98	1,04	1,93	1,64	2,64
**Boxcox_RwPower3**	**Pain**	0,46	0,74	0,79	1,08	0,5	0,84	0,77	1,05
	**Dep**	0,89	1,62	1,71	2,91	1,04	1,93	1,63	2,63

Abbreviations: MAE = mean absolute error; RMSE = root mean squared error; dep = depression.

Supplementary Table S7. Same as Supplementary Table S5, but for the Feedback_Hit_LowReward contrast.

		Dresden		Dublin		Mannheim		Nottingham	
		MAE	RMSE	MAE	RMSE	MAE	RMSE	MAE	RMSE
noRw	Pain	0,57	0,72	0,71	0,95	0,59	0,78	0,73	0,95
	Dep	1,15	1,63	1,86	2,73	1,24	1,86	1,83	2,6
Rw	Pain	0,57	0,73	0,67	0,85	0,59	0,77	0,67	0,88
	Dep	1,18	1,64	1,66	2,48	1,28	1,87	1,76	2,51
RwPower2	Pain	0,57	0,72	0,69	0,89	0,59	0,78	0,71	0,93
	Dep	1,16	1,63	1,84	2,77	1,25	1,86	1,97	2,78
RwPower3	Pain	0,57	0,72	0,69	0,89	0,59	0,78	0,7	0,91
	Dep	1,16	1,63	1,83	2,74	1,25	1,86	1,95	2,74
Log_noRw	Pain	0,49	0,73	0,81	1,18	0,53	0,81	0,78	1,11
	Dep	0,95	1,59	1,75	2,72	1,09	1,88	1,66	2,63
Log_Rw	Pain	0,51	0,73	0,64	0,9	0,54	0,8	0,62	0,89
	Dep	1,13	1,62	1,55	2,38	1,24	1,86	1,6	2,4
Log_RwPower2	Pain	0,5	0,73	0,65	0,9	0,53	0,8	0,69	0,95
	Dep	0,95	1,59	1,64	2,65	1,09	1,88	1,64	2,58
Log_RwPower3	Pain	0,5	0,73	0,7	0,95	0,53	0,8	0,73	1
	Dep	0,95	1,59	1,8	3,04	1,09	1,88	1,68	2,63
Sqrt_noRw	Pain	0,53	0,72	0,74	1,01	0,56	0,79	0,75	1,01
	Dep	1	1,59	1,79	2,71	1,13	1,86	1,69	2,58

**Sqrt_Rw**	**Pain**	0,55	0,72	0,66	0,87	0,58	0,78	0,65	0,86
	**Dep**	1,36	1,71	1,69	2,35	1,43	1,92	1,72	2,35
**Sqrt_RwPower2**	**Pain**	0,54	0,72	0,65	0,87	0,56	0,79	0,69	0,92
	**Dep**	1,01	1,59	1,66	2,63	1,13	1,85	1,69	2,56
**Sqrt_RwPower3**	**Pain**	0,53	0,72	0,71	0,94	0,56	0,79	0,74	0,99
	**Dep**	1	1,59	1,83	2,93	1,13	1,86	1,77	2,64
**Exp_noRw**	**Pain**	0,6	0,73	1,53	1,73	0,61	0,77	0,67	0,83
	**Dep**	1,31	1,69	2,11	2,48	1,35	1,89	6,12	6,56
**Exp_Rw**	**Pain**	0,61	0,74	0,8	0,91	0,63	0,77	0,67	0,82
	**Dep**	1,33	1,7	1,69	2,35	1,39	1,9	3,38	4,11
**Exp_RwPower2**	**Pain**	0,61	0,74	0,94	1,1	0,62	0,77	0,67	0,83
	**Dep**	1,32	1,7	1,69	2,35	1,38	1,9	6,45	6,87
**Exp_RwPower3**	**Pain**	0,6	0,74	1,07	1,21	0,62	0,77	0,67	0,83
	**Dep**	1,3	1,69	1,61	2,36	1,35	1,89	16,26	16,43
**Boxcox_noRw**	**Pain**	0,47	0,75	0,88	1,26	0,5	0,84	0,83	1,18
	**Dep**	0,89	1,62	1,78	2,81	1,04	1,93	1,66	2,7
**Boxcox_Rw**	**Pain**	0,46	0,75	0,61	0,94	0,5	0,84	0,6	0,95
	**Dep**	1,03	1,59	1,49	2,41	1,16	1,85	1,56	2,44
**Boxcox_RwPower2**	**Pain**	0,46	0,74	0,68	0,95	0,5	0,83	0,72	1,01
	**Dep**	0,89	1,62	1,62	2,68	1,04	1,92	1,64	2,66
**Boxcox_RwPower3**	**Pain**	0,46	0,74	0,74	1	0,5	0,84	0,76	1,04
	**Dep**	0,89	1,62	1,77	3	1,04	1,93	1,73	2,81

Abbreviations: MAE = mean absolute error; RMSE = root mean squared error; dep = depression.

Supplementary Table S8. Same as Supplementary Table S5, but for the Feedback_HighReward contrast.

		Dresden		Dublin		Mannheim		Nottingham	
		MAE	RMSE	MAE	RMSE	MAE	RMSE	MAE	RMSE
**noRw**	**Pain**	0,56	0,72	0,73	0,94	0,59	0,78	0,76	1,01
	**Dep**	1,14	1,62	1,81	2,6	1,24	1,86	1,78	2,53
**Rw**	**Pain**	0,57	0,73	0,66	0,85	0,59	0,78	0,71	0,9
	**Dep**	1,16	1,63	1,71	2,55	1,26	1,86	1,78	2,47
**RwPower2**	**Pain**	0,57	0,72	0,66	0,85	0,59	0,78	0,71	0,91
	**Dep**	1,15	1,62	1,76	2,65	1,25	1,86	1,85	2,54
**RwPower3**	**Pain**	0,56	0,72	0,71	0,91	0,59	0,78	0,77	0,99
	**Dep**	1,13	1,62	1,97	3,08	1,23	1,86	2,1	2,85
**Log_noRw**	**Pain**	0,49	0,73	0,82	1,22	0,53	0,81	0,78	1,13
	**Dep**	0,95	1,59	1,63	2,59	1,08	1,88	1,63	2,57
**Log_Rw**	**Pain**	0,51	0,73	0,64	0,89	0,54	0,8	0,63	0,89
	**Dep**	1,13	1,62	1,54	2,38	1,24	1,86	1,6	2,4
**Log_RwPower2**	**Pain**	0,5	0,73	0,66	0,9	0,53	0,8	0,71	0,97
	**Dep**	0,95	1,59	1,65	2,73	1,09	1,88	1,62	2,53
**Log_RwPower3**	**Pain**	0,49	0,73	0,71	0,96	0,53	0,81	0,74	1,01
	**Dep**	0,95	1,59	1,6	2,63	1,09	1,88	1,61	2,52
**Sqrt_noRw**	**Pain**	0,53	0,72	0,75	1,02	0,56	0,79	0,76	1,04
	**Dep**	1	1,59	1,7	2,58	1,13	1,86	1,66	2,52
**Sqrt_Rw**	**Pain**	0,54	0,72	0,65	0,85	0,56	0,78	0,68	0,91
	**Dep**	1,01	1,58	1,58	2,52	1,14	1,86	1,63	2,46
**Sqrt_RwPower2**	**Pain**	0,53	0,72	0,68	0,9	0,56	0,79	0,73	0,97
	**Dep**	1	1,58	1,71	2,79	1,13	1,86	1,7	2,52

**Sqrt_RwPower3**	**Pain**	0,53	0,72	0,7	0,92	0,56	0,79	0,74	0,98
	**Dep**	1	1,58	1,81	3,07	1,13	1,86	1,78	2,59
**Exp_noRw**	**Pain**	0,6	0,73	1,78	1,97	0,61	0,77	0,66	0,83
	**Dep**	1,26	1,67	1,57	2,37	1,35	1,89	7,83	8,17
**Exp_Rw**	**Pain**	0,61	0,74	1,01	1,16	0,62	0,77	0,67	0,83
	**Dep**	1,29	1,68	1,6	2,36	1,37	1,9	8	8,33
**Exp_RwPower2**	**Pain**	0,61	0,74	1,43	1,6	0,62	0,77	0,67	0,83
	**Dep**	1,27	1,67	1,65	2,35	1,35	1,89	14,77	14,95
**Exp_RwPower3**	**Pain**	0,6	0,74	1,59	1,8	0,61	0,77	0,66	0,83
	**Dep**	1,24	1,66	1,61	2,36	1,32	1,88	2,42	2,67
**Boxcox_noRw**	**Pain**	0,47	0,75	0,86	1,27	0,51	0,84	0,81	1,19
	**Dep**	0,89	1,62	1,64	2,67	1,04	1,93	1,63	2,64
**Boxcox_Rw**	**Pain**	0,46	0,75	0,61	0,94	0,49	0,83	0,6	0,95
	**Dep**	1,03	1,59	1,49	2,41	1,16	1,85	1,56	2,44
**Boxcox_RwPower2**	**Pain**	0,46	0,74	0,69	0,94	0,5	0,83	0,73	1,02
	**Dep**	0,89	1,62	1,59	2,67	1,04	1,92	1,61	2,6
**Boxcox_RwPower3**	**Pain**	0,46	0,74	0,74	1	0,5	0,84	0,76	1,05
	**Dep**	0,89	1,62	1,61	2,7	1,04	1,92	1,62	2,61

Abbreviations: MAE = mean absolute error; RMSE = root mean squared error; dep = depression.

Supplementary Table S9. Deviations from the original analysis strategy contained in the pre-registration. For each deviation, it is indicated the original strategy, the one adopted in the final analysis, and the reasons for the change.

Original strategy	Final strategy	Reason for change

Outcomes of the models in the IMAGEN dataset will be the change scores between follow-up 2 and follow-up 3	Outcomes of the models in the IMAGEN dataset were the scores from follow-up 2	The scale assessing depression changed from follow-up 2 to follow-up 3
The specificity of the neural signature will be tested using stress scores	Not tested	1) Signatures not built for diagnostic purposes
The list of questionnaires for the psychosocial predictors in IMAGEN will include TCI-R (but not LEQ and CSI)	The list of questionnaires for the psychosocial predictors in IMAGEN included LEQ and CSI (but not TCI-R)	TCI-R contained too many missing values. We decided later to include also LEQ and the non-painful somatic symptoms from the CSI as predictors in the analysis, because they were considered relevant.
"We will test the importance of the fine-grained structure of the signatures by smoothing them with increasingly wider Gaussian filters, and eventually binarizing them. To test the redundancy of the decoders, we will also randomly sample the weights, using decreasing percentages"	Not done	Not necessary for the aim of our study
We will test the models obtained in the IMAGEN dataset on the CBP-PREDICT dataset	Not possible	The two datasets had different:<br><br>1) Questionnaires,<br>2) Tasks used for fMRI
The outcomes "will be binarized into two classes splitting the distributions at their median values"	We trained regression models instead of classification models	1) The outcomes of our models were suited for regression algorithms<br>2) The unbalance of the outcomes was difficult to be dealt with using standard methods for classification, because in our MTL

	model each subject had two outcomes

Supplementary Table S10. MAEs and p-values (p) for the three selected models of the comorbidity between pain and the severity of anxiety symptoms. Calculations conducted for each hold-out center within the IMAGEN dataset. P-values obtained via 10,000 one-sided permutation tests.

		**Dresden**		**Dublin**		**Mannheim**		**Nottingham**	
		**MAE**	**p**	**MAE**	**p**	**MAE**	**p**	**MAE**	**p**
**Feedback_Hit_HighReward**	**Pain**	0.72	0.55	0.92	0.67	0.75	0.99	1.06	1.00
	**Anxiety**	0.68	0.59	1.04	0.89	0.70	0.67	0.92	0.87
**Feedback_HighReward**	**Pain**	0.72	0.80	0.98	0.88	0.74	0.97	1.00	0.98
	**Anxiety**	0.67	0.23	1.00	0.91	0.70	0.45	0.90	0.84
**Feedback_Missed_LowReward > Feedback_Missed_NoReward**	**Pain**	0.72	0.94	1.17	0.98	0.76	0.93	0.90	0.89
	**Anxiety**	0.68	0.46	1.14	0.96	0.72	0.87	0.98	0.99

Supplementary Methods

Pre-processing of fMRI data.

The B0 distortion correction was specifically applied when the corresponding field map could be calculated. This was feasible for 499 out of the total 689 subjects utilized in the analyses, constituting 72% of the subject sample.

<u>fMRIPrep boilerplate:</u>

Results included in this manuscript come from preprocessing performed using *fMRIPrep* 21.0.1 (@fmriprep1; @fmriprep2; RRID:SCR_016216), which is based on *Nipype* 1.6.1 (@nipype1; @nipype2; RRID:SCR_002502).

Preprocessing of B0 inhomogeneity mappings:

A total of 1 fieldmaps were found available within the input BIDS structure for this particular subject. A *B0* nonuniformity map (or *fieldmap*) was estimated from the phase-drift map(s) measure with two consecutive GRE (gradient-recalled echo) acquisitions. The corresponding phase-map(s) were phase-unwrapped with `prelude` (FSL 6.0.5.1:57b01774).

Anatomical data preprocessing:

A total of 1 T1-weighted (T1w) images were found within the input BIDS dataset.The T1-weighted (T1w) image was corrected for intensity non-uniformity (INU) with `N4BiasFieldCorrection` [@n4], distributed with ANTs 2.3.3 [@ants, RRID:SCR_004757], and used as T1w-reference throughout the workflow. The T1w-reference was then skull-stripped with a *Nipype* implementation of the `antsBrainExtraction.sh` workflow (from ANTs), using OASIS30ANTs as target template. Brain tissue segmentation of cerebrospinal fluid (CSF),white-matter (WM) and gray-matter (GM) was performed on the brain-extracted T1w using `fast` [FSL 6.0.5.1:57b01774, RRID:SCR_002823, @fsl_fast]. Volume-based spatial normalization to two standard spaces (MNI152NLin2009cAsym, MNI152NLin6Asym) was performed through nonlinear registration with `antsRegistration` (ANTs 2.3.3), using brain-extracted versions of both T1w reference and the T1w template. The following templates were selected for spatial normalization: *ICBM 152 Nonlinear Asymmetrical template version 2009c* [@mni152nlin2009casym, RRID:SCR_008796; TemplateFlow ID: MNI152NLin2009cAsym], *FSL's MNI ICBM 152 non-linear 6th Generation Asymmetric Average Brain Stereotaxic Registration Model* [@mni152nlin6asym, RRID:SCR_002823; TemplateFlow ID: MNI152NLin6Asym].

Functional data preprocessing:

For each of the 2 BOLD runs found per subject (across all tasks and sessions), the following preprocessing was performed. First, a reference volume and its skull-stripped version were generated using a custom methodology of *fMRIPrep*. Head-motion parameters with respect to the BOLD reference (transformation matrices, and six

corresponding rotation and translation parameters) are estimated before any spatiotemporal filtering using `mcflirt` [FSL 6.0.5.1:57b01774, @mcflirt]. The estimated *fieldmap* was then aligned with rigid-registration to the target EPI (echo-planar imaging) reference run. The field coefficients were mapped on to the reference EPI using the transform. BOLD runs were slice-time corrected to 1.07s (0.5 of slice acquisition range 0s-2.15s) using `3dTshift` from AFNI [@afni, RRID:SCR_005927]. The BOLD reference was then co-registered to the T1w reference using `mri_coreg` (FreeSurfer) followed by `flirt` [FSL 6.0.5.1:57b01774, @flirt] with the boundary-based registration [@bbr] cost-function. Co-registration was configured with six degrees of freedom. Several confounding time-series were calculated based on the *preprocessed BOLD*: framewise displacement (FD), DVARS and three region-wise global signals. FD was computed using two formulations following Power (absolute sum of relative motions, @power_fd_dvars) and Jenkinson (relative root mean square displacement between affines, @mcflirt). FD and DVARS are calculated for each functional run, both using their implementations in *Nipype* [following the definitions by @power_fd_dvars]. The three global signals are extracted within the CSF, the WM, and the whole-brain masks. Additionally, a set of physiological regressors were extracted to allow for component-based noise correction [*CompCor*, @compcor]. Principal components are estimated after high-pass filtering the *preprocessed BOLD* time-series (using a discrete cosine filter with 128s cut-off) for the two *CompCor* variants: temporal (tCompCor) and anatomical (aCompCor). tCompCor components are then calculated from the top 2% variable voxels within the brain mask. For aCompCor, three probabilistic masks (CSF, WM and combined CSF+WM) are generated in anatomical space. The implementation differs from that of Behzadi et al. in that instead of eroding the masks by 2 pixels on BOLD space, the aCompCor masks are subtracted a mask of pixels that likely contain a volume fraction of GM. This mask is obtained by thresholding the corresponding partial volume map at 0.05, and it ensures components are not extracted from voxels containing a minimal fraction of GM. Finally, these masks are resampled into BOLD space and binarized by thresholding at 0.99 (as in the original implementation). Components are also calculated separately within the WM and CSF masks. For each CompCor decomposition, the *k* components with the largest singular values are retained, such that the retained components' time series are sufficient to explain 50 percent of variance across the nuisance mask (CSF, WM, combined, or temporal). The remaining components are dropped from consideration. The head-motion estimates calculated in the correction step were also placed within the corresponding confounds file. The confound time series derived from head motion estimates and global signals were expanded with the inclusion of temporal derivatives and quadratic terms for each [@confounds_satterthwaite_2013]. Frames that exceeded a threshold of 0.5 mm FD or 1.5 standardised DVARS were annotated as motion outliers. The BOLD time-series were resampled into standard space, generating a *preprocessed BOLD run in MNI152NLin2009cAsym space*. First, a reference volume and its skull-stripped version were generated using a custom methodology of *fMRIPrep*. Automatic removal of motion artifacts using independent component analysis [ICA-AROMA, @aroma] was performed on the *preprocessed BOLD on MNI space* time-series after removal of non-steady state volumes and spatial smoothing with an isotropic, Gaussian kernel of 6mm FWHM (full-width half-maximum). Corresponding "non-aggressively" denoised runs were produced after such smoothing. Additionally, the "aggressive" noise-regressors were collected and placed in the

corresponding confounds file. All resamplings can be performed with *a single interpolation step* by composing all the pertinent transformations (i.e. head-motion transform matrices, susceptibility distortion correction when available, and co-registrations to anatomical and output spaces). Gridded (volumetric) resamplings were performed using `antsApplyTransforms` (ANTs), configured with Lanczos interpolation to minimize the smoothing effects of other kernels [@lanczos]. Non-gridded (surface) resamplings were performed using `mri_vol2surf` (FreeSurfer).
Many internal operations of *fMRIPrep* use *Nilearn* 0.8.1 [@nilearn, RRID:SCR_001362], mostly within the functional processing workflow. For more details of the pipeline, see [the section corresponding to workflows in *fMRIPrep*'s documentation](https://fmriprep.readthedocs.io/en/latest/workflows.html "FMRIPrep's documentation").

Data denoising

We evaluated three distinct denoising approaches:

1) ICA-AROMA:

- In this case, we used the output files of fMRIPrep, which had been smoothed with an isotropic Gaussian kernel of 6mm FWHM (full-width half-maximum) and denoised with ICA-AROMA.

2) ICA-AROMA + 8 Phys (aggressive):

- Employing a general linear model (GLM), we regressed out 8 physiological confounds from the (smoothed) fMRIPrep output denoised with ICA-AROMA. These confounds included mean signals of WM and CSF, their derivatives, squares, and square derivatives.

3) ICA-AROMA + 8 Phys (non-aggressive):

- In this approach, we denoised the (non-smoothed) pre-processed output of fMRIPrep in a single step, adding the 8 physiological confounds to the independent components calculated by ICA-AROMA, encompassing both signal and noise components.

ICA-AROMA has been demonstrated to efficiently remove artefacts from the signal[12,14], and excluding subjects with more movements can introduce biases[3]. Therefore, we did not exclude subjects based on the amount of movement or artefacts present before the denoising step, but estimated the efficacy of our denoising procedure using four metrics: the QC-FC correlations, the percentage of significant QC-FC correlations, QC-FC distance-dependence, and loss of temporal degrees of freedom[12]. To compute these metrics we used the Dictionary of Functional Modes for brain imaging (DiFuMo) with 256 components[5]. These metrics allowed us to choose the data denoised through the ICA-AROMA + 8 Phys (non-aggressive) procedure, which was superior in 3 out of 4 metrics (for details, see Supplementary Fig. 7). Furthermore, these data were unsmoothed, potentially resulting in higher resolution.

Psychosocial data

The questionnaires and tests used were as follows: Cambridge Gambling Task (CGT; CANTAB®, Cambridge Cognition, 2019); Monetary Choice Questionnaire (MCQ)[8]; Passive Avoidance Learning Paradigm (PALP)[1]; Neuroticism-Extraversion-Openness Personality Inventory (NEO)[4]; Substance Use Risk Profile Scale (SURPS)[17]; European School Survey Project on Alcohol and Drugs (ESPAD)[7], which includes the Bully questionnaire; Barratt Impulsiveness Scale (BIS-11)[13]; Ruminative Responses Scale (RRS)[15]; Childhood Trauma Questionnaire (CTQ-SF)[2]; Life-Events Questionnaire (LEQ)[10]; items indicating non-painful somatic symptoms from the Children’s Somatization Inventory (CSI)[9,16]. The predictor items derived from the CSI were not used in the calculation of the outcome. The variable 'sex' was also included.

Data Files S1-S5

Data File S1. The 453 variables used as features to train the psychosocial models of the IMAGEN sample. For each variable, we specify the corresponding questionnaire or test in which it is found, along with the percentage of missing values within our sample. See also the IMAGEN documentation for more details (https://imagen-project.org/). Variables containing (MI) at the end of their name are missing indicators.

**Variable**	**Questionnaire / Test**	**Missing (%)**
I plan tasks carefully.	Barratt Impulsiveness Scale	0
I do things without thinking.	Barratt Impulsiveness Scale	0
I make-up my mind quickly.	Barratt Impulsiveness Scale	0
I am happy-go-lucky.	Barratt Impulsiveness Scale	0
I don’t "pay attention".	Barratt Impulsiveness Scale	0
I have "racing" thoughts.	Barratt Impulsiveness Scale	0
I plan trips well ahead of time.	Barratt Impulsiveness Scale	0
I am self controlled.	Barratt Impulsiveness Scale	0
I concentrate easily.	Barratt Impulsiveness Scale	0
I save regularly.	Barratt Impulsiveness Scale	0
I "squirm" at plays or lectures.	Barratt Impulsiveness Scale	0
I am a careful thinker.	Barratt Impulsiveness Scale	0
I plan for job security.	Barratt Impulsiveness Scale	0
I say things without thinking.	Barratt Impulsiveness Scale	0
I like to think about complex problems.	Barratt Impulsiveness Scale	0
I change jobs.	Barratt Impulsiveness Scale	0
I act "on impulse".	Barratt Impulsiveness Scale	0
I get easily bored when solving thought problems.	Barratt Impulsiveness Scale	0
I act on the spur of the moment.	Barratt Impulsiveness Scale	0
I am a steady thinker.	Barratt Impulsiveness Scale	0
I change residences.	Barratt Impulsiveness Scale	0
I buy things on impulse.	Barratt Impulsiveness Scale	0
I can only think about one thing at a time.	Barratt Impulsiveness Scale	0
I change hobbies.	Barratt Impulsiveness Scale	0
I spend or charge more than I earn.	Barratt Impulsiveness Scale	0
I often have extraneous thoughts when thinking.	Barratt Impulsiveness Scale	0
I am more interested in the present than the future.	Barratt Impulsiveness Scale	0
I am restless at the theater or lectures.	Barratt Impulsiveness Scale	0
I like puzzles.	Barratt Impulsiveness Scale	0
I am future oriented.	Barratt Impulsiveness Scale	0
Cambridge Gambling Task - Delay Aversion	Cambridge Gambling Task	0
Cambridge Gambling Task - Deliberation Time	Cambridge Gambling Task	0
Cambridge Gambling Task - Overall Proportion Bet	Cambridge Gambling Task	0
Cambridge Gambling Task - Quality of Decision Making	Cambridge Gambling Task	0
Cambridge Gambling Task - Risk Adjustment	Cambridge Gambling Task	0
Cambridge Gambling Task - Risk Taking	Cambridge Gambling Task	0

I didnt have enough to eat.	Childhood Trauma Questionnaire	0
There was nothing I wanted to change about my family.	Childhood Trauma Questionnaire	0
People in my family hit me so hard that it left me with bruises or marks.	Childhood Trauma Questionnaire	0
I was punished with a belt, a board, a cord, or some other hard object.	Childhood Trauma Questionnaire	0
People in my family looked out for each other.	Childhood Trauma Questionnaire	0
People in my family said hurtful or insulting things to me.	Childhood Trauma Questionnaire	0
I believe that I was physically abused.	Childhood Trauma Questionnaire	0
I had the perfect childhood.	Childhood Trauma Questionnaire	0
I got beaten so badly that it was noticed by someone like a teacher, neighbor, or doctor.	Childhood Trauma Questionnaire	0
I felt that someone in my family hated me.	Childhood Trauma Questionnaire	0
People I my family felt close to each other.	Childhood Trauma Questionnaire	0
I knew there was someone to take care of me and protect me.	Childhood Trauma Questionnaire	0
Someone tried to touch me in a sexual way, or tried to make me touch them.	Childhood Trauma Questionnaire	0
Someone threatened to hurt me or tell lies about me unless I did something sexual with them.	Childhood Trauma Questionnaire	0
I had the best family in the world.	Childhood Trauma Questionnaire	0
Someone tried to make me do sexual things or watch sexual things.	Childhood Trauma Questionnaire	0
Someone molested me.	Childhood Trauma Questionnaire	0
I believe that I was emotionally abused.	Childhood Trauma Questionnaire	0
There was someone to take me to the doctor if I needed it.	Childhood Trauma Questionnaire	0
I believe that I was sexually abused.	Childhood Trauma Questionnaire	0
My family was a source of strength and support.	Childhood Trauma Questionnaire	0
People in my family called me things like "stupid", "lazy" or "ugly".	Childhood Trauma Questionnaire	0
My parents were too drunk or high to take care of the family.	Childhood Trauma Questionnaire	0
There was someone in my family who helped me feel that I was important or special.	Childhood Trauma Questionnaire	0
I had to wear dirty clothes.	Childhood Trauma Questionnaire	0
I felt loved.	Childhood Trauma Questionnaire	0
I thought that my parents wished I had never been born.	Childhood Trauma Questionnaire	0
I got hit so hard by someone in my family that I had to see a doctor or go to the hospital.	Childhood Trauma Questionnaire	0
How many times IN YOUR WHOLE LIFETIME have you had five or more drinks in a row?	European School Survey Project on Alcohol and Drugs	0
How many times OVER THE LAST 12 MONTHS have you had five or more drinks in a row?	European School Survey Project on Alcohol and Drugs	17,1
How many times OVER THE LAST 30 DAYS have you had five or more drinks in a row?	European School Survey Project on Alcohol and Drugs	22,8
How likely is it that each of the following things would happen to you personally, if you drink alcohol? Feel relaxed.	European School Survey Project on Alcohol and Drugs	0
How likely is it that each of the following things would happen to you personally, if you drink alcohol? Get into trouble with police.	European School Survey Project on Alcohol and Drugs	0

How likely is it that each of the following things would happen to you personally, if you drink alcohol? Harm my health.	European School Survey Project on Alcohol and Drugs	0
How likely is it that each of the following things would happen to you personally, if you drink alcohol? Feel happy.	European School Survey Project on Alcohol and Drugs	0
How likely is it that each of the following things would happen to you personally, if you drink alcohol? Forget my problems.	European School Survey Project on Alcohol and Drugs	0
How likely is it that each of the following things would happen to you personally, if you drink alcohol? Not be able to stop drinking.	European School Survey Project on Alcohol and Drugs	0
How likely is it that each of the following things would happen to you personally, if you drink alcohol? Get a hangover.	European School Survey Project on Alcohol and Drugs	0
How likely is it that each of the following things would happen to you personally, if you drink alcohol? Feel more friendly and outgoing.	European School Survey Project on Alcohol and Drugs	0
How likely is it that each of the following things would happen to you personally, if you drink alcohol? Feel sick.	European School Survey Project on Alcohol and Drugs	0
On how many occasions IN YOUR WHOLE LIFETIME have you been drunk from drinking alcoholic beverages?	European School Survey Project on Alcohol and Drugs	0
On how many occasions OVER THE LAST 12 MONTHS have you been drunk from drinking alcoholic beverages?	European School Survey Project on Alcohol and Drugs	17,1
On how many occasions OVER THE LAST 30 DAYS have you been drunk from drinking alcoholic beverages?	European School Survey Project on Alcohol and Drugs	26,3
Please indicate on this scale from 1 to 10 how drunk you would say you were the last time you were drunk.	European School Survey Project on Alcohol and Drugs	17,1
Have you ever wanted to try any of the drugs mentioned in the previous questions?	European School Survey Project on Alcohol and Drugs	0
When did you FIRST drink alcopops (at least one bottle)?	European School Survey Project on Alcohol and Drugs	0
When did you FIRST drink beer (at least one glass)?	European School Survey Project on Alcohol and Drugs	0
When did you FIRST get drunk from drinking alcoholic beverages?	European School Survey Project on Alcohol and Drugs	17,1
When did you smoke your FIRST cigarette?	European School Survey Project on Alcohol and Drugs	36,2
When did you first smoke cigarettes ON A DAILY BASIS?	European School Survey Project on Alcohol and Drugs	36,2
When did you FIRST drink spirits (at least one shot)?	European School Survey Project on Alcohol and Drugs	0
When did you FIRST drink wine (at least one glass)?	European School Survey Project on Alcohol and Drugs	0
Please indicate if you have used amphetamines (speed), methamphetamine (crystal meth) or desoxyn on a weekly basis OVER A 12 MONTH PERIOD or in an excessive or problematic way IN YOUR LIFETIME?	European School Survey Project on Alcohol and Drugs	98,9
Please indicate if you have used marijuana (grass, pot) or hashish (hash, hash oil) on a weekly basis OVER A 12 MONTH PERIOD or in an excessive or problematic way IN YOUR LIFETIME?'	European School Survey Project on Alcohol and Drugs	80,9
Please indicate if you have used ketamine (Ket, K) or Phencyclidine (PCP, or angel dust) on a weekly basis OVER A 12 MONTH PERIOD or in an excessive or problematic way IN YOUR LIFETIME?	European School Survey Project on Alcohol and Drugs	99,4
Please indicate if you have used ecstasy (MDMA) on a weekly basis OVER A 12 MONTH PERIOD or in an excessive or problematic way IN YOUR LIFETIME?	European School Survey Project on Alcohol and Drugs	97,4
Average grade in the end of the last term you completed? (From 1=A or 93-100 to 8=C- or 70-72)	European School Survey Project on Alcohol and Drugs	0
On how many occasions during your lifetime have you smoked cigarettes?	European School Survey Project on Alcohol and Drugs	0
On how many occasions IN YOUR WHOLE LIFETIME have you had any alcoholic beverage to drink?	European School Survey Project on Alcohol and Drugs	0
On how many occasions OVER THE LAST 12 MONTHS have you had any alcoholic beverage to drink?	European School Survey Project on Alcohol and Drugs	0
On how many occasions OVER THE LAST 30 DAYS have you had any alcoholic beverage to drink?	European School Survey Project on Alcohol and Drugs	3,4

On how many occasions OVER THE LAST 30 DAYS have you had beer to drink?	European School Survey Project on Alcohol and Drugs	0
On how many occasions OVER THE LAST 30 DAYS have you had wine to drink?	European School Survey Project on Alcohol and Drugs	0
On how many occasions OVER THE LAST 30 DAYS have you had an alcopop to drink?	European School Survey Project on Alcohol and Drugs	0
On how many occasions OVER THE LAST 30 DAYS have you had spirits to drink?	European School Survey Project on Alcohol and Drugs	0
I was bullied at school/college/work (a student/ peer said or did nasty or unpleasant things to me).	Bully Questionnaire	0
I was called mean names, was made fun of, or teased in a hurtful way by a student/ peer.	Bully Questionnaire	0
A student/ peer left me out of things on purpose, excluded me from their group of friends or completely ignored me.	Bully Questionnaire	0
I was hit, kicked, pushed or shoved around, or locked indoors by a student/ peer.	Bully Questionnaire	0
I took part in bullying another student/ peer at school / college / work.	Bully Questionnaire	0
I called another student/ peer mean names, made fun of, or teased him or her in a hurtful way.	Bully Questionnaire	0
I kept a student/ peer out of things on purpose, excluded that student/peer from my group of friends, or completely ignored that student/ peer.	Bully Questionnaire	0
I hit, kicked, pushed, shoved around, or locked a student/ peer indoors.	Bully Questionnaire	0
I have been bullied by a teacher.	Bully Questionnaire	0
I have been bullied by a family member.	Bully Questionnaire	0
I have bullied a teacher.	Bully Questionnaire	0
I have bullied a family member.	Bully Questionnaire	0
When did you first try amphetamines (speed), methamphetamine (crystal meth) or desoxyn?	European School Survey Project on Alcohol and Drugs	98,9
When did you first try cocaine (coke)?	European School Survey Project on Alcohol and Drugs	99
When did you first try marijuana (grass, pot) or hashish (hash, hash oil)?	European School Survey Project on Alcohol and Drugs	80,9
When did you first try ketamine (Ket, K) or Phencyclidine (PCP, or angel dust)?	European School Survey Project on Alcohol and Drugs	99,4
When did you first try LSD (acid)?	European School Survey Project on Alcohol and Drugs	99,7
When did you first try ecstasy (MDMA)?	European School Survey Project on Alcohol and Drugs	97,4
When did you first try magic mushrooms or other hallucinogens (excluding LSD)?	European School Survey Project on Alcohol and Drugs	99,4
When did you first try narcotics (e.g. opium, morphine, codeine)?	European School Survey Project on Alcohol and Drugs	99,4
When did you first try tranquillisers or sedatives (without a doctors prescription) (benzodiazepines, valium or xanax; barbiturates, barbs or downers such as amytal, or seconal)?	European School Survey Project on Alcohol and Drugs	99,7
On how many occasions IN YOUR WHOLE LIFETIME have you used amphetamines (speed), methamphetamine (crystal meth) or desoxyn?	European School Survey Project on Alcohol and Drugs	0
On how many occasions IN YOUR WHOLE LIFETIME have you used cocaine (coke)?	European School Survey Project on Alcohol and Drugs	0
On how many occasions IN YOUR WHOLE LIFETIME have you used crack?	European School Survey Project on Alcohol and Drugs	0
On how many occasions IN YOUR WHOLE LIFETIME have you used GHB or liquid ecstasy?	European School Survey Project on Alcohol and Drugs	0
On how many occasions IN YOUR WHOLE LIFETIME have you sniffed a substance (glue, aerosols etc) to get high?	European School Survey Project on Alcohol and Drugs	0
On how many occasions IN YOUR WHOLE LIFETIME have you used marijuana (grass, pot) or hashish (hash, hash oil)?	European School Survey Project on Alcohol and Drugs	0
On how many occasions IN YOUR WHOLE LIFETIME have you used ketamine (Ket, K) or Phencyclidine (PCP, or angel dust)?	European School Survey Project on Alcohol and Drugs	0

On how many occasions IN YOUR WHOLE LIFETIME have you used LSD (acid)?	European School Survey Project on Alcohol and Drugs	0
On how many occasions IN YOUR WHOLE LIFETIME have you used ecstasy (MDMA)?	European School Survey Project on Alcohol and Drugs	0
On how many occasions IN YOUR WHOLE LIFETIME have you used magic mushrooms or other hallucinogens (excluding LSD)?	European School Survey Project on Alcohol and Drugs	0
On how many occasions IN YOUR WHOLE LIFETIME have you used narcotics (e.g. opium, morphine, codeine)?	European School Survey Project on Alcohol and Drugs	0
On how many occasions IN YOUR WHOLE LIFETIME have you used tranquillisers or sedatives (without a doctors prescription) (benzodiazepines, valium or xanax; barbiturates, barbs or downers such as amytal, or seconal)?	European School Survey Project on Alcohol and Drugs	0
On how many occasions OVER THE LAST 30 DAYS have you used amphetamines (speed), methamphetamine (crystal meth) or desoxyn?	European School Survey Project on Alcohol and Drugs	98,9
On how many occasions OVER THE LAST 30 DAYS have you used cocaine (coke)?	European School Survey Project on Alcohol and Drugs	99
On how many occasions OVER THE LAST 30 DAYS have you used marijuana (grass, pot) or hashish (hash, hash oil)?	European School Survey Project on Alcohol and Drugs	80,9
On how many occasions OVER THE LAST 30 DAYS have you used ketamine (Ket, K) or Phencyclidine (PCP, or angel dust)?	European School Survey Project on Alcohol and Drugs	99,4
On how many occasions OVER THE LAST 30 DAYS have you used LSD (acid)?	European School Survey Project on Alcohol and Drugs	99,7
On how many occasions OVER THE LAST 30 DAYS have you used ecstasy (MDMA)?	European School Survey Project on Alcohol and Drugs	97,4
On how many occasions OVER THE LAST 30 DAYS have you used magic mushrooms or other hallucinogens (excluding LSD)?	European School Survey Project on Alcohol and Drugs	99,4
On how many occasions OVER THE LAST 30 DAYS have you used narcotics (e.g. opium, morphine, codeine)?	European School Survey Project on Alcohol and Drugs	99,4
On how many occasions OVER THE LAST 30 DAYS have you used tranquillisers or sedatives (without a doctors prescription) (benzodiazepines, valium or xanax; barbiturates, barbs or downers such as amytal, or seconal)?	European School Survey Project on Alcohol and Drugs	99,7
How many drinks containing alcohol do you have on a TYPICAL DAY when you are drinking?	European School Survey Project on Alcohol and Drugs	0
On how many occasions OVER THE LAST WEEK have you used amphetamines (speed), methamphetamine (crystal meth) or desoxyn?	European School Survey Project on Alcohol and Drugs	99,2
On how many occasions OVER THE LAST WEEK have you used cocaine (coke)?	European School Survey Project on Alcohol and Drugs	99,5
On how many occasions OVER THE LAST WEEK have you used marijuana (grass, pot) or hashish (hash, hash oil)?	European School Survey Project on Alcohol and Drugs	85,1
On how many occasions OVER THE LAST WEEK have you used ecstasy (MDMA)?	European School Survey Project on Alcohol and Drugs	98,9
On how many occasions OVER THE LAST WEEK have you used narcotics (e.g. opium, morphine, codeine)?	European School Survey Project on Alcohol and Drugs	99,7
On how many occasions OVER THE LAST WEEK have you used tranquillisers or sedatives (without a doctors prescription) (benzodiazepines, valium or xanax; barbiturates, barbs or downers such as amytal, or seconal)?	European School Survey Project on Alcohol and Drugs	99,8
On how many occasions OVER THE LAST 12 MONTHS have you used amphetamines (speed), methamphetamine (crystal meth) or desoxyn?	European School Survey Project on Alcohol and Drugs	96
On how many occasions OVER THE LAST 12 MONTHS have you used cocaine (coke)?	European School Survey Project on Alcohol and Drugs	96
On how many occasions OVER THE LAST 12 MONTHS have you sniffed a substance (glue, aerosols etc) to get high?	European School Survey Project on Alcohol and Drugs	96,5
On how many occasions OVER THE LAST 12 MONTHS have you used marijuana (grass, pot) or hashish (hash, hash oil)?	European School Survey Project on Alcohol and Drugs	55,4
On how many occasions OVER THE LAST 12 MONTHS have you used ketamine (Ket, K) or Phencyclidine (PCP, or angel dust)?	European School Survey Project on Alcohol and Drugs	98,1

On how many occasions OVER THE LAST 12 MONTHS have you used LSD (acid)?	European School Survey Project on Alcohol and Drugs	97,9
On how many occasions OVER THE LAST 12 MONTHS have you used ecstasy (MDMA)?	European School Survey Project on Alcohol and Drugs	93,1
On how many occasions OVER THE LAST 12 MONTHS have you used magic mushrooms or other hallucinogens (excluding LSD)?	European School Survey Project on Alcohol and Drugs	96,6
On how many occasions OVER THE LAST 12 MONTHS have you used narcotics (e.g. opium, morphine, codeine)?	European School Survey Project on Alcohol and Drugs	98,6
On how many occasions OVER THE LAST 12 MONTHS have you used tranquillisers or sedatives (without a doctors prescription) (benzodiazepines,<br>valium or xanax; barbiturates, barbs or downers such as amytal, or seconal)?	European School Survey Project on Alcohol and Drugs	98,7
Discounting parameter	Monetary Choice Questionnaire	0
Discounting parameter - Small long delay reward	Monetary Choice Questionnaire	0
Discounting parameter - Medium long delay reward	Monetary Choice Questionnaire	0
Discounting parameter - Large long delay reward	Monetary Choice Questionnaire	0
I am intrigued by the patterns I find in art and nature.	NEO Personality Inventory	0
I really enjoy talking to people.	NEO Personality Inventory	0
I would rather cooperate with others than compete with them.	NEO Personality Inventory	0
I like to have a lot of people around me.	NEO Personality Inventory	0
I like to be where the action is.	NEO Personality Inventory	0
Poetry has little or no effect on me.	NEO Personality Inventory	0
I have a clear set of goals and work toward them in an orderly fashion.	NEO Personality Inventory	0
I often try new and foreign foods.	NEO Personality Inventory	0
I dont like to waste my time daydreaming.	NEO Personality Inventory	0
I often feel as if Im bursting with energy.	NEO Personality Inventory	0
Most people I know like me.	NEO Personality Inventory	0
I work hard to accomplish my goals.	NEO Personality Inventory	0
I am a cheerful, high-spirited person.	NEO Personality Inventory	0
Too often, when things go wrong, I get discouraged and feel like giving up.	NEO Personality Inventory	0
I am not a cheerful optimist.	NEO Personality Inventory	0
Sometimes when I am reading poetry or looking at a work of art, I feel a chill or wave of excitement.	NEO Personality Inventory	0
My life is fast-paced.	NEO Personality Inventory	0
I have little interest in speculating on the nature of the universe or human condition.	NEO Personality Inventory	0
I am a productive person who always gets the job done.	NEO Personality Inventory	0
I often feel helpless and want someone else to solve my problems.	NEO Personality Inventory	0
I am a very active person.	NEO Personality Inventory	0
I have a lot of intellectual curiosity	NEO Personality Inventory	0
I often enjoy playing with theories or abstract ideas	NEO Personality Inventory	0
I strive for excellence in everything I do.	NEO Personality Inventory	0
I laugh easily.	NEO Personality Inventory	0
Once I find the right way to do something, I stick to it.	NEO Personality Inventory	0
Score Punishment-Punishment - Missing a “right” or active number is Punished and responding to a “wrong” or passive number is Punished	Passive Avoidance Learning Paradigm	0

Score Reward-Punishment - Responding to a “right” or active number is Rewarded and responding to a “wrong” or passive number is Punished	Passive Avoidance Learning Paradigm	0
Score Reward-Reward - “Right” responding to an active number is Rewarded and correctly missing a “wrong” or passive number is Rewarded	Passive Avoidance Learning Paradigm	0
Mean Omission Error Punishment-Punishment - Missing a “right” or active number is Punished and responding to a “wrong” or passive number is Punished	Passive Avoidance Learning Paradigm	0
Mean Omission Error Reward-Punishment - Responding to a “right” or active number is Rewarded and responding to a “wrong” or passive number is Punished	Passive Avoidance Learning Paradigm	0
Mean Omission Error Reward-Reward - “Right” responding to an active number is Rewarded and correctly missing a “wrong” or passive number is Rewarded	Passive Avoidance Learning Paradigm	0
Mean Commission Error Punishment-Punishment - Missing a “right” or active number is Punished and responding to a “wrong” or passive number is Punished	Passive Avoidance Learning Paradigm	0
Mean Commission Error Reward-Punishment - Responding to a “right” or active number is Rewarded and responding to a “wrong” or passive number is Punished	Passive Avoidance Learning Paradigm	0
Mean Commission Error Reward-Reward - “Right” responding to an active number is Rewarded and correctly missing a “wrong” or passive number is Rewarded	Passive Avoidance Learning Paradigm	0
Reaction Time Punishment-Punishment - Missing a “right” or active number is Punished and responding to a “wrong” or passive number is Punished	Passive Avoidance Learning Paradigm	0
Reaction Time Reward-Punishment - Responding to a “right” or active number is Rewarded and responding to a “wrong” or passive number is Punished	Passive Avoidance Learning Paradigm	0
Reaction Time Reward-Reward - “Right” responding to an active number is Rewarded and correctly missing a “wrong” or passive number is Rewarded	Passive Avoidance Learning Paradigm	0
Think about a recent situation, wishing it had gone better.	Ruminative Responses Scale	0
Think about all your shortcomings, failings, faults, mistakes.	Ruminative Responses Scale	0
Think about how you don’t feel up to doing anything.	Ruminative Responses Scale	0
Think about how angry you are with yourself.	Ruminative Responses Scale	0
Think about how passive and unmotivated you feel.	Ruminative Responses Scale	0
I would like to learn how to drive a motorcycle.	Substance Use Risk Profile Scale	0
I feel proud of my accomplishments.	Substance Use Risk Profile Scale	0
I would enjoy hiking long distances in wild and uninhabited territory.	Substance Use Risk Profile Scale	0
I feel pleasant.	Substance Use Risk Profile Scale	0
I am very enthusiastic about my future.	Substance Use Risk Profile Scale	0
I often involve myself in situations that I later regret being involved in.	Substance Use Risk Profile Scale	0
I enjoy new and exciting experiences even if they are unconventional.	Substance Use Risk Profile Scale	0
I have faith that my future holds great promise.	Substance Use Risk Profile Scale	0
I like doing things that frighten me a little.	Substance Use Risk Profile Scale	0

Sex: Female	NA	0
Parents divorced - Since last IMAGEN	Life-Events Questionnaire	0
Parents divorced - Feel	Life-Events Questionnaire	0
Parents divorced - Age	Life-Events Questionnaire	94,1
Family accident or illness - Since last IMAGEN	Life-Events Questionnaire	0
Family accident or illness - Feel	Life-Events Questionnaire	0
Family accident or illness - Age	Life-Events Questionnaire	58,2
Found a new group of friends - Since last IMAGEN	Life-Events Questionnaire	0
Found a new group of friends - Feel	Life-Events Questionnaire	0
Found a new group of friends - Age	Life-Events Questionnaire	19,9
Got in trouble with the law - Since last IMAGEN	Life-Events Questionnaire	0
Got in trouble with the law - Feel	Life-Events Questionnaire	0
Got in trouble with the law - Age	Life-Events Questionnaire	93,6
Stole something valuable - Since last IMAGEN	Life-Events Questionnaire	0
Stole something valuable - Feel	Life-Events Questionnaire	0
Stole something valuable - Age	Life-Events Questionnaire	95
Given medication by physician - Since last IMAGEN	Life-Events Questionnaire	0
Given medication by physician - Feel	Life-Events Questionnaire	0
Given medication by physician - Age	Life-Events Questionnaire	44,4
Fell in love - Since last IMAGEN	Life-Events Questionnaire	0
Fell in love - Feel	Life-Events Questionnaire	0
Fell in love - Age	Life-Events Questionnaire	28,5
Death in family - Since last IMAGEN	Life-Events Questionnaire	0
Death in family - Feel	Life-Events Questionnaire	0
Death in family - Age	Life-Events Questionnaire	67,8
Face broke out with pimples - Since last IMAGEN	Life-Events Questionnaire	0
Face broke out with pimples - Feel	Life-Events Questionnaire	0
Face broke out with pimples - Age	Life-Events Questionnaire	67
Brother or sister moved out - Since last IMAGEN	Life-Events Questionnaire	0
Brother or sister moved out - Feel	Life-Events Questionnaire	0
Brother or sister moved out - Age	Life-Events Questionnaire	74,4
Started seeing a therapist - Since last IMAGEN	Life-Events Questionnaire	0
Started seeing a therapist - Feel	Life-Events Questionnaire	0
Started seeing a therapist - Age	Life-Events Questionnaire	94,9
Parent changed jobs - Since last IMAGEN	Life-Events Questionnaire	0
Parent changed jobs - Feel	Life-Events Questionnaire	0
Parent changed jobs - Age	Life-Events Questionnaire	81,3
Began a time-consuming hobby - Since last IMAGEN	Life-Events Questionnaire	0
Began a time-consuming hobby - Feel	Life-Events Questionnaire	0
Began a time-consuming hobby - Age	Life-Events Questionnaire	68,9
Got or made pregnant - Since last IMAGEN	Life-Events Questionnaire	0
Got or made pregnant - Feel	Life-Events Questionnaire	0
Got or made pregnant - Age	Life-Events Questionnaire	98,9
Decided about college / university - Since last IMAGEN	Life-Events Questionnaire	0
Decided about college / university - Feel	Life-Events Questionnaire	0
Decided about college / university - Age	Life-Events Questionnaire	34,9
Changed schools - Since last IMAGEN	Life-Events Questionnaire	0

Changed schools - Feel	Life-Events Questionnaire	0
Changed schools - Age	Life-Events Questionnaire	96,3
Joined a club or group - Since last IMAGEN	Life-Events Questionnaire	0
Joined a club or group - Feel	Life-Events Questionnaire	0
Joined a club or group - Age	Life-Events Questionnaire	61,4
Got in trouble at school - Since last IMAGEN	Life-Events Questionnaire	0
Got in trouble at school - Feel	Life-Events Questionnaire	0
Got in trouble at school - Age	Life-Events Questionnaire	89,3
Got or gave sexually transmitted disease - Since last IMAGEN	Life-Events Questionnaire	0
Got or gave sexually transmitted disease - Feel	Life-Events Questionnaire	0
Got or gave sexually transmitted disease - Age	Life-Events Questionnaire	97,8
Met a teacher I liked a lot - Since last IMAGEN	Life-Events Questionnaire	0
Met a teacher I liked a lot - Feel	Life-Events Questionnaire	0
Met a teacher I liked a lot - Age	Life-Events Questionnaire	41,3
Family had money problems - Since last IMAGEN	Life-Events Questionnaire	0
Family had money problems - Feel	Life-Events Questionnaire	0
Family had money problems - Age	Life-Events Questionnaire	85,6
Got own TV or computer - Since last IMAGEN	Life-Events Questionnaire	0
Got own TV or computer - Feel	Life-Events Questionnaire	0
Got own TV or computer - Age	Life-Events Questionnaire	38,9
Parents argued or fought - Since last IMAGEN	Life-Events Questionnaire	0
Parents argued or fought - Feel	Life-Events Questionnaire	0
Parents argued or fought - Age	Life-Events Questionnaire	67,5
Ran away from home - Since last IMAGEN	Life-Events Questionnaire	0
Ran away from home - Feel	Life-Events Questionnaire	0
Ran away from home - Age	Life-Events Questionnaire	97,4
Started going out with a girlfriend/boyfriend - Since last IMAGEN	Life-Events Questionnaire	0
Started going out with a girlfriend/boyfriend - Feel	Life-Events Questionnaire	0
Started going out with a girlfriend/boyfriend - Age	Life-Events Questionnaire	33,5
Got poor grades in school - Since last IMAGEN	Life-Events Questionnaire	0
Got poor grades in school - Feel	Life-Events Questionnaire	0
Got poor grades in school - Age	Life-Events Questionnaire	50,3
Went on holiday without parents - Since last IMAGEN	Life-Events Questionnaire	0
Went on holiday without parents - Feel	Life-Events Questionnaire	0
Went on holiday without parents - Age	Life-Events Questionnaire	25,3
Started driving a motor vehicle - Since last IMAGEN	Life-Events Questionnaire	0
Started driving a motor vehicle - Feel	Life-Events Questionnaire	0
Started driving a motor vehicle - Age	Life-Events Questionnaire	38,6
Broke up with boy/ girl-friend - Since last IMAGEN	Life-Events Questionnaire	0
Broke up with boy/ girl-friend - Feel	Life-Events Questionnaire	0
Broke up with boy/ girl-friend - Age	Life-Events Questionnaire	56,4
Family moved - Since last IMAGEN	Life-Events Questionnaire	0
Family moved - Feel	Life-Events Questionnaire	0
Family moved - Age	Life-Events Questionnaire	94,6
Started making own money - Since last IMAGEN	Life-Events Questionnaire	0
Started making own money - Feel	Life-Events Questionnaire	0
Started making own money - Age	Life-Events Questionnaire	33,7

Found religion - Since last IMAGEN	Life-Events Questionnaire	0
Found religion - Feel	Life-Events Questionnaire	0
Found religion - Age	Life-Events Questionnaire	94,7
Parent remarried - Since last IMAGEN	Life-Events Questionnaire	0
Parent remarried - Feel	Life-Events Questionnaire	0
Parent remarried - Age	Life-Events Questionnaire	95,7
Had a gay experience - Since last IMAGEN	Life-Events Questionnaire	0
Had a gay experience - Feel	Life-Events Questionnaire	0
Had a gay experience - Age	Life-Events Questionnaire	88,1
Gained a lot of weight - Since last IMAGEN	Life-Events Questionnaire	0
Gained a lot of weight - Feel	Life-Events Questionnaire	0
Gained a lot of weight - Age	Life-Events Questionnaire	81,9
Serious accident or illness - Since last IMAGEN	Life-Events Questionnaire	0
Serious accident or illness - Feel	Life-Events Questionnaire	0
Serious accident or illness - Age	Life-Events Questionnaire	88,5
Lost virginity - Since last IMAGEN	Life-Events Questionnaire	0
Lost virginity - Feel	Life-Events Questionnaire	0
Lost virginity - Age	Life-Events Questionnaire	48,9
Parent abused alcohol - Since last IMAGEN	Life-Events Questionnaire	0
Parent abused alcohol - Feel	Life-Events Questionnaire	0
Parent abused alcohol - Age	Life-Events Questionnaire	97,1
Parents divorced - Age (MI)	Life-Events Questionnaire	NA
Family accident or illness - Age (MI)	Life-Events Questionnaire	NA
Found a new group of friends - Age (MI)	Life-Events Questionnaire	NA
Got in trouble with the law - Age (MI)	Life-Events Questionnaire	NA
Stole something valuable - Age (MI)	Life-Events Questionnaire	NA
Given medication by physician - Age (MI)	Life-Events Questionnaire	NA
Fell in love - Age (MI)	Life-Events Questionnaire	NA
Death in family - Age (MI)	Life-Events Questionnaire	NA
Face broke out with pimples - Age (MI)	Life-Events Questionnaire	NA
Brother or sister moved out - Age (MI)	Life-Events Questionnaire	NA
Started seeing a therapist - Age (MI)	Life-Events Questionnaire	NA
Parent changed jobs - Age (MI)	Life-Events Questionnaire	NA
Began a time-consuming hobby - Age (MI)	Life-Events Questionnaire	NA
Got or made pregnant - Age (MI)	Life-Events Questionnaire	NA
Decided about college / university - Age (MI)	Life-Events Questionnaire	NA
Changed schools - Age (MI)	Life-Events Questionnaire	NA
Joined a club or group - Age (MI)	Life-Events Questionnaire	NA
Got in trouble at school - Age (MI)	Life-Events Questionnaire	NA
Got or gave sexually transmitted disease - Age (MI)	Life-Events Questionnaire	NA
Met a teacher I liked a lot - Age (MI)	Life-Events Questionnaire	NA
Family had money problems - Age (MI)	Life-Events Questionnaire	NA
Got own TV or computer - Age (MI)	Life-Events Questionnaire	NA
Parents argued or fought - Age (MI)	Life-Events Questionnaire	NA
Ran away from home - Age (MI)	Life-Events Questionnaire	NA
Started going out with a girlfriend/boyfriend - Age (MI)	Life-Events Questionnaire	NA
Got poor grades in school - Age (MI)	Life-Events Questionnaire	NA

Went on holiday without parents - Age (MI)	Life-Events Questionnaire	NA
Started driving a motor vehicle - Age (MI)	Life-Events Questionnaire	NA
Broke up with boy/ girl-friend - Age (MI)	Life-Events Questionnaire	NA
Family moved - Age (MI)	Life-Events Questionnaire	NA
Started making own money - Age (MI)	Life-Events Questionnaire	NA
Found religion - Age (MI)	Life-Events Questionnaire	NA
Parent remarried - Age (MI)	Life-Events Questionnaire	NA
Had a gay experience - Age (MI)	Life-Events Questionnaire	NA
Gained a lot of weight - Age (MI)	Life-Events Questionnaire	NA
Serious accident or illness - Age (MI)	Life-Events Questionnaire	NA
Lost virginity - Age (MI)	Life-Events Questionnaire	NA
Parent abused alcohol - Age (MI)	Life-Events Questionnaire	NA
Lump in throat.	Children's Somatization Inventory	0
Weakness.	Children's Somatization Inventory	0
Heavy feelings in arms, legs.	Children's Somatization Inventory	0
Nausea, upset stomach.	Children's Somatization Inventory	0
Constipation.	Children's Somatization Inventory	0
Loose BM's, diarrhea.	Children's Somatization Inventory	0
Heart beating too fast.	Children's Somatization Inventory	0
Difficulty swallowing.	Children's Somatization Inventory	0
Losing voice.	Children's Somatization Inventory	0
Faintness or dizziness.	Children's Somatization Inventory	0
Deafness (cannot hear).	Children's Somatization Inventory	0
Double vision.	Children's Somatization Inventory	0
Blurred vision.	Children's Somatization Inventory	0
Blindness.	Children's Somatization Inventory	0
Fainting, passing out.	Children's Somatization Inventory	0
Memory loss, amnesia.	Children's Somatization Inventory	0
Seizures, convulsions.	Children's Somatization Inventory	0
Trouble walking.	Children's Somatization Inventory	0
Paralysis, muscle weakness.	Children's Somatization Inventory	0
Difficulty urinating.	Children's Somatization Inventory	0
Vomiting, throwing up.	Children's Somatization Inventory	0
Feeling bloated, gassy.	Children's Somatization Inventory	0
Food makes you sick.	Children's Somatization Inventory	0
Low energy, slowed down.	Children's Somatization Inventory	0

Trouble getting breath.	Children's Somatization Inventory	0
Hot or cold spells.	Children's Somatization Inventory	0
Numbness or tingling.	Children's Somatization Inventory	0
How many times OVER THE LAST 12 MONTHS have you had five or more drinks in a row? (MI)	European School Survey Project on Alcohol and Drugs	NA
How many times OVER THE LAST 30 DAYS have you had five or more drinks in a row? (MI)	European School Survey Project on Alcohol and Drugs	NA
On how many occasions OVER THE LAST 12 MONTHS have you been drunk from drinking alcoholic beverages? (MI)	European School Survey Project on Alcohol and Drugs	NA
On how many occasions OVER THE LAST 30 DAYS have you been drunk from drinking alcoholic beverages? (MI)	European School Survey Project on Alcohol and Drugs	NA
Please indicate on this scale from 1 to 10 how drunk you would say you were the last time you were drunk. (MI)	European School Survey Project on Alcohol and Drugs	NA
When did you FIRST get drunk from drinking alcoholic beverages? (MI)	European School Survey Project on Alcohol and Drugs	NA
When did you smoke your FIRST cigarette? (MI)	European School Survey Project on Alcohol and Drugs	NA
When did you first smoke cigarettes ON A DAILY BASIS? (MI)	European School Survey Project on Alcohol and Drugs	NA
Please indicate if you have used amphetamines (speed), methamphetamine (crystal meth) or desoxyn on a weekly basis OVER A 12 MONTH PERIOD or in an excessive or problematic way IN YOUR LIFETIME? (MI)	European School Survey Project on Alcohol and Drugs	NA
Please indicate if you have used cocaine (coke) on a weekly basis OVER A 12 MONTH PERIOD or in an excessive or problematic way IN YOUR LIFETIME? (MI)	European School Survey Project on Alcohol and Drugs	NA
Please indicate if you have used marijuana (grass, pot) or hashish (hash, hash oil) on a weekly basis OVER A 12 MONTH PERIOD or in an excessive or problematic way IN YOUR LIFETIME? (MI)	European School Survey Project on Alcohol and Drugs	NA
Please indicate if you have used ketamine (Ket, K) or Phencyclidine (PCP, or angel dust) on a weekly basis OVER A 12 MONTH PERIOD or in an excessive or problematic way IN YOUR LIFETIME? (MI)	European School Survey Project on Alcohol and Drugs	NA
Please indicate if you have used LSD on a weekly basis OVER A 12 MONTH PERIOD or in an excessive or problematic way IN YOUR LIFETIME? (MI)	European School Survey Project on Alcohol and Drugs	NA
Please indicate if you have used ecstasy (MDMA) on a weekly basis OVER A 12 MONTH PERIOD or in an excessive or problematic way IN YOUR LIFETIME? (MI)	European School Survey Project on Alcohol and Drugs	NA
Please indicate if you have used magic mushrooms or other hallucinogens (excluding LSD) on a weekly basis OVER A 12 MONTH PERIOD or in an excessive or problematic way IN YOUR LIFETIME? (MI)	European School Survey Project on Alcohol and Drugs	NA
Please indicate if you have used narcotics (e.g. opium, morphine, codeine) on a weekly basis OVER A 12 MONTH PERIOD or in an excessive or problematic way IN YOUR LIFETIME? (MI)	European School Survey Project on Alcohol and Drugs	NA
Please indicate if you have used tranquillisers or sedatives (without a doctors prescription) on a weekly basis OVER A 12 MONTH PERIOD or in an excessive or problematic way IN YOUR LIFETIME? (MI)	European School Survey Project on Alcohol and Drugs	NA
On how many occasions OVER THE LAST 30 DAYS have you had any alcoholic beverage to drink? (MI)	European School Survey Project on Alcohol and Drugs	NA
When did you first try amphetamines (speed), methamphetamine (crystal meth) or desoxyn? (MI)	European School Survey Project on Alcohol and Drugs	NA
When did you first try cocaine (coke)? (MI)	European School Survey Project on Alcohol and Drugs	NA
When did you first try marijuana (grass, pot) or hashish (hash, hash oil)? (MI)	European School Survey Project on Alcohol and Drugs	NA

When did you first try ketamine (Ket, K) or Phencyclidine (PCP, or angel dust)? (MI)	European School Survey Project on Alcohol and Drugs	NA
On how many occasions IN YOUR WHOLE LIFETIME have you used LSD (acid)? (MI)	European School Survey Project on Alcohol and Drugs	NA
When did you first try ecstasy (MDMA)? (MI)	European School Survey Project on Alcohol and Drugs	NA
When did you first try magic mushrooms or other hallucinogens (excluding LSD)?	European School Survey Project on Alcohol and Drugs	NA
When did you first try narcotics (e.g. opium, morphine, codeine)? (MI)	European School Survey Project on Alcohol and Drugs	NA
When did you first try tranquillisers or sedatives (without a doctors prescription) (benzodiazepines,<br>valium or xanax; barbiturates, barbs or downers such as amytal, or seconal)? (MI)	European School Survey Project on Alcohol and Drugs	NA
On how many occasions OVER THE LAST 30 DAYS have you used amphetamines (speed), methamphetamine (crystal meth) or desoxyn? (MI)	European School Survey Project on Alcohol and Drugs	NA
On how many occasions OVER THE LAST 30 DAYS have you used cocaine (coke)? (MI)	European School Survey Project on Alcohol and Drugs	NA
On how many occasions OVER THE LAST 30 DAYS have you used marijuana (grass, pot) or hashish (hash, hash oil)? (MI)	European School Survey Project on Alcohol and Drugs	NA
On how many occasions OVER THE LAST 30 DAYS have you used ketamine (Ket, K) or Phencyclidine (PCP, or angel dust)? (MI)	European School Survey Project on Alcohol and Drugs	NA
On how many occasions OVER THE LAST 30 DAYS have you used LSD (acid)? (MI)	European School Survey Project on Alcohol and Drugs	NA
On how many occasions OVER THE LAST 30 DAYS have you used ecstasy (MDMA)? (MI)	European School Survey Project on Alcohol and Drugs	NA
On how many occasions OVER THE LAST 30 DAYS have you used magic mushrooms or other hallucinogens (excluding LSD)? (MI)	European School Survey Project on Alcohol and Drugs	NA
On how many occasions OVER THE LAST 30 DAYS have you used narcotics (e.g. opium, morphine, codeine)? (MI)	European School Survey Project on Alcohol and Drugs	NA
On how many occasions OVER THE LAST 30 DAYS have you used tranquillisers or sedatives (without a doctors prescription) (benzodiazepines,<br>valium or xanax; barbiturates, barbs or downers such as amytal, or seconal)? (MI)	European School Survey Project on Alcohol and Drugs	NA
On how many occasions OVER THE LAST WEEK have you used amphetamines (speed), methamphetamine (crystal meth) or desoxyn? (MI)	European School Survey Project on Alcohol and Drugs	NA
On how many occasions OVER THE LAST WEEK have you used cocaine (coke)? (MI)	European School Survey Project on Alcohol and Drugs	NA
On how many occasions OVER THE LAST WEEK have you used marijuana (grass, pot) or hashish (hash, hash oil)? (MI)	European School Survey Project on Alcohol and Drugs	NA
On how many occasions OVER THE LAST WEEK have you used ketamine (Ket, K) or Phencyclidine (PCP, or angel dust)? (MI)	European School Survey Project on Alcohol and Drugs	NA
On how many occasions OVER THE LAST WEEK have you used LSD (acid)? (MI)	European School Survey Project on Alcohol and Drugs	NA
On how many occasions OVER THE LAST WEEK have you used ecstasy (MDMA)? (MI)	European School Survey Project on Alcohol and Drugs	NA
On how many occasions OVER THE LAST WEEK have you used magic mushrooms or other hallucinogens (excluding LSD)? (MI)	European School Survey Project on Alcohol and Drugs	NA
On how many occasions OVER THE LAST WEEK have you used narcotics (e.g. opium, morphine, codeine)? (MI)	European School Survey Project on Alcohol and Drugs	NA
On how many occasions OVER THE LAST WEEK have you used tranquillisers or sedatives (without a doctors prescription) (benzodiazepines,<br>valium or xanax; barbiturates, barbs or downers such as amytal, or seconal)? (MI)	European School Survey Project on Alcohol and Drugs	NA
On how many occasions OVER THE LAST 12 MONTHS have you used amphetamines (speed), methamphetamine (crystal meth) or desoxyn? (MI)	European School Survey Project on Alcohol and Drugs	NA
On how many occasions OVER THE LAST 12 MONTHS have you used cocaine (coke)? (MI)	European School Survey Project on Alcohol and Drugs	NA

On how many occasions OVER THE LAST 12 MONTHS have you used crack? (MI)	European School Survey Project on Alcohol and Drugs	NA
On how many occasions OVER THE LAST 12 MONTHS have you used GHB or liquid ecstasy? (MI)	European School Survey Project on Alcohol and Drugs	NA
On how many occasions OVER THE LAST 12 MONTHS have you sniffed a substance (glue, aerosols etc) to get high? (MI)	European School Survey Project on Alcohol and Drugs	NA
On how many occasions OVER THE LAST 12 MONTHS have you used marijuana (grass, pot) or hashish (hash, hash oil)? (MI)	European School Survey Project on Alcohol and Drugs	NA
On how many occasions OVER THE LAST 12 MONTHS have you used ketamine (Ket, K) or Phencyclidine (PCP, or angel dust)? (MI)	European School Survey Project on Alcohol and Drugs	NA
On how many occasions OVER THE LAST 12 MONTHS have you used LSD (acid)? (MI)	European School Survey Project on Alcohol and Drugs	NA
On how many occasions OVER THE LAST 12 MONTHS have you used ecstasy (MDMA)? (MI)	European School Survey Project on Alcohol and Drugs	NA
On how many occasions OVER THE LAST 12 MONTHS have you used magic mushrooms or other hallucinogens (excluding LSD)? (MI)	European School Survey Project on Alcohol and Drugs	NA
On how many occasions OVER THE LAST 12 MONTHS have you used narcotics (e.g. opium, morphine, codeine)? (MI)	European School Survey Project on Alcohol and Drugs	NA
On how many occasions OVER THE LAST 12 MONTHS have you used tranquillisers or sedatives (without a doctors prescription) (benzodiazepines, valium or xanax; barbiturates, barbs or downers such as amytal, or seconal)? (MI)	European School Survey Project on Alcohol and Drugs	NA

***

Data File S2. Contrasts within the IMAGEN dataset and associated MSEs for estimation of pain and the severity of depressive symptoms. Grey contrasts indicate no PC selection. Orange highlights denote models chosen for hold-out center validation, representing the three with the smallest average MSEs across pain and severity of depressive symptoms among those with at least one PC selected.

**Contrasts - Anticipation**	**MSE pain**	**MSE depression**	**Contrasts - Feedback**	**MSE pain**	**MSE depression**
Anticipation_Hit_HighReward	1.0316	1.0424	Feedback_Hit_HighReward	1.0288	1.0451
Anticipation_Hit_NoReward	1.0633	1.1763	Feedback_Hit_NoReward	1.0400	1.0673
Anticipation_Hit_LowReward	1.0249	1.0461	Feedback_Hit_LowReward	1.0239	1.0477
Anticipation_Missed_HighReward	1.0508	1.1492	Feedback_Missed_HighReward	1.0290	1.0451
Anticipation_Missed_NoReward	1.0517	1.1577	Feedback_Missed_NoReward	1.0320	1.0433
Anticipation_Missed_LowReward	1.0544	1.1694	Feedback_Missed_LowReward	1.0338	1.0486
Anticipation_Hit	1.0425	1.0711	Feedback_Hit	1.0344	1.0760
Anticipation_Missed	1.0535	1.1546	Feedback_Missed	1.0342	1.0493
Anticipation_Hit > Anticipation_Missed	1.0627	1.1995	Feedback_Hit > Feedback_Missed	1.0584	1.2042

Anticipation_Hit_HighReward > Anticipation_Hit_NoReward	1.0476	1.0658	Feedback_Hit_HighReward > Feedback_Hit_NoReward	1.0648	1.1961
Anticipation_Hit_LowReward > Anticipation_Hit_NoReward	1.0528	1.1878	Feedback_Hit_LowReward > Feedback_Hit_NoReward	1.0602	1.2055
Anticipation_Missed_HighReward > Anticipation_Missed_NoReward	1.0448	1.1728	Feedback_Missed_HighReward > Feedback_Missed_NoReward	1.0507	1.1732
Anticipation_Missed_LowReward > Anticipation_Missed_NoReward	1.0516	1.1641	Feedback_Missed_LowReward > Feedback_Missed_NoReward	1.0603	1.1758
Anticipation_HighReward	1.0376	1.0441	Feedback_HighReward	1.0284	1.0445
Anticipation_LowReward	1.0505	1.1652	Feedback_LowReward	1.0283	1.0545
Anticipation_NoReward	1.0616	1.1716	Feedback_NoReward	1.0417	1.0679
Anticipation_HighReward > Anticipation_NoReward	1.0648	1.1961	Feedback_HighReward > Feedback_NoReward	1.0461	1.0647
Anticipation_LowReward > Anticipation_NoReward	1.0548	1.2056	Feedback_LowReward > Feedback_NoReward	1.0630	1.2095

***

Data File S3. Contrasts within the IMAGEN dataset and associated MSEs for estimation of pain and the severity of anxiety symptoms. Grey contrasts indicate no PC selection. Orange highlights denote models chosen for hold-out center validation, representing the three with the smallest average MSEs across pain and severity of anxiety symptoms among those with at least one PC selected.

**Contrasts - Anticipation**	**MSE pain**	**MSE anxiety**	**Contrasts - Feedback**	**MSE pain**	**MSE anxiety**
Anticipation_Hit_HighReward	1.0443	1.0234	Feedback_Hit_HighReward	1.0317	1.0232
Anticipation_Hit_NoReward	1.0643	1.0429	Feedback_Hit_NoReward	1.0643	1.0451
Anticipation_Hit_LowReward	1.0430	1.0303	Feedback_Hit_LowReward	1.0240	1.0370
Anticipation_Missed_HighReward	1.0515	1.0265	Feedback_Missed_HighReward	1.0377	1.0226
Anticipation_Missed_NoReward	1.0536	1.0301	Feedback_Missed_NoReward	1.0547	1.0260
Anticipation_Missed_LowReward	1.0536	1.0271	Feedback_Missed_LowReward	1.0545	1.0286
Anticipation_Hit	1.0486	1.0416	Feedback_Hit	1.0549	1.0560
Anticipation_Missed	1.0530	1.0264	Feedback_Missed	1.0464	1.0390
Anticipation_Hit > Anticipation_Missed	1.0610	1.0559	Feedback_Hit > Feedback_Missed	1.0581	1.0564

Anticipation_Hit_HighReward > Anticipation_Hit_NoReward	1.0639	1.0433	Feedback_Hit_HighReward > Feedback_Hit_NoReward	1.0643	1.0451
Anticipation_Hit_LowReward > Anticipation_Hit_NoReward	1.0525	1.0501	Feedback_Hit_LowReward > Feedback_Hit_NoReward	1.0575	1.0494
Anticipation_Missed_HighReward > Anticipation_Missed_NoReward	1.0447	1.0276	Feedback_Missed_HighReward > Feedback_Missed_NoReward	1.0486	1.0216
Anticipation_Missed_LowReward > Anticipation_Missed_NoReward	1.0546	1.0236	Feedback_Missed_LowReward > Feedback_Missed_NoReward	1.0450	1.0036
Anticipation_HighReward	1.0441	1.0195	Feedback_HighReward	1.0160	1.0323
Anticipation_LowReward	1.0469	1.0324	Feedback_LowReward	1.0318	1.0369
Anticipation_NoReward	1.0605	1.0416	Feedback_NoReward	1.0643	1.0451
Anticipation_HighReward > Anticipation_NoReward	1.0585	1.0394	Feedback_HighReward > Feedback_NoReward	1.0459	1.0348
Anticipation_LowReward > Anticipation_NoReward	1.0465	1.0558	Feedback_LowReward > Feedback_NoReward	1.0431	1.0212

***

Data File S4. All the selected features of the psychosocial model for the comorbidity between pain and the severity of depressive symptoms in IMAGEN. For each feature, we indicate the percentage of times it was selected (robustness), its weight for the estimation of pain, and its weight for the estimation of the severity of depressive symptoms.

**Variable**	**% times selected**	**Pain weight**	**Depression weight**
Low energy, slowed down	74	0,206699454	0,123722712
Think about how you don’t feel up to doing anything	73	0,040588732	0,224691474
Weakness	59	0,025743625	0,013146302
Think about all your shortcomings, failings, faults, mistakes	57	0,018950288	0,045853762
I am very enthusiastic about my future	54	-0,019016679	-0,064121703
Too often, when things go wrong, I get discouraged and feel like giving up	54	0,045880694	0,070495501
I am not a cheerful optimist	52	-0,013971264	0,106177524
Numbness or tingling	46	0,082420803	0,080431616
Hot or cold spells	46	0,038413477	0,039934566
Faintness or dizziness	46	0,012317762	0,008883298
Nausea, upset stomach	46	0,15427864	0,0651667
I often feel helpless and want someone else to solve my problems	46	-0,016482056	0,025127082
I concentrate easily	46	0,00095501	-0,078681039

Difficulty urinating	43	0,009790649	0,106081288
Average grade in the end of the last term you completed?	41	0,045009501	-0,066390647
Heart beating too fast	37	0,03836489	0,003076305
I am a cheerful, high-spirited person	37	-0,033469478	-0,033030872
Trouble getting breath	36	-0,000842771	0,01588233
Lump in throat	36	0,017692207	0,002473374
My family was a source of strength and support	36	-0,011275127	-0,048734283
I felt that someone in my family hated me	36	0,053092901	0,048390049
People in my family said hurtful or insulting things to me	35	0,001055395	0,002672983
Food makes you sick	33	0,054478373	0,082236794
I don’t "pay attention"	33	-0,00480085	0,039015813
Deafness (cannot hear)	31	0,029498071	-0,009201909
I dont like to waste my time daydreaming	29	-0,005875584	-0,006208291
I am self controlled	29	-2,88626E-05	1,82633E-05
Think about a recent situation, wishing it had gone better	27	-1,47457E-06	0,006475089
I am restless at the theater or lectures	24	0,08637101	0,003591382
Ran away from home - Age (MI)	22	-0,031744839	-0,031936604
Ran away from home - Since last IMAGEN	22	0,031744839	0,031936604
Most people I know like me	22	-0,000549994	0,000294791
Paralysis, muscle weakness	21	0,074090863	0,009003454
Parent abused alcohol - Age (MI)	21	-0,009738985	-0,02184458
Parent abused alcohol - Since last IMAGEN	21	0,009738985	0,02184458
Sex - Female	21	0,052178197	0,01177336
I have "racing" thoughts	17	0,000313507	0,000149745
Face broke out with pimples - Age (MI)	15	-0,033756188	0,000872042
Face broke out with pimples - Since last IMAGEN	15	0,033756188	-0,000872042
Started going out with a girlfriend/boyfriend - Feel	14	0,008364243	-0,020853554
I often feel as if I'm bursting with energy	14	-0,023205219	-0,000401167
On how many occasions IN YOUR WHOLE LIFETIME have you used ketamine (Ket, K) or Phencyclidine (PCP, or angel dust)?	14	0,026478583	0,030449265
I thought that my parents wished I had never been born	14	0,001114363	0,006320679
Went on holiday without parents - Age (MI)	12	-0,029208209	0,00903722
Went on holiday without parents - Since last IMAGEN	12	0,029208209	-0,00903722
Heavy feelings in arms, legs	11	0,003379128	-7,92406E-05
I act on the spur of the moment	11	0,0007553	0,00576326
I get easily bored when solving thought problems	11	0,005285707	0,001642238
Blurred vision	10	0,046273008	-0,001536607
Loose BM's, diarrhea	10	0,032367858	-0,024334151
I "squirm" at plays or lectures	9	0,003657125	-0,00096924
Family had money problems - Age (MI)	7	-0,01957803	-0,015072303
Family had money problems - Since last IMAGEN	7	0,01957803	0,015072303
I enjoy new and exciting experiences even if they are unconventional	7	-0,053138658	0,026075753
On how many occasions OVER THE LAST 30 DAYS have you had an alcopop to drink?	7	-0,021183912	0,031627062
Parent changed jobs - Age	6	0,023687077	-0,01083299
On how many occasions OVER THE LAST WEEK have you used cocaine (coke)? (MI)	5	0,006760172	0,001639593
On how many occasions OVER THE LAST 30 DAYS have you used cocaine (coke)? (MI)	5	0,006760172	0,001639593

When did you first try cocaine (coke)? (MI)	5	0,006760172	0,001639593
Please indicate if you have used cocaine (coke) on a weekly basis OVER A 12 MONTH PERIOD or in an excessive or problematic way IN YOUR LIFETIME? (MI)	5	0,006760172	0,001639593
Constipation	5	0,00578248	0,011155403
Went on holiday without parents - Age	5	0,030666549	-0,016203933
On how many occasions OVER THE LAST 30 DAYS have you used cocaine (coke)?	5	-0,006760172	-0,001639593
When did you first try cocaine (coke)?	5	-0,006760172	-0,001639593
How likely is it that each of the following things would happen to you personally, if you drink alcohol? Get a hangover	5	-0,030912596	0,037399689
Serious accident or illness - Age (MI)	4	-0,004648483	-0,000705039
Serious accident or illness - Since last IMAGEN	4	0,004648483	0,000705039
Met a teacher I liked a lot - Feel	4	-0,041961066	0,005145697
I save regularly	4	0,011548949	-0,001705842
Changed schools - Age (MI)	3	-0,003397702	0,002991632
Started going out with a girlfriend/boyfriend - Age	3	-0,033173617	-0,009569431
Changed schools - Since last IMAGEN	3	0,003397702	-0,002991632
I was called mean names, was made fun of, or teased in a hurtful way by a student/ peer	3	0,007406881	0,005425941
Cambridge Gambling Task - Risk Taking	3	0,025048507	-0,010152228
I change jobs	3	-0,015032607	0,01288393
Got own TV or computer - Age	2	-0,001186144	-0,016276135
On how many occasions OVER THE LAST WEEK have you used LSD (acid)? (MI)	1	1,14075E-05	-4,15872E-05
On how many occasions OVER THE LAST 30 DAYS have you used LSD (acid)? (MI)	1	1,14075E-05	-4,15872E-05
On how many occasions IN YOUR WHOLE LIFETIME have you used LSD (acid)? (MI)	1	1,14075E-05	-4,15872E-05
Please indicate if you have used LSD on a weekly basis OVER A 12 MONTH PERIOD or in an excessive or problematic way IN YOUR LIFETIME? (MI)	1	1,14075E-05	-4,15872E-05
Once I find the right way to do something, I stick to it	1	0,000536283	-0,000360269
On how many occasions OVER THE LAST 12 MONTHS have you used cocaine (coke)?	1	-0,000617863	-0,000529775
On how many occasions OVER THE LAST 30 DAYS have you used LSD (acid)?	1	-1,14075E-05	4,15872E-05
On how many occasions IN YOUR WHOLE LIFETIME have you sniffed a substance (glue, aerosols etc) to get high?	1	2,89781E-05	5,67697E-05
When did you first try LSD (acid)?	1	-1,14075E-05	4,15872E-05
Cambridge Gambling Task - Overall Proportion Bet	1	0,000756424	-0,000330899

***

Data File S5. All the selected features of the psychosocial model for the comorbidity between pain and the severity of anxiety symptoms in IMAGEN. For each feature, we indicate the percentage of times it was

selected (robustness), its weight for the estimation of pain, and its weight for the estimation of the severity of anxiety symptoms.

Variable	% times selected	Pain weight	Anxiety weight
Low energy, slowed down	62	0,229364875	0,127538208
Weakness	57	0,056283919	0,094085859
Nausea, upset stomach	47	0,167967218	0,11970362
Think about how you don’t feel up to doing anything	44	0,025782471	0,108631499
Think about all your shortcomings, failings, faults, mistakes	40	0,016522376	0,036597798
Heart beating too fast	37	0,057162021	0,047555481
Lump in throat	31	0,035993961	0,029410153
I believe that I was emotionally abused	27	-0,003958515	0,113665197
Hot or cold spells	24	0,032432358	0,038576442
I felt that someone in my family hated me	23	0,064239223	0,074043668
Think about how angry you are with yourself	22	-0,001540923	0,004102331
I am restless at the theater or lectures	21	0,117549402	0,051887183
I often have extraneous thoughts when thinking	20	0,002574784	0,092438435
Sex - Female	17	0,082367681	0,066035925
Paralysis, muscle weakness	15	0,076388411	0,004381249
I feel pleasant	15	0,017915727	-0,043422315
People in my family hit me so hard that it left me with bruises or marks	15	0,029074328	0,06303707
Numbness or tingling	12	0,027629757	0,009836588
Parents divorced - Feel	12	0,027491242	0,024960125
Think about a recent situation, wishing it had gone better	12	0,001071968	0,028083873
I have "racing" thoughts	12	0,020031424	0,025781527
Blurred vision	9	0,048656323	0,013560516
Deafness (cannot hear)	9	0,022306183	-0,001477309
Heavy feelings in arms, legs	9	0,013700918	0,011015808
Face broke out with pimples - Age (MI)	9	-0,025229431	0,001368822
Face broke out with pimples - Since last IMAGEN	9	0,025229431	-0,001368822
I often feel helpless and want someone else to solve my problems	9	-0,010281849	0,024314482
I am a cheerful, high-spirited person	9	-0,014893992	-0,010425724
I often feel as if I'm bursting with energy	9	-0,042406617	-0,01596883
I believe that I was physically abused	9	0,00388131	0,030828852
Food makes you sick	8	0,008436259	0,005147955
On how many occasions OVER THE LAST 12 MONTHS have you used tranquillisers or sedatives (without a doctors prescription) (benzodiazepines, valium or xanax; barbiturates, barbs or downers such as amytal, or seconal)? (MI)	7	0,000684823	-0,008168167
On how many occasions OVER THE LAST 30 DAYS have you used tranquillisers or sedatives (without a doctors prescription) (benzodiazepines, valium or xanax; barbiturates, barbs or downers such as amytal, or seconal)? (MI)	7	0,000684823	-0,008168167
When did you first try tranquillisers or sedatives (without a doctors prescription) (benzodiazepines, valium or xanax; barbiturates, barbs or downers such as amytal, or seconal)? (MI)	7	0,000684823	-0,008168167

Please indicate if you have used tranquillisers or sedatives (without a doctors prescription) on a weekly basis OVER A 12 MONTH PERIOD or in an excessive or problematic way IN YOUR LIFETIME? (MI)	7	0,000684823	-0,008168167
On how many occasions OVER THE LAST 12 MONTHS have you used tranquillisers or sedatives (without a doctors prescription) (benzodiazepines, valium or xanax; barbiturates, barbs or downers such as amytal, or seconal)?	7	-0,000684823	0,008168167
On how many occasions IN YOUR WHOLE LIFETIME have you used tranquillisers or sedatives (without a doctors prescription) (benzodiazepines, valium or xanax; barbiturates, barbs or downers such as amytal, or seconal)?	7	-0,000684823	0,008168167
When did you first try tranquillisers or sedatives (without a doctors prescription) (benzodiazepines, valium or xanax; barbiturates, barbs or downers such as amytal, or seconal)?	7	-0,000684823	0,008168167
Started driving a motor vehicle - Age (MI)	6	-0,006786886	0,014140152
Went on holiday without parents - Age (MI)	6	-0,022319321	0,0055224
Started driving a motor vehicle - Since last IMAGEN	6	0,006786886	-0,014140152
Went on holiday without parents - Since last IMAGEN	6	0,022319321	-0,0055224
I enjoy new and exciting experiences even if they are unconventional	6	-0,054850863	-0,015080726
Use of cocaine (coke) - over last week (MI)	5	0,006378676	-0,000460824
Use of cocaine (coke) - over last month (MI)	5	0,006378676	-0,000460824
When did you first try cocaine (coke)? (MI)	5	0,006378676	-0,000460824
Abuse of cocaine (coke). (MI)	5	0,006378676	-0,000460824
Met a teacher I liked a lot - Feel	5	-0,03505888	0,015415362
Use of cocaine (coke) - over last month	5	-0,006378676	0,000460824
When did you first try cocaine (coke)?	5	-0,006378676	0,000460824
There was nothing I wanted to change about my family	5	-0,00266123	-0,006203685
Parents divorced - Age (MI)	4	-0,00347699	-0,004748998
Went on holiday without parents - Age	4	0,036475409	0,024394345
Started going out with a girlfriend/boyfriend - Age	4	-0,041537442	-0,020434238
Decided about college / university - Age	4	-0,011373562	-0,011444698
Parents divorced - Since last IMAGEN	4	0,00347699	0,004748998
Most people I know like me	4	-0,023195937	0,008596196
Loose BM's, diarrhea	3	0,013550765	0,005737109
Ran away from home - Age (MI)	3	-0,009514131	-0,002299395
Parents argued or fought - Age (MI)	3	-0,001339027	-0,003211981
Ran away from home - Since last IMAGEN	3	0,009514131	0,002299395
Parents argued or fought - Age	3	-0,000308446	-0,019140427
Parents argued or fought - Since last IMAGEN	3	0,001339027	0,003211981
I strive for excellence in everything I do	3	0,00899047	0,018827675
Cambridge Gambling Task - Risk Taking	3	0,030392385	-0,009266049
Trouble walking	2	0,006575348	0,0024017
Serious accident or illness - Age (MI)	2	-0,003453644	-0,001205385
Family had money problems - Age (MI)	2	-0,005733168	0,002252348
Changed schools - Age (MI)	2	-0,003376419	0,000865886
Parent abused alcohol - Feel	2	0,011862622	0,008183046
Serious accident or illness - Since last IMAGEN	2	0,003453644	0,001205385
Family had money problems - Since last IMAGEN	2	0,005733168	-0,002252348

Changed schools - Since last IMAGEN	2	0,003376419	-0,000865886
Face broke out with pimples - Age	2	0,012577729	-0,0027253
Too often, when things go wrong, I get discouraged and feel like giving up	2	0,000272352	0,000342701
Cambridge Gambling Task - Quality of Decision Making	2	0,003255055	0,010970856
Found religion - Age (MI)	1	0,000174286	-0,000111734
Found religion - Since last IMAGEN	1	-0,000174286	0,000111734
Parent changed jobs - Age	1	0,002475825	-0,000342508
Started seeing a therapist - Feel	1	-1,34636E-06	6,11217E-06
Got in trouble with the law - Age	1	-1,27587E-05	-0,000231092
I have bullied a family member	1	-8,40579E-05	1,4111E-05
I have bullied a teacher	1	-0,004900959	0,006540379
Cambridge Gambling Task - Deliberation Time	1	-0,006323225	-0,002167933
I change jobs	1	-0,000626368	0,000389255
I save regularly	1	0,001993657	-0,000140718

References

[1] Arnett PA, Newman JP. Gray's three-arousal model: an empirical investigation. Personal Individ Differ 2000;28:1171–1189. https://doi.org/10.1016/S0191-8869(99)00169-5.

[2] Bernstein DP, Stein JA, Newcomb MD, Walker E, Pogge D, Ahluvalia T, Stokes J, Handelsman L, Medrano M, Desmond D, Zule W. Development and validation of a brief screening version of the Childhood Trauma Questionnaire. Child Abuse Negl 2003;27:169–190. https://doi.org/10.1016/s0145-2134(02)00541-0.

[3] Cosgrove KT, McDermott TJ, White EJ, Mosconi MW, Thompson WK, Paulus MP, Cardenas-Iniguez C, Aupperle RL. Limits to the generalizability of resting-state functional magnetic resonance imaging studies of youth: An examination of ABCD Study® baseline data. Brain Imaging Behav 2022;16:1919–1925. https://doi.org/10.1007/s11682-022-00665-2.

[4] Costa PT, McCrae RR. Normal personality assessment in clinical practice: The NEO Personality Inventory. Psychol Assess 1992;4:5–13. https://doi.org/10.1037/1040-3590.4.1.5.

[5] Dadi K, Varoquaux G, Machlouzarides-Shalit A, Gorgolewski KJ, Wassermann D, Thirion B, Mensch A. Fine-grain atlases of functional modes for fMRI analysis. NeuroImage 2020;221:117126. https://doi.org/10.1016/j.neuroimage.2020.117126.

[6] Goodman R, Ford T, Richards H, Gatward R, Meltzer H. The Development and Well-Being Assessment: description and initial validation of an integrated assessment of child and adolescent psychopathology. J Child Psychol Psychiatry 2000;41:645–655.

[7] Hibell B, Andersson B, Bjarnason T, Kokkevi A, Morgan M, Narusk A, Ahlström S. The 1995 ESPAD report. Alcohol and other drug use among students in 26 European countiries. 1997.

[8] Kirby KN, Petry NM, Bickel WK. Heroin addicts have higher discount rates for delayed rewards than non-drug-using controls. J Exp Psychol Gen 1999;128:78–87. https://doi.org/10.1037//0096-3445.128.1.78.

[9] Meesters C, Muris P, Ghys A, Reumerman T, Rooijmans M. The Children's Somatization Inventory: further evidence for its reliability and validity in a pediatric and a community sample of Dutch children and adolescents. J Pediatr Psychol 2003;28:413–422. https://doi.org/10.1093/jpepsy/jsg031.

[10] Newcomb MD, Huba GJ, Bentler PM. A Multidimensional Assessment of Stressful Life Events among Adolescents: Derivation and Correlates. J Health Soc Behav 1981;22:400–415. https://doi.org/10.2307/2136681.

[11] Obozinski G, Taskar B, Jordan MI. Multi-task feature selection. University of California, Berkeley, 2006.

[12] Parkes L, Fulcher B, Yücel M, Fornito A. An evaluation of the efficacy, reliability, and sensitivity of motion correction strategies for resting-state functional MRI. NeuroImage 2018;171:415–436. https://doi.org/10.1016/j.neuroimage.2017.12.073.

[13] Patton JH, Stanford MS, Barratt ES. Factor structure of the Barratt impulsiveness scale. J Clin Psychol 1995;51:768–774. https://doi.org/10.1002/1097-4679(199511)51:6<768::aid-jclp2270510607>3.0.co;2-1.

[14] Pruim RHR, Mennes M, Buitelaar JK, Beckmann CF. Evaluation of ICA-AROMA and alternative strategies for motion artifact removal in resting state fMRI. NeuroImage 2015;112:278–287. https://doi.org/10.1016/j.neuroimage.2015.02.063.

[15] Treynor W, Gonzalez R, Nolen-Hoeksema S. Rumination Reconsidered: A Psychometric Analysis. Cogn Ther Res 2003;27:247–259. https://doi.org/10.1023/A:1023910315561.

[16] Walker LS, Beck JE, Garber J, Lambert W. Children's Somatization Inventory: psychometric properties of the revised form (CSI-24). J Pediatr Psychol 2009;34:430–440. https://doi.org/10.1093/jpepsy/jsn093.

[17] Woicik PA, Stewart SH, Pihl RO, Conrod PJ. The Substance Use Risk Profile Scale: a scale measuring traits linked to reinforcement-specific substance use profiles. Addict Behav 2009;34:1042–1055. https://doi.org/10.1016/j.addbeh.2009.07.001.
